# Genetic architecture underlying the lignin biosynthesis pathway involves noncoding RNAs and transcription factors for growth and wood properties in *Populus*

**DOI:** 10.1111/pbi.12978

**Published:** 2018-07-16

**Authors:** Mingyang Quan, Qingzhang Du, Liang Xiao, Wenjie Lu, Longxin Wang, Jianbo Xie, Yuepeng Song, Baohua Xu, Deqiang Zhang

**Affiliations:** ^1^ Beijing Advanced Innovation Center for Tree Breeding by Molecular Design Beijing Forestry University Beijing China; ^2^ National Engineering Laboratory for Tree Breeding College of Biological Sciences and Technology Beijing Forestry University Beijing China; ^3^ Key Laboratory of Genetics and Breeding in Forest Trees and Ornamental Plants Ministry of Education College of Biological Sciences and Technology Beijing Forestry University Beijing China

**Keywords:** association genetics, epistasis, eQTN mapping, lignin biosynthesis pathway, long noncoding RNA, miRNA, *Populus*, transcription factor

## Abstract

Lignin provides structural support in perennial woody plants and is a complex phenolic polymer derived from phenylpropanoid pathway. Lignin biosynthesis is regulated by coordinated networks involving transcription factors (TFs), microRNAs (miRNAs) and long noncoding RNAs (lncRNAs). However, the genetic networks underlying the lignin biosynthesis pathway for tree growth and wood properties remain unknown. Here, we used association genetics (additive, dominant and epistasis) and expression quantitative trait nucleotide (eQTN) mapping to decipher the genetic networks for tree growth and wood properties in 435 unrelated individuals of *Populus tomentosa*. We detected 124 significant associations (*P *≤* *6.89E‐05) for 10 growth and wood property traits using 30 265 single nucleotide polymorphisms from 203 lignin biosynthetic genes, 81 TF genes, 36 miRNA genes and 71 lncRNA loci, implying their common roles in wood formation. Epistasis analysis uncovered 745 significant pairwise interactions, which helped to construct proposed genetic networks of lignin biosynthesis pathway and found that these regulators might affect phenotypes by linking two lignin biosynthetic genes. eQTNs were used to interpret how causal genes contributed to phenotypes. Lastly, we investigated the possible functions of the genes encoding 4‐coumarate: CoA ligase and cinnamate‐4‐hydroxylase in wood traits using epistasis, eQTN mapping and enzymatic activity assays. Our study provides new insights into the lignin biosynthesis pathway in poplar and enables the novel genetic factors as biomarkers for facilitating genetic improvement of trees.

## Introduction

Lignin is a phenylpropanoid‐derived phenolic polymer abundant in vascular plants and is a component of the secondary cell wall (Bonawitz and Chapple, 2010). Lignin provides mechanical strength and hydrophobicity to cell walls, enabling trees to grow to great heights and transport water and nutrients over long distances, and plays essential roles in protection from pathogens (Bhuiyan *et al*., 2009; Ros, 1997). Lignin is also one of the main components of wood, which is the substantial character of perennial woody plants and provides the raw materials for industrial products and renewable energy (Novaes *et al*., 2010).

The biosynthesis of the lignin monomer starts with the deamination of phenylalanine, resulting in the synthesis of three monolignols: coniferyl, sinapyl and *p*‐coumaryl alcohols. In dicots, such as *Populus*, lignin polymers are composed of guaiacyl (G), syringyl (S) and low levels of *p*‐hydroxyphenyl (H) units, which are processed from the three monomers, respectively (Campbell and Sederoff, 1996; Voxeur *et al*., 2015). Some key genes in lignin biosynthesis pathway regulate the lignin content in dicots. For example, down‐regulation of *4CL* (4‐coumarate: CoA ligase) in hybrid poplar (*P. tremula* × *P. alba*) sharply decreased the amount of lignin and largely changed wood chemistry and wood metabolism (Voelker *et al*., 2010). Furthermore, down‐regulation of *C4H* (cinnamate‐4‐hydroxylase) in transgenic tobacco reduced the phenylalanine ammonia‐lyase (PAL) enzymatic activity by feedback modulation (Blount *et al*., 2000); phenylalanine concentration also increased the expression of *PAL*,* 4CL*,* CCoAOMT* (caffoyl‐CoA *O*‐methyltransferase) and *CCR* (cinnamoyl‐CoA reductase) in *Pinus taeda* (Anterola *et al*., 2002). While these studies demonstrated the interactions of multiple lignin biosynthetic genes, other aspects of this network require further study, such as the patterns of genetic interaction within the lignin biosynthesis pathway and how the multigene coordinated network functions in wood formation.

Notably, growing evidence suggests that lignin biosynthesis pathway is regulated by various upstream genetic factors. Transcription factors (TFs) have important functions in the regulation of lignin biosynthesis. The presence of AC elements in the promoters of many lignin biosynthetic genes of *Pinus teada*, which can be recognized by MYB TFs, provides evidence of the involvement of common TFs in the regulation of lignin biosynthesis (Patzlaff *et al*., 2003). Recently, regulation by noncoding RNAs (ncRNAs), such as microRNAs (miRNAs) and long noncoding RNAs (lncRNAs), has also attracted considerable attention. For example, overexpression of miR397a in *P. trichocarpa* down‐regulated the expression of 17 *laccase* (*LAC*) genes, resulting in a reduction in lignin content (Lu *et al*., 2013). In *P. tomentosa*, eight lncRNAs exhibited epistatic effects with 15 phenylpropanoid biosynthesis genes, which contributed to the regulation of biomass in trees (Zhou *et al*., 2017). Therefore, to improve our understanding of the lignin biosynthesis pathway in *Populus*, it is critical to uncover the broad‐spectrum regulatory networks involving ncRNAs (lncRNAs and miRNAs) and TFs underlying various wood formation phenotypes and to ascertain the specific functions of each genetic factor in lignin biosynthesis.

Association mapping is an excellent strategy for examining the allelic variants behind the complex quantitative traits of perennial trees with large population sizes and abundant phenotypic variation (Beaulieu *et al*., 2011; Guerra *et al*., 2013; Wegrzyn *et al*., 2010). Single nucleotide polymorphism (SNP)‐based association mapping has been used to characterize the SNPs within specific lignin biosynthetic genes for wood properties in trees (Thumma *et al*., 2005). Association studies underlying additive, dominant and epistatic interactions have become a powerful method for deciphering the genetic architecture of multigene networks (Deng *et al*., 2017; Du *et al*., 2015; Mackay, 2014). However, there is a substantial gap in our knowledge of how the causative genes identified from association mapping contribute to traits. This gap can be addressed with expression quantitative trait nucleotide (eQTN) mapping, which is used to decipher the allelic variations that contribute to phenotypes at the transcriptional level, and has been applied in deciphering the genetic architecture of quantitative traits in maize (Li *et al*., 2013; Wen *et al*., 2014). Thus, association studies and eQTN mapping can be combined to uncover the genetic factors (miRNAs, lncRNAs, TFs and protein‐coding genes) underlying the lignin biosynthesis pathway, which offers effective clues for understanding the comprehensive genetic networks and identifying causal genes for wood property traits.

Here, we identified potential ncRNAs (lncRNAs and miRNAs) and TFs that are associated with 203 lignin biosynthetic genes in *P. tomentosa*. Using association studies, we deciphered the genetic basis (additive, dominant and epistatic effects) of these genetic factors underlying lignin biosynthesis for tree growth and wood properties in a natural population of 435 unrelated individuals of *P. tomentosa*. Based on this, we determined the multigenotype combinations for lignin content and proposed genetic networks in the lignin biosynthesis pathway, which will be useful for selection breeding. eQTN mapping combined with analyses of enzymatic activity aided our interpretation of the causal genes identified in association studies for tree growth and wood properties. The strategy of integrating association mapping (additive, dominant and epistasis) with eQTN analysis offers an alternative idea for deciphering the genetic regulatory mechanisms of complex traits in perennial trees. Our findings provide new insight into the lignin biosynthesis pathway in poplar and may enhance the efficiency of tree breeding.

## Results

### Identification and expression analysis of candidate genetic factors in lignin biosynthesis pathway in *P. tomentosa*

#### Expression patterns of candidate lignin biosynthetic genes in P. tomentosa

We identified 298 genes from 13 gene families in lignin biosynthesis pathway in *P. trichocarpa* genome: *PAL*,* 4CL*,* LAC*,* CCR*,* C4H*,* CCoAOMT*,* CSE* (caffeoyl shikimate esterase), *CAD* (cinnamyl alcohol dehydrogenase), *C3H* (4‐coumarate 3‐hydroxylase), *F5H* (ferulate/coniferaldehyde 5‐hydroxylase), *PO* (peroxidase), *HCT* (*p*‐hydroxycinnamoyl‐CoA:quinate shikimate *p*‐hydroxycinnamoyltransferase) and *COMT* (caffeic acid/5‐hydroxyconiferaldehyde *O*‐methyltransferase). RNA‐seq was used to examine the transcript abundance of the 298 lignin biosynthetic genes, which found that 203 genes expressed in at least one tested tissue of *P. tomentosa*, that is leaf, developing xylem, mature xylem and cambium, with diverse patterns (Data S1). For example, all *CSE* and *PAL* family members were highly expressed in stem tissues (developing xylem, mature xylem and cambium). Genes in the *C4H*,* CCR*,* F5H*,* CCoAOMT* and *C3H* families exhibited low to moderate expression levels in the tested tissues. Some genes showed tissue‐specific expression; for example, *Ptr‐COMT27* was preferentially expressed in mature xylem but had no expression in the leaf; 70.94% of the lignin biosynthetic genes had higher expression levels in at least one stem tissue than in leaf (Data S1), indicating their potential functions in wood formation.

#### Identification of lncRNAs and miRNAs for lignin biosynthetic genes

To investigate the potential ncRNAs involved in the lignin biosynthesis pathway, we identified 296 lncRNA–mRNA pairs representing 190 lncRNAs and 73 lignin biosynthetic genes in *P. tomentosa* (Data S2). Of which, 59.05% of the pairs were *cis*‐acting and 45 of the lncRNAs had both *cis* and *trans* roles. Expression analysis identified 47 lncRNAs only expressed in one tissue (Data S3). Expression correlations of 296 lncRNA‐mRNA pairs showed that 76.69% of the pairs exhibited significantly strong correlations (*r *>* *0.4 or <−0.4, *P *<* *0.01, Table S1), indicating the close regulatory relationship between these 190 lncRNAs and lignin biosynthetic genes. Additionally, degradome sequencing showed that 36 miRNAs from 17 miRNA families regulate 31 lignin biosynthetic genes in *P. tomentosa* (Data S4). The expression of these 36 miRNAs varied across the six tissues (Data S5), and the expression correlations for 69 miRNA–mRNA pairs revealed that 54 pairs were significantly negatively correlated (−0.960 to −0.189, *P *<* *0.01, Table S2), strongly supporting the roles of miRNAs in regulation of lignin biosynthetic genes.

#### Cis‐regulatory motif analysis identified the shared TFs regulating lignin biosynthetic genes

To explore the potential TFs that regulate the lignin biosynthetic genes, 26 TF binding motifs were over‐represented in >90% of the lignin biosynthetic gene promoters, and every promoter contained 1 to 297 TF binding sites (TFBS) (mean of 121) (Table S3 and Data S6), which suggested the universal regulatory mechanism of TFs for lignin biosynthesis, in contrast to ncRNAs, which affect only a subset of genes. Examination of the motif position showed that most were evenly distributed across the 203 lignin biosynthetic gene promoters, except for two TFBS of the AT‐Hook family (TFBS_0131 and TFBS_0148), which were preferentially located 1 kb upstream of the transcription start site (TSS) (frequency > 0.1; Figure S1). Correspondingly, 81 TF genes from eight TF families (HD‐ZIP, Myb/SANT, AT‐Hook, TCR, TBP, bZIP, bHLH and C2H2) interacted with the 26 enriched motifs (Table S3). Expression correlations of 15 246 TF‐gene pairs revealed that 62.47% were strongly correlated (*r *>* *0.4 or <−0.4, *P *<* *0.01) (Data S7 and Data S8), revealing their potential regulatory roles for lignin biosynthetic genes.

The expression profiles of 40 genes, selected from 13 gene families, were validated by real‐time quantitative PCR (RT‐qPCR), which showed consistent patterns with the RNA‐seq data (*r *=* *0.91, *P *<* *0.01; Figure S2a–b). Additionally, six lncRNAs, six miRNAs and nine TFs which revealed significant expression correlations with the 40 genes by RNA‐seq were chosen for RT‐qPCR validation, results showed a nearly perfect correlation with the RNA‐seq results for the expression profiles and expression correlations of lncRNA/miRNA/TF–mRNA pairs (*r *=* *0.85, 0.64, and 0.71, respectively, *P *<* *0.01, Figure S2c–d). These findings supported that candidate ncRNAs and TFs have extensive regulatory roles in lignin biosynthesis pathway.

### Genetic diversity assessment within candidate genes and linkage disequilibrium (LD) tests in the association population of *P. tomentosa*

According to the genomic resequencing data (coverage *> *15×) for the 435 unrelated individuals of *P. tomentosa*, we detected 35 161 SNPs from all candidate genes of the lignin biosynthesis pathway, including 203 biosynthesis genes, 81 TF genes, 36 miRNA genes and 71 lncRNA loci. Of these, 30 265 high‐quality SNPs (minor allele frequency (MAF) > 5% and missing data < 10%) were selected for further analysis (Data S9–S12). Of the high‐quality SNPs, 7451 were in ncRNA genes, and the remaining 22 814 were derived from protein‐coding genes, with 81.92% in noncoding regions, revealing the high nucleotide diversity in noncoding sequences. The SNP frequency varied across genes with one SNP per 15–659 bp (Table 1 and Table S4). Notably, the lignin biosynthetic genes had the highest nucleotide diversity (average π = 0.0428), followed by lncRNA loci (average π = 0.0419) and TFs (average π = 0.0372), with miRNA genes being the most conserved (average π = 0.0356). The nucleotide diversity in noncoding regions (π = 0.0654) of protein‐coding genes was higher than that in coding regions (π = 0.0318), indicating that coding regions were under stronger selection pressure. Moreover, nonsynonymous (*d*_N_) diversity was lower than synonymous (*d*_S_) diversity (*d*_N_ */d*_S_ **= 0.43), indicating purifying selection within the exons. The *r* ^2^ (squared allele‐frequency correlations) of all pairwise combinations, combined with their physical distance, were used to evaluate the overall patterns of LD for each chromosome. We detected ~2400 high‐LD blocks (*r* ^2^ > 0.75, *P *<* *0.01) across 19 chromosomes and the *r* ^2^ dropped to 0.1 within ~0.6 kb to ~1.2 kb on average (Figure S3‐S4).

**Table 1:** Summary of single nucleotide polymorphisms (SNPs) within candidate genes in our studies

Category	Gene numbers	SNP numbers^a^	Frequency	π	θw
Lignin biosynthetic genes	201	18560/16350	22–659	0.00081–0.12219	0.01322–0.19865
Transcription factor genes	81	8194/6464	31–372	0.00907–0.09756	0.02550–0.19074
MiRNA genes	32	1218/884	15–258	0.00048–0.12435	0.00950–0.20393
LncRNA loci	71	7189/6567	27–303	0.00151–0.11978	0.01345–0.16771

a Number of total SNPs and high‐quality SNPs in each panel, respectively (separated by a semicolon).

### Genetic basis of natural variants for tree growth and wood property traits in *P. tomentosa*

To identify the causative variants among the candidate genetic factors of the lignin biosynthesis pathway for wood formation in *P. tomentosa*, 10 tree growth and wood property traits were measured for all individuals in the association population, that is diameter at breast height (DBH), tree height (H), stem volume (V), fibre width (FW), fibre length (FL), microfibril angle (MFA), lignin content (LC), holocellulose content (HC), α‐cellulose content (CC) and hemicellulose content (HEC). Collectively, 124 associations representing 116 significant loci were identified at *P *≤* *6.89E‐05 (*P *=* *1/n, a Bonferroni correction) (Figure S5 and Table 2). The phenotypic variation explained (*R* ^2^) by each association was 13.14%–31.30%, with an average of 21.40% (Table S5).

**Table 2:** Summary of causal SNPs within candidate genes associated with growth and wood properties in the association population of *P. tomentosa*

Trait	Number of associations	SNP numbers	Independent SNP numbers	The source of significant SNPs				Additive effect	Dominant effect	*R* ^2^ (%)
				Lignin biosynthetic genes	Transcription factor genes	MiRNA gene	LncRNA loci			
DBH	27	27	26	9	6	4	8	0.090–7.913	−4.222–8.645	20.59–26.85
H	3	3	2	3	0	0	0	1.436–2.194	−4.009–2.762	20.17–22.41
V	10	10	10	3	2	1	4	1.497–30.608	−31.147–33.557	21.14–23.57
MAF	10	10	4	0	7	0	3	0.696–1.546	−5.573–5.569	18.32–26.64
FW	31	27	21	22	4	0	5	0.262–3.49	−1.703–4.541	17.62–25.48
FL	21	19	13	12	4	0	5	0.001–0.099	−0.328–0.057	15.39–27.36
CC	1	1	1	0	1	0	0	2.822	–	17.45
HC	11	11	10	6	4	0	1	0.514–12.967	−20.164–22.254	18.19–31.3
HEC	1	1	1	0	1	0	0	13.981	11.088	22.80
LC	9	9	8	4	2	0	3	0.296–2.101	−1.021–3.564	13.14–20.65

The 116 significant loci distributed across 85 candidate genes, and 96 of them were independent loci (weak or no LD, *r* ^2^ < 0.75 or different chromosomes). For the 34 associations identified in ncRNA genes, 15 loci overlapped with transcribed regions of lncRNAs, and five were in miRNA genes with one in the precursor miRNA (pre‐miRNA) region (Pto‐MIR397a_SNP7 for DBH; Figure S6a). Of the 90 loci identified in protein‐coding genes, 11 were in exons, such as Ptr‐HCT12_SNP59 (G/C), which caused a nonsynonymous mutation of Ala to Pro, and had the peak signal for H (*P* = 1.29E‐05) (Figure S6b). We found that 59 loci in lignin biosynthetic genes were associated with seven traits, and 42 were in promoters, such as Ptr‐4CL12_SNP28 overlapped with TFBS_0323 of Myb/SANT, which was associated with FL (*P* = 1.21E‐10) (Figure S6c). These results supported that candidate TFs and ncRNAs have functions in wood formation. Each trait associated with 1–27 loci from four categories of genes, indicating their common roles in tree growth and wood properties. For example, 27 significant SNPs, including eight in lncRNA loci, four in miRNA genes, nine in lignin biosynthetic genes, and six in TF genes, were associated with DBH with *R* ^2^ of 20.59%–26.85% (Figure S6a).

The associated loci exhibited various effects on traits. For the 124 locus–trait associations, 100 and 97 associations possessed additive and dominant effects, respectively, and 58.87% of associations had joint additive and dominant effects (Table S5). Twelve pleiotropic genes, including three lncRNA loci, one miRNA gene, four lignin biosynthetic genes and four TF genes, shared two to three traits, and they showed different effects on traits, indicating their common roles in wood formation. Seven pleiotropic genes possessed combined additive and dominant effects for all associated traits, such as *Pto‐MIR167a* for DBH and V. Notably, one locus had different contributions to different traits. Ptr‐CAD13_SNP44 possessed joint effects for HC and V; however, it showed distinctly dominant effects for HC (12.160) and V (−30.827). Additionally, the pleiotropic genes could have either an additive or dominant effect for traits. For example, *Ptr‐TBP2* showed additive effects for FL, but dominant effects for HC. Additionally, the pleiotropic genes could show combined effects for one trait, and a single effect to other traits. For instance, SNPs in *L40* (lncRNA locus 40) showed combined additive and dominant effects for LC, but only displayed varied additive effects for MFA (0.696–1.546) (Table S5). These findings indicated the diverse functions of the four types of genetic factors for specific traits related to wood formation.

**Figure 1:**
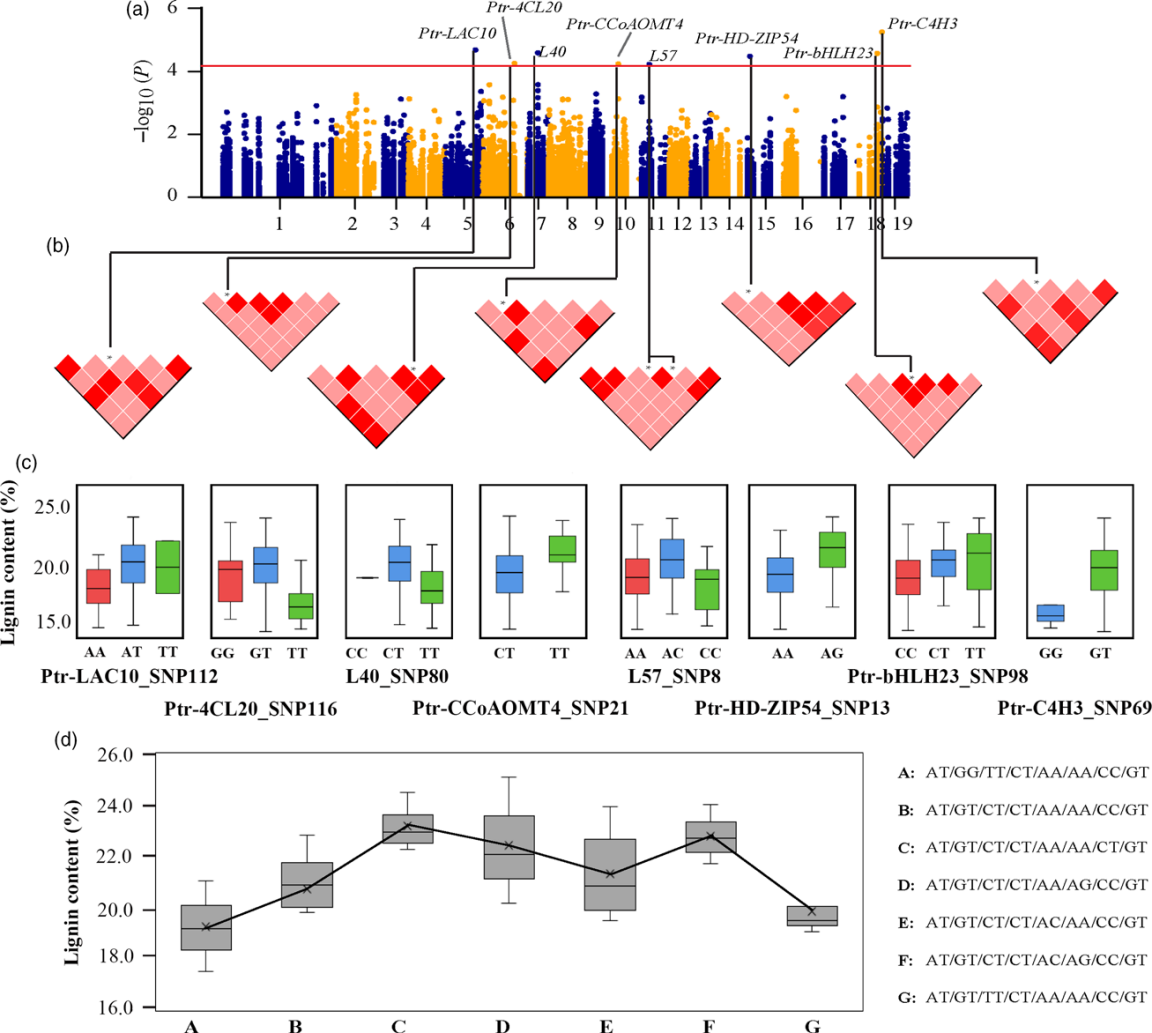
The proposed multi‐SNP‐based genotype combinations for lignin content. (a) Manhattan plot for lignin content marked with the eight causal genes whose significant SNPs simultaneously associated with lignin content. (b) Pairwise LD plots among multiple loci around each causal SNP; the pink and red blocks represent the *r* ^2^ of two SNPs that were less than and greater than 0.75, respectively. (c) Genotype effects of each causal SNP for lignin content. (d) Seven possible genotype combinations with a frequency of ≥5% from the eight allelic variations, and the genotype combination effects for lignin content in the association population of *P. tomentosa*. The SNPs in each genotype combination were ordered according to (c). We used SNP8 in L57, as L57‐SNP8/9 were in LD.

Based on the effects of significant loci for a trait, the possible genotype combinations of significant SNPs for the same trait were identified. Eight independent SNPs were significantly associated with LC (Figure 1a–b), which different genotypes of them contributed differently to LC (Figure 1c), and they displayed seven possible common genotype combinations (frequency ≥ 5%, *P *<* *0.01) for LC (Figure 1d). The genotype alternation of three major loci (Ptr‐4CL20_SNP116, L40_SNP80 and Ptr‐bHLH23_SNP98) led to phenotypic differences of LC, where GT‐CT‐CT and GG‐TT‐CC combinations of the three major loci represented the maximum (23.16%) and minimum (19.23%) phenotypic values, respectively.

### Pairwise epistasis between candidate loci revealed complex genetic networks in the lignin biosynthesis pathway

**Figure 2:**
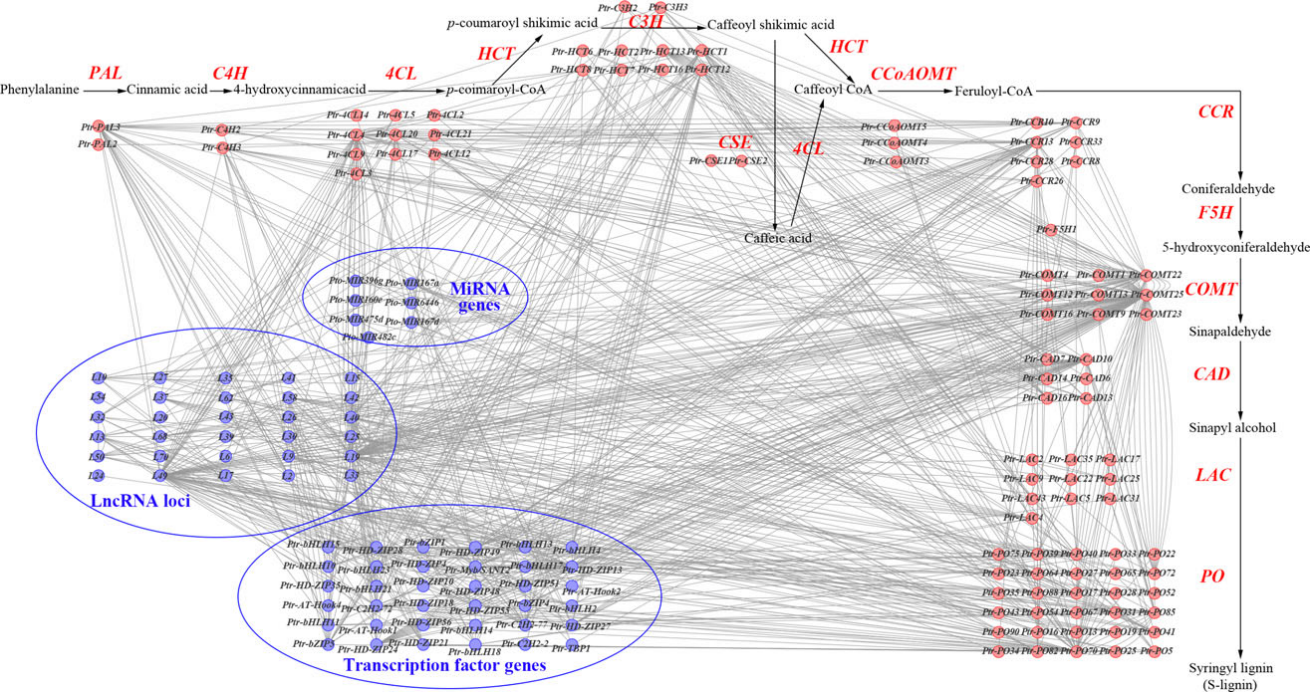
The proposed genetic interactive network among genetic factors in lignin biosynthesis pathway, which exemplified by S‐lignin biosynthesis. The red circles represents the lignin biosynthetic genes and blue circles represents lncRNAs, miRNAs and TFs that had epistatic interactions with lignin biosynthetic genes. The blue circles marked ‘L’ depict the lncRNA locus.

**Figure 3:**
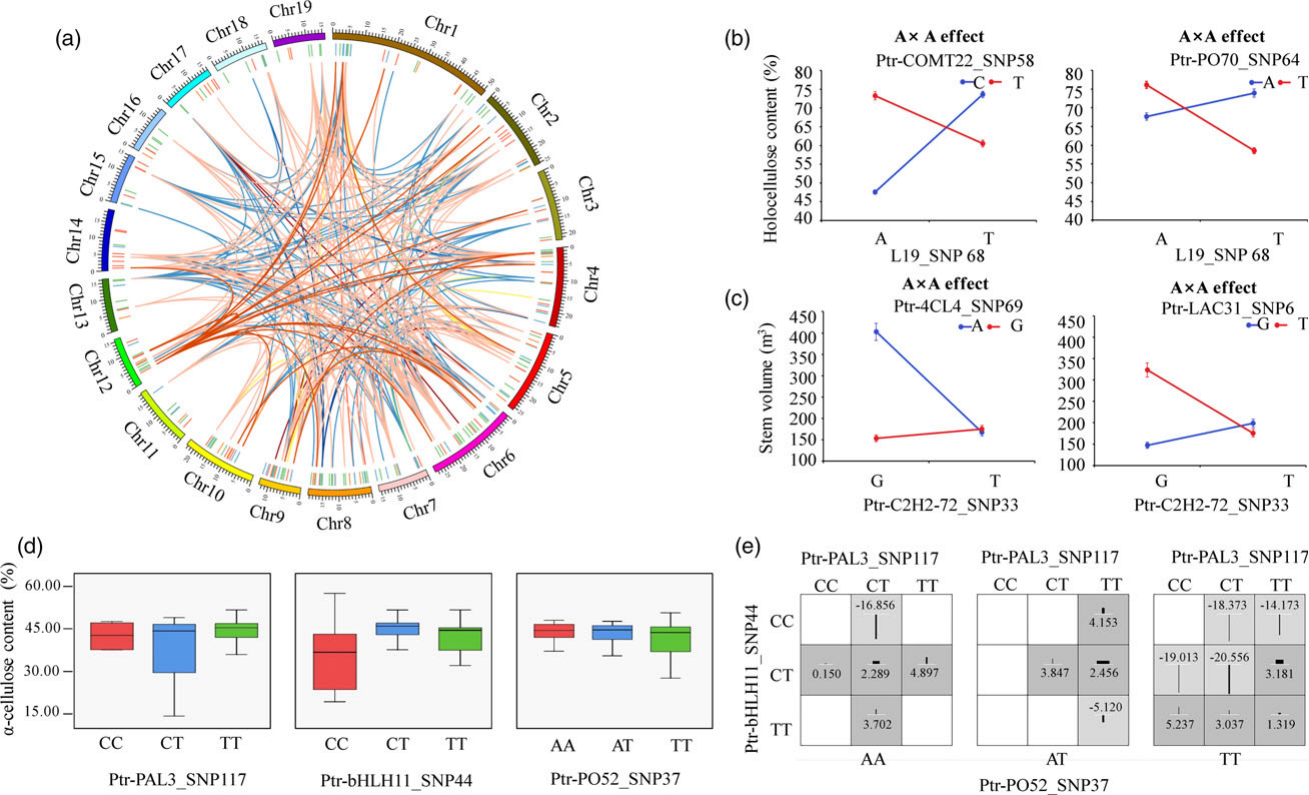
The epistatic interaction networks of alleles within candidate genes related to the lignin biosynthesis pathway. (a) The circular diagram shows 745 pairwise interactions for ten growth and wood property traits (*P *≤* *1.0E‐04). The outer circle represents the chromosomes. The middle circle indicates the chromosome position of lignin biosynthetic genes (green), lncRNA loci (blue), miRNA genes (orange) and TF genes (red). The interior lines represents the pairwise interactions, and different colour lines represent different traits, (deep red, light blue, pink, orange, yellow, green, deep blue, red, blue and deep orange indicate diameter at breast height, tree height, stem volume, fibre length, fibre width, microfibril angle, lignin content, α‐cellulose content, holocellulose content and hemicellulose content, respectively). (b) The epistatic effects for holocellulose content between L19_SNP68 with two loci in *Ptr‐COMT22* and *Ptr‐PO70*, respectively. (c) The epistatic effects for stem volume between Ptr‐C2H2‐72_SNP33 with two loci in *Ptr‐4CL4* and *Ptr‐LAC31*, respectively. (d) Box plots revealing the single SNP genotype effects of three SNPs for α‐cellulose content (CC) and (e) epistatic effects of different genotypic combination effects for CC.

To decipher the genetic networks in the lignin biosynthesis pathway, we chose 6313 loci that were significant in SNP‐based associations at *P *<* *0.01 and tested the epistatic interactions between each SNP pair for each trait. Collectively, 745 significant pairwise epistatic combinations were detected for 10 tree growth and wood property traits at *P *<* *1.0 × 10^−4^. Based on this, we constructed a proposed epistatic network among genetic factors in lignin biosynthesis pathway, which exemplified by S‐lignin biosynthesis (Figure 2), providing useful resource for the genetic interactions within the pathway. The 745 SNP pairs represented 395 loci within 167 genes (Figure 3a and Table S6), including 166, 188 and 23 epistatic interactions for TFs, lncRNAs and miRNAs with lignin biosynthetic genes, respectively (Figure 2 and Figure 3a). Interestingly, 169 SNP–SNP pairs were between ncRNA/TF genes and their corresponding target genes. Additionally, 52 significant genes were repeatedly detected with epistatic effects (507 pairwise), including 11 causative variants with additive/dominant effects. For example, Ptr‐bHLH11_SNP45 (G/A) showed a joint effects of additive, dominant and epistasis. Additionally, 68.29% (267) of the loci showed epistatic interactions with multiple SNPs and 50 SNP–SNP pairs associated with more than one trait. For example, Ptr‐CCR13_SNP7 (G/A), a nonsynonymous mutation of Gly to Arg, had 28 pairwise combinations with 22 SNPs for four traits. The Ptr‐CCR13_SNP7‐L19_SNP33 interaction was associated with D, V and FW, in which the different genotype combinations provided substantial epistatic effects than single locus for the traits (Figure S7).

Among the 745 epistatic interactions, 257 additive × additive (AA), 166 additive × dominance (AD), 189 dominance × additive (DA) and 133 dominance × dominance (DD) interaction effects were partitioned for 10 traits, indicating the diverse interaction models for traits (Table S6). Interestingly, we found that ncRNA/TF genes linked two lignin biosynthetic genes in the pathway (Figure 2). For example, L19_SNP68 had epistatic interactions with *COMT‐22* (Ptr‐COMT22_SNP58) and *PO‐70* (Ptr‐PO70_SNP64), in which the different allelic interactions showed different effects for HC (Figure 3b). Moreover, transcription factor *C2H2* (Ptr‐C2H2‐72_SNP33) linked *4CL* (Ptr‐4CL4_SNP69) and *LAC* (Ptr‐LAC31_SNP6) together, which had no direct genetic or regulatory interactions within the pathway, and the allelic combinations showed considerable nonadditive effects for V (Figure 3c). These results demonstrated that, except for the direct interaction among lignin biosynthetic genes, ncRNAs and TFs participated in alternative interactions among genes. We also found that the genotype combinations of SNPs with epistatic interactions for phenotypes displayed stronger effects than single loci. For example, CC varied across different genotype combinations (Ptr‐PO52_SNP37, Ptr‐bHLH11_SNP44 and Ptr‐PAL3_SNP117), and phenotypic differences ranged from ‐20.556 (TT‐CT‐CT) to 5.237 (TT‐TT‐CC), which differed from single‐locus effects (Figure 3d–e). These findings revealed that epistatic interactions involving miRNAs, lncRNAs and TFs enriched the lignin biosynthesis pathway and proposed alternative interaction models connecting lignin biosynthetic genes, thereby substantially affecting growth and wood properties of *P. tomentosa*.

### Genetic regulation of gene expression accounts for a substantial proportion of phenotypic variations in P. tomentosa

To investigate the causative allelic variants underlying the level of transcription of lignin biosynthetic genes, eQTN mapping was conducted between 30 265 genetic variants and the expression levels of 74 lignin biosynthetic genes (expressed in ≥80% of the 435 individuals; Data S13). At *P *≤* *6.89E‐05, we found that 42 lignin biosynthetic genes defined 20 558 eQTN signals, representing 11 787 eQTN loci from 377 genes (Figure S8 and Data S14). The number of eQTNs identified for each expressed lignin biosynthetic genes ranged 1–7611 and 99.20% of the genes with eQTNs were pleiotropic (Table S7). Remarkably, in 90.91% of the associated genes, we detected eQTNs in their corresponding ncRNA genes and 96.97% of eQTN signals in TF genes were associated with their corresponding targets, supporting the regulatory roles of ncRNAs/TFs for lignin biosynthetic genes. The number of eQTNs in noncoding regions was much higher than that in coding regions (17 048 vs. 3510), suggesting that noncoding sequences might play dominant roles in the regulation of expression of lignin biosynthetic genes. The distribution of eQTN signals for associated genes was compared. We identified 162 *cis*‐eQTNs and 20 396 *trans*‐eQTNs, indicating that *trans*‐eQTNs were more frequent across the whole genome.

We identified 3262 eQTNs whose localized genes were significant in association studies, including 36 significant SNPs. For example, three eQTN loci of *Ptr‐COMT30* (L57_SNP8, L57_SNP9 and Ptr‐HD‐ZIP54_SNP13) also associated with LC (Figure S9a–d). Variants within L57 and Ptr‐HD‐ZIP54 also regulated the expression of 10 additional genes as *cis*/*trans*‐eQTNs (Data S14), of which the expression levels of three genes (*Ptr‐COMT30*,* Ptr‐HCT12* and *Ptr‐PO54*) were positively correlated with LC (*r *=* *0.187, 0.160 and 0.138, respectively, *P *<* *0.01; Figure S9e), indicating that SNPs in causal genes might affect phenotypes by regulating the expression of other genes. Interestingly, L57_SNP21 affected *Ptr‐PO64* as a *cis*‐eQTN, while it functioned as a *trans*‐eQTN for *Ptr‐CCR29* and *Ptr‐CCR33*, indicating the diverse actions of eQTNs for traits.

Additionally, the phenotypes were determined by expressions of the causal lignin biosynthetic genes, which were affected by multi‐eQTNs. Of the 42 associated expression traits, 13 were significant in association mapping. For example, *Ptr‐LAC27* (Ptr‐LAC27_SNP50) was identified to associate with FW, whose expression level was negatively correlated with FW (*r *=* *−0.353, *P *<* *0.01; Figure S9f). *Ptr‐LAC27* expression was also determined by one *cis*‐eQTNs and 66 *trans*‐eQTNs. Of these, four *trans*‐eQTNs in four causative genes (*Ptr‐LAC8*,* Ptr‐LAC26*,* Ptr‐TCR2* and *L50*) displayed potential epistatic interactions for the expression of *Ptr‐LAC27* (Figure S9g‐i), including two causal loci for FW (Ptr‐LAC8_SNP27 and Ptr‐LAC26_SNP13). Thus, *Ptr‐LAC27* probably affected FW by regulating its own gene expression, and this process might be regulated by *Ptr‐LAC8*,* Ptr‐LAC26*,* Ptr‐TCR2* and *L50*. These findings suggested that causal genes might contribute to phenotypes by affecting the expression of other genes and/or are regulated by multiple eQTNs.

### Functional interpretation of 4CL and C4H underlying growth and wood property traits of P. tomentosa

**Figure 4:**
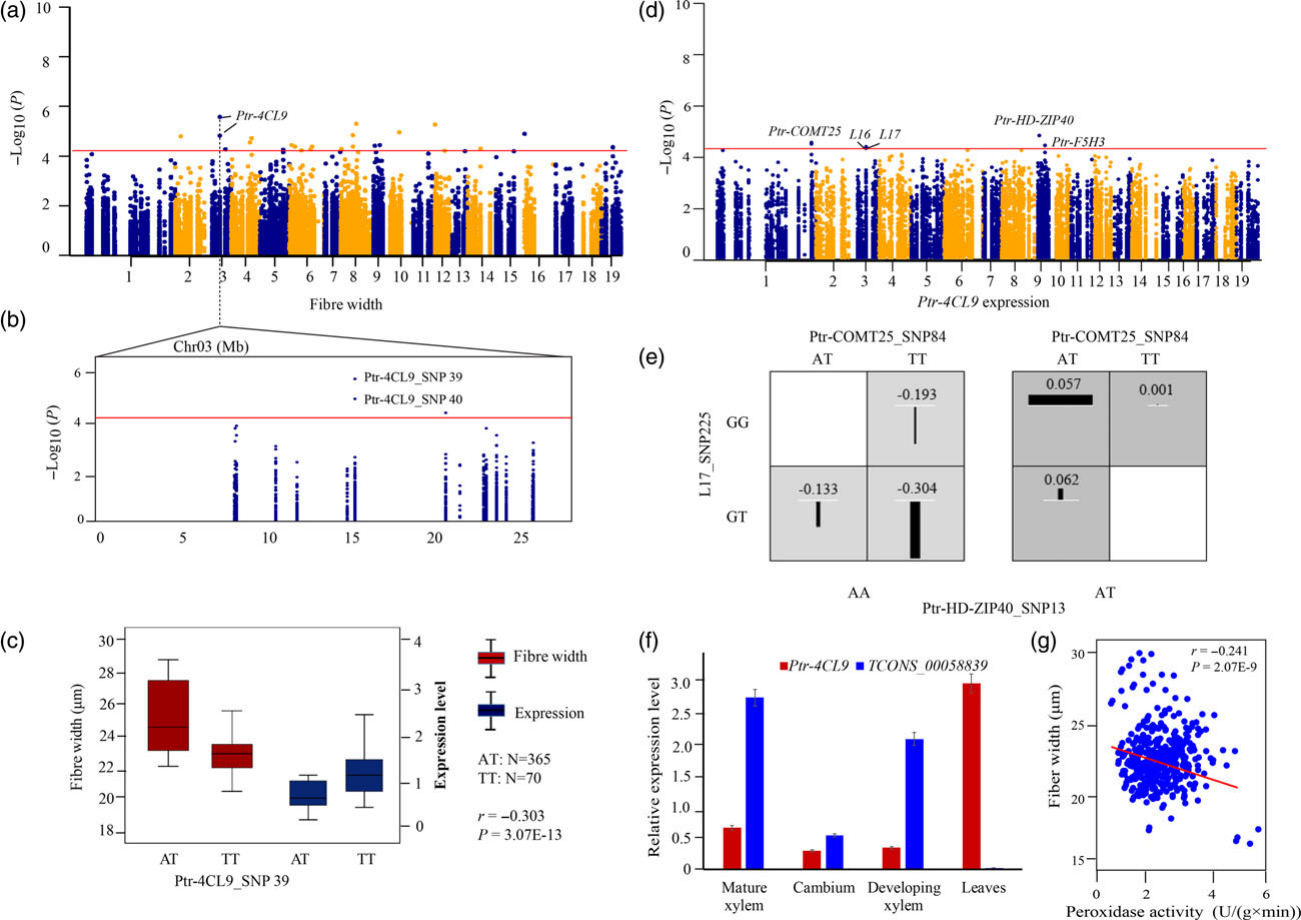
The functional prediction of *Ptr‐4CL9* for fibre width. (a) Manhattan plot displaying the association results for fibre width marked with the lead gene of *Ptr‐4CL9*. (b) Regional association plot for Ptr‐4CL9_SNP39/40 (*4CL9*). (c) The genotype effects of Ptr‐4CL9_SNP39 for fibre width and expression levels of *Ptr‐4CL9*. The ‘*r’* represents the correlation of expression levels and phenotypic variation, which was calculated by Pearson's correlation coefficient. (d) Manhattan plot displaying the association results for *Ptr‐4CL9* expression marked with the significantly associated genes. (e) The epistatic effects of three‐locus genotype combinations for the expression level of *Ptr‐4CL9*. (f) Expression patterns of *Ptr‐4CL9* and its *cis* lncRNAs in four tissues of *P. tomentosa*. (g) Plot of correlation between the peroxidase enzymatic activity and phenotype variations of fibre width.

To further confirm the findings in our association studies, we conducted an in‐depth investigation on how causal SNPs contributed to phenotypes. We identified that Ptr‐4CL9_SNP 39 was a lead SNP for FW (*P *=* *3.01E‐06) (Figure 4a–b), and the expression level of *Ptr‐4CL9* was negatively correlated with FW (*r *=* *−0.303, *P *<* *0.01) (Figure 4c). We detected two strong *cis*‐eQTN signals, found in *Ptr‐4CL9*'s corresponding lncRNA loci (L16 and L17), and four *trans*‐eQTNs that determine the expression of *Ptr‐4CL9* (Figure 4d). Of these, two *trans*‐eQTNs (Ptr‐HD‐ZIP40_SNP13 and Ptr‐COMT25_SNP84) and one *cis*‐eQTN (L17_SNP225) showed clear epistatic interactions, and different genotype combinations of these SNPs contributed differently to the expression of *Ptr‐4CL9* (Figure 4e). Tissue‐specific analysis showed a clear expression correlation between *Ptr‐4CL9* and its *cis*‐lncRNA *TCONS_00058839* (*r *=* *−0.611, *P *<* *0.01; Figure 4f). Notably, association mapping underlying PO enzymatic activities showed that Ptr‐4CL9_SNP34 was significantly associated with PO activity (*P *=* *4.00E‐03, *Q *<* *0.1, *R* ^2^ = 12.15%). The significant correlation (*r *=* *−0.241, *P *<* *0.01) was observed between PO activity and FW (Figure 4g), implying that *Ptr‐4CL9* might affect FW by regulating PO activity.

**Figure 5:**
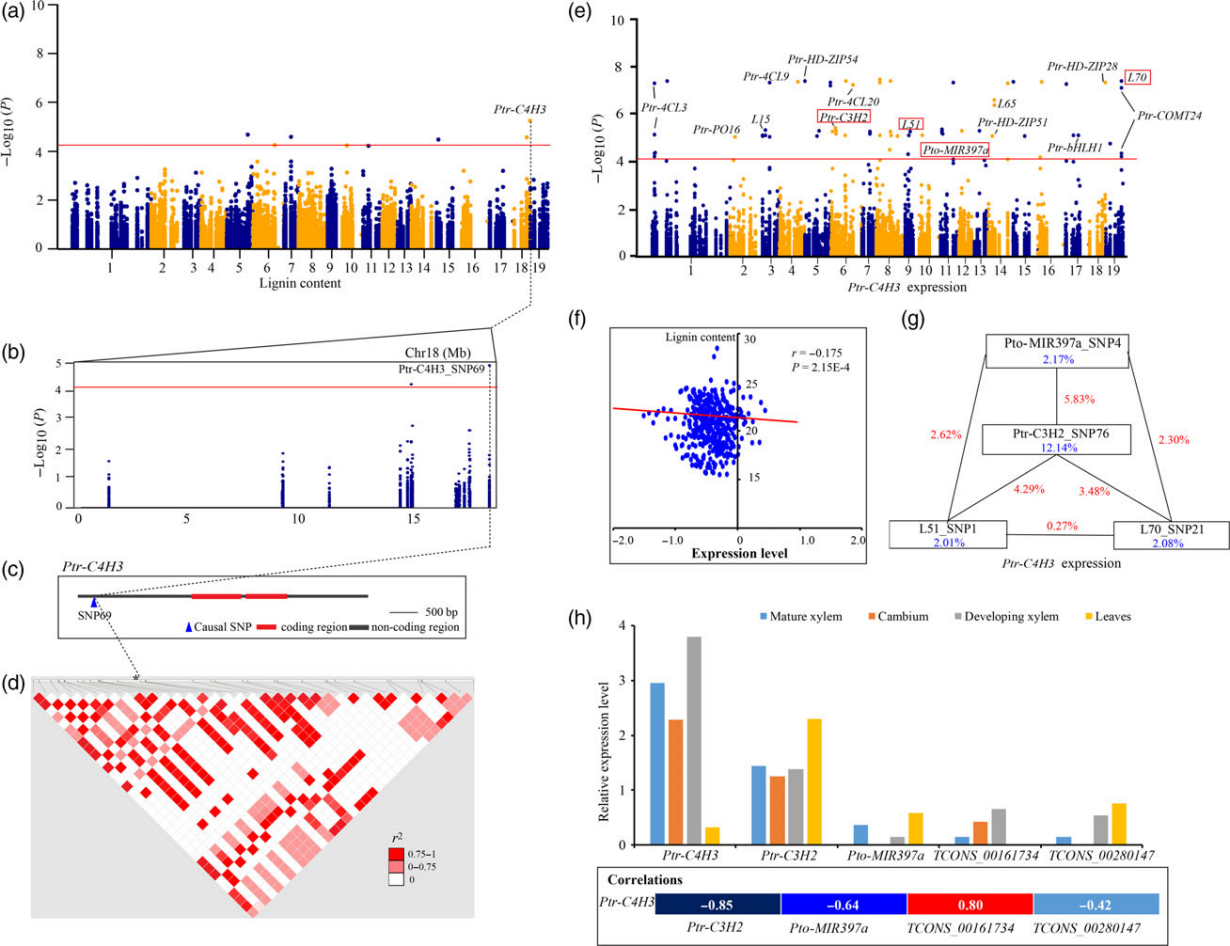
The proposed function of *Ptr‐C4H3* for lignin content. (a) Manhattan plot displaying the association results for lignin content marked with the lead gene of Ptr‐*C4H3*. (b) Regional association plot for Ptr‐C4H3_SNP69. (c) The gene structure of *Ptr‐C4H3*. (d) Pairwise LD between the lead SNP (Ptr‐C4H3_SNP69) and SNPs around it. (e) Manhattan plot for the association results of *Ptr‐C4H3* expression marked with significantly associated genes; the red frame represents the four causal genes with epistatic interactions. (f) Correlation for lignin content and normalized expression levels of *Ptr‐C4H3*, which is indicated by ‘*r’* calculated by Pearson's correlation coefficient. (g) The epistatic interactions of four loci for variations in lignin content. The blue values indicate the single variant effects and the red values represent the pairwise effects. (h) Expression patterns (top) and the Pearson's correlation coefficient (bottom) of *Ptr‐4CL9* and its eQTNs hosted genes in four tissues and organs of *P. tomentosa*.

Ptr‐C4H3_SNP 69 was the lead SNP for LC (*P *=* *1.29E‐05) (Figure 5a–d), and 15 independent eQTNs were identified in causative genes to determine the expression of *Ptr‐C4H3* (Figure 5e), which was negatively correlated with the phenotypic variation of LC (*r *=* *−0.175, *P *<* *0.01) (Figure 5f). Epistasis analysis showed that four *trans*‐eQTNs formed interaction networks for *Ptr‐C4H3* expression (Figure 5g), implying that *Ptr‐C4H3* might be regulated by the four causal genes, *Pto‐MIR397a*,* L51*,* L70* and *Ptr‐C3H2*. Tissue expression analysis also showed significant correlations of *Ptr‐C4H3* with these four genes (Figure 5h), supporting the hypothesis that these causal genes might indirectly affect LC. Additionally, association analysis with enzymatic activity revealed that Ptr‐C4H3_SNP104 associated with CAD activity (*P *=* *3.82E‐03, *Q *<* *0.1, *R* ^2^ = 8.11%), and very weak negative correlations (*r *=* *0.11, *P *=* *1.20E‐02) were shown between lignin content and CAD enzymatic activity, indicating a potential alternative pathway for *Ptr‐C4H3* affecting LC.

## Discussion

### Genetic basis for the coordinated network underlying the lignin biosynthesis pathway for tree growth and wood properties in *P. tomentosa*

The phenylpropanoid biosynthesis pathway is the principal determinant of lignin content (Bonawitz and Chapple, 2010; Ros, 1997). Previously reported lignin biosynthetic genes are regulated by several categories of genetic factors (miRNAs, lncRNAs and TFs), resulting in phenotype diversification (Lu *et al*., 2013; Patzlaff *et al*., 2003; Zhou *et al*., 2017). Thus, our primary goal was to comprehensively understand the interplay of these genetic factors in lignin biosynthesis pathway, and dissect their roles in tree growth and wood properties.

Combining bioinformatics prediction, degradome sequencing and expression pattern analysis, we identified 190 lncRNAs, 36 miRNAs and 81 TFs that were associated with 203 lignin biosynthetic genes of *P. tomentosa*, indicating the universal regulation of lignin biosynthesis by these factors. The majority of the upstream regulators exhibited strong expression correlations to their putative target genes (Figure S2), suggesting that these regulators could affect lignin biosynthesis pathway by regulating the expression of lignin biosynthetic genes (Shi *et al*., 2010), and offering a new understanding of these genetic factors in tree growth and wood formation.

Association mapping is a popular strategy for determining the causal genes in population genetics studies of trees (Ingvarsson and Street, 2011; Neale and Savolainen, 2004). Here, we detected 124 SNP–trait associations harbouring different additive/dominant effects for phenotypes (Figure S5), and a majority of the loci (52.42%) were in genes encoding ncRNAs and TFs, suggesting that tree growth and wood formation are regulated by these genetic factors (Solovieff *et al*., 2013). We demonstrated that 12 pleiotropic genetic factors contributed to wood formation with diverse genetic effects, suggesting that these genes affect wood formation in different ways. The coordinated regulation of wood formation by ncRNAs and TFs largely depended on the different genetic effects of the loci. For instance, eight independent SNPs from three types of genetic factors conferred the fixed genotype combinations for LC (Figure 1), which demonstrated that the phenotypic variations were the result of the interplay of many genetic factors, and the genes and genetic effects would modify across the traits (Bac‐Molenaar *et al*., 2015; Du *et al*., 2015). These findings offer additional resources for gene‐based breeding of trees and provide valuable clues for understanding the coordinated networks of miRNAs, lncRNAs and TFs in the lignin biosynthesis pathway in *Populus*.

Linkage disequilibrium has significant implication on SNP–trait associations. The decay of LD was substantial rapidly than previously reported in *P. trichocarpa* (Slavov *et al*., 2012). Factors for this result could explain below. First, the population structure and the individuals of the association population could cause the differences of LD decay in *Populus* (Terwilliger and Hiekkalinna, 2006). Second, the MAF thresholds used for SNP screening and allele frequency of the loci were probably the considerable reasons for LD detection (Slavov *et al*., 2012; VanLiere and Rosenberg, 2008). Finally, LD in our studies were estimated based on the candidate genes, which was different from the previously studies on genome resequencing data (Slavov *et al*., 2012). Comparably, the LD decay in our study was more consistent with that on candidate gene‐based association mapping (Gilchrist *et al*., 2006; Wegrzyn *et al*., 2010).

### Epistasis analysis and eQTN mapping interpreted the multifactor genetic networks in the lignin biosynthesis pathway underlying growth and wood properties in Populus

Epistasis is a critical component for the genetic basis of quantitative traits, which defines the nonadditive interactions between variants or genes (Mackay, 2014). Here, we identified 745 significant epistatic pairwise interactions (Figure 3a), and a fraction of loci (11 SNPs) were detectable with additive/dominant effects, suggesting that epistasis analysis could capture loci with minor effects (Xu and Jia, 2007) and providing information other than additive/dominant effects of significant loci for phenotypes. Interestingly, 68.29% of loci and 50 SNP–SNP pairs associated with multiple traits with various effects, supporting that the pleiotropism of these genes affects wood formation of *P. tomentosa* (Figure S7).

We enriched the lignin biosynthesis pathway using genes encoding lncRNAs, miRNAs and TFs that had epistatic interactions with lignin biosynthetic genes (Figure 2). TFs and ncRNAs also affected phenotypic variations by linking two lignin biosynthetic genes (Figure 3b–c), which supported the crosstalk among the four genetic factors and proposed an alternative pathway for the regulatory roles of TFs and ncRNAs. Additionally, the interaction graph for α‐cellulose content sufficiently illustrated that epistasis had stronger effects than single loci (Figure 3d–e), which supported the notion that allelic variations and epistatic interplays are dominant drivers of phenotypes (Mackay, 2014). These findings demonstrated that the genetic factors underlying the lignin biosynthesis pathway are network dependent (Zhao and Dixon, 2011). TFs and ncRNAs are essential components in the lignin biosynthesis pathway, and our understanding of the heritability of growth and wood properties would be lacking if we failed to account for their effects.

Much work is being devoted to exploring the causal loci for phenotypes at the transcriptional level. Investigation of eQTNs provides insights into the gene expression effects of candidate loci and helps to unravel the relationship between genotypes and phenotypes (Westra and Franke, 2014). In our studies, the eQTNs identified for 42 expressed lignin biosynthetic genes were more abundant in noncoding regions than those in coding sequences (82.93% vs. 17.07%) (Figure S8), highlighting the regulatory roles of noncoding sequences for quantitative traits (Li *et al*., 2012a, b; Liu *et al*., 2017). Additionally, *trans*‐eQTNs are more frequent in the genome, which indicates that genes are largely regulated by *trans*‐eQTNs (Drost *et al*., 2010). These findings were consistent with studies in *P. trichocarpa* and humans, demonstrating the importance of long‐range control of gene expression and the dominant roles of noncoding sequences for regulation (Bryois *et al*., 2014; Drost *et al*., 2010).

Here, eQTN analysis demonstrated that causal genes could affect phenotypes through an alternative pathway involving miRNA, lncRNA and TF genes at the transcriptional level. Collectively, the causal SNPs and/or genes affect tree growth and wood properties in two ways. Causal genes (*L57* and Ptr‐HD‐ZIP54) might directly contribute to the traits according to the association results at genomic level, and alternatively, they might also affect lignin content by regulating the expression of additional genes by *cis*/*trans*‐eQTNs within them (Figure S9a–d). This notion was supported by the significant correlations between phenotype variations and gene expression (Figure S9e), and the functions of these causal genes should be investigated in the future. Additionally, a causal gene could affect phenotypes by regulating its own expression, and several genes might contribute to expression dynamics in this process. These observations were supported by the distinct contributions of epistatic interactions of four *trans*‐eQTNs for *Ptr‐LAC27* (Figure S9f–i). In particular, variants in ncRNA and TF genes played a significant role in this process, illustrating the additional regulation model that ncRNAs and TFs regulate gene expression at genomewide levels. Collectively, the detection of eQTNs demonstrated another layer of interaction of the four genetic factors (miRNAs, lncRNAs, TFs and lignin biosynthetic genes) for phenotypes, which links the genetic variation with phenotype diversification, and emphasizes that ncRNAs and TFs participate in tree growth and wood formation at the genomewide level.

Previously, substantial regulatory networks have been reported for wood formation in trees (Du *et al*., 2015; Zhou *et al*., 2017), and they dissected a well‐characterized pathway involves in a fraction of major genes, such as miR397a (Lu *et al*., 2013) and the MYB gene family (Patzlaff *et al*., 2003). Here, we dissected the multigene genetic network underlying lignin biosynthesis pathway includes lncRNAs, miRNAs and TFs, which provides a more comprehensive genetic networks for wood formation and captures the major loci in noncoding transcripts. Notably, epistasis explored more loci with minor effects for the networks, which is reasonable for application in marker‐assisted breeding. Importantly, our study also demonstrated the alternative function of causal genes within this multifactor network at transcriptional level, providing important leads to functional studies on understanding the mechanisms whereby natural variants leads to complex traits in trees.

### Functional interpretation of causal genes associated with growth and wood property traits of P. tomentosa

Based on our findings, two lead SNPs contributed to phenotypes via different methods, and the integrated strategy using association mapping to find the underlying additive, dominant and epistatic effects, and eQTN analysis helped to define the putative mechanisms of causal SNPs for quantitative traits in *P. tomentosa*. Additionally, analysis of enzymatic activities in the lignin biosynthesis pathway also aided in the interpretation of the functional roles of causal genes (Bonawitz and Chapple, 2010).

We detected that a lead SNP, Ptr‐4CL9_SNP39, was associated with FW (Figure 4a–c), indicating the potential roles of *4CL9* on FW. We propose the following mechanisms for this association. First, *Ptr‐4CL9* might affect FW through regulating its own expression by *Ptr‐HD‐ZIP40*,* Ptr‐COMT25* and *L17* (Figure 4d). Allelic interactions of three eQTNs in three regulators might play critical roles in expression variations (Figure 4e), including the *cis*‐lncRNA of *TCONS_00058839*, whose expression showed significant correlations in the tested tissues (Figure 4f). As an alternative pathway, *Ptr‐4CL9* might affect FW by regulating PO enzymatic activity, which was supported by significant associations of Ptr‐4CL9_SNP34 with PO activity. In *P. deltoides*, PO activity and FW are somewhat related under salt stress (Li *et al*., 2003), which supported the negative correlations between the expression of *4CL9* and PO activity (Figure 4g). Further studies are needed to determine this mechanism in trees.

Another example illustrates the possible mechanisms of *Ptr‐C4H3* for LC. Previous studies reported that down‐regulation of *C4H* expression led to decreased levels of lignin content (Sewalt *et al*., 1997). Alternatively, we identified four causal genes (*Pto‐MIR397a*,* Ptr‐C3H2*,* L51* and *L70*) that affect the expression of *Ptr‐C4H3* (Figure 5e–g). Of which, *Pto‐MIR397a* has been characterized as a master regulator of LC by down‐regulating the expression of *LAC* in *Populus* (Chen *et al*., 2015; Lu *et al*., 2013). Notably, *Pto‐MIR397a*,* Ptr‐C3H2*, and two lncRNAs in *L51* (*TCONS_00161734*) and *L70* (*TCONS_00280147*) also showed significant correlations in tissue expression analysis, supporting that epistatic interactions of genotypes within the four loci contributed to the expression of *Ptr‐C4H3* (Figure 5h). Association studies of enzymatic activity showed that *C4H‐3* (Ptr‐C4H3_SNP104) significantly associated with CAD activity, but a very weak correlation was observed between lignin content and CAD activity. The change of CAD activity did not significantly alter lignin content, while the lignin structure and composition were altered in tobacco stems (Halpin *et al*., 1994). These findings indicated that *C4H‐3* alters lignin composition and structure by affecting the enzymatic activity of CAD. Further studies are needed to investigate the detailed mechanisms.

In summary, we identified a functional link between genetic variants underlying the lignin biosynthesis pathway and tree growth and wood formation. The integration of multiple strategies, including association mapping (additive, dominant and epistasis), gene expression profiling and analysis of enzymatic activities, has facilitated the identification of candidate genetic factors (Bonawitz and Chapple, 2010; Deng *et al*., 2017; Wen *et al*., 2014). Epistasis is a critical component of genetic effect for the phenotypes and is important for our understanding of heritability (Xu and Jia, 2007). The eQTNs allow for resolution down to the single‐nucleotide level by determining the function of alleles in phenotypes (Liu *et al*., 2017). LncRNAs, miRNAs and TFs have an important and broad‐spectrum impact on the lignin biosynthesis pathway, which affects the genetic architecture of tree growth and wood formation. Also, the endogenous target mimicry also provides a crucial regulatory mechanism of miRNA‐lncRNA‐mRNA in plants, which should be considered in the future (Franco‐Zorrilla *et al*., 2007; Karakülah *et al*., 2016). Importantly, more major regulators (lncRNAs, miRNAs and TFs) will be found if we used the integrated mapping strategy (genomewide association studies and eQTN mapping), which have the substantial potential to accelerate the genetic improvement in perennial trees. Also, natural variants within these regulators could be effectively used as these regulators exist in the upper layer of regulation so that it might have the master regulation roles for the downstream genes, which would decrease the cumbersome of genetic manipulation in trees. Future studies should focus on multiple aspects, such as enzymology, metabolomics and phenomics, which could improve precision and efficiency in uncovering the genetic basis of complex traits, and benefit tree breeding and improvement.

## Experimental procedures

### Population materials and DNA extraction

The association population was composed of 435 unrelated individuals of *P. tomentosa*, representing almost all the natural distribution of *P. tomentosa, that is* the southern, northwestern and northeastern region of China. This collection was selected from a clonal arboretum that includes 1047 individuals of *P. tomentosa* assembled from an area of 1 million km^2^ along the Yellow River (30–40°N, 105–125°E), which was established in Guan Xian Country (Shandong Province, China, 36°23′N, 115°47′E) in 1982, using a randomized complete block design approach with three clonal replications (Du *et al*., 2012). Fresh leaves were harvested from each genotype in the association population, and total genomic DNA was extracted using the DNeasy Plant Mini Kit (Qiagen, Shanghai, China) according to the manufacturer's protocol.

### Phenotypic data

Ten tree growth and wood property traits were scored for all individuals in the association population with at least three replications per genotype. The growth traits were as follows: DBH (cm), H (m) and V (m^3^). The wood properties were as follows: FW (μm), FL (mm), MFA (degrees), LC (%), HC (%), CC (%) and HEC (%). The detailed sampling and measurement methods were reported previously (Du *et al*., 2014). The phenotypic variance (ANOVA) and Pearson's correlations coefficients (*r*) for the 10 quantitative traits were calculated by SPSS Statistics v19.0 (SPSS Inc., Chicago, IL), in which abundant phenotypic variations were observed and 72.22% of pairwise correlations were significant at *P *<* *0.05 (Table S8–S9). Additionally, two enzymatic activity traits, PO (U/(g × min)) and CAD (U/(g × min)), from the mature xylem of *P. tomentosa* in the association population were measured according to the protocol of a plant PO assay kit and a plant CAD assay kit (Nanjing Jiancheng Bioengineering Institute, Jiangsu Province, China), respectively.

### RNA isolation and RNA‐sequencing

Total RNAs were extracted from the cambium, developing xylem, mature xylem and leaves of 1‐year‐old *P. tomentosa* clone ‘LM50’ planted in Guan Xian Country, using the Plant Qiagen RNAeasy kit following the manufacturer's instructions, which were used for transcriptome sequencing of mRNAs and lncRNAs. For biological replicates, three individuals were used in our studies. FPKM (fragments per kilobase of transcript per million fragments) were used to normalize the transcripts’ expression. Transcripts abundance failed to detect in any tested tissues were excluded in our analysis. The processing of transcriptome data is described in Methods S1.

### RT‐qPCR

The RT‐qPCR was performed on a 7500 Fast Real‐time PCR system using SYBR Premix Ex Taq (TaKaRa, Dalian, China). The cDNA templates were transcribed from total RNA extracted from the cambium, developing xylem, mature xylem and leaves of 1‐year‐old *P. tomentosa* clone ‘LM50’. The gene‐specific primers were designed by Primer Express v5.0 software (Applied Biosystems; Data S15). All reactions were performed with triplicate technical and triplicate biological repetitions with *Actin* (EF145577) as the internal control, according to the PCR program described in Zhang *et al*. (2011). The melting curve was used to examine the specificity of the amplification, and Opticon Monitor Analysis software v3.1 was used to analyse the data.

### Identification of lignin biosynthetic genes in *P. tomentosa*

Thirteen lignin biosynthetic gene families (298 gene models) were determined by phenylpropanoid biosynthesis pathway in the KEGG database (http://www.kegg.jp/kegg/) and the reports by Shi *et al*. (2010), based on *P. trichocarpa* genome annotation v3.0 (Tuskan *et al*., 2006), and 203 of them were expressed in at least one tested tissue of *P. tomentosa* (Data S1). The numerical IDs for the genes were in accordance with Shi *et al*. (2010). The newly identified genes, which were not reported in Shi *et al*. (2010) but were annotated in the KEGG database and the *P. trichocarpa* genome v3.0, were numbered consecutively.

### Prediction of miRNAs and lncRNAs for lignin biosynthetic genes

Small RNAs from six tissues (leaf, phloem, cambium, developing xylem, mature xylem and shoot apex), collected from 1‐year‐old *P. tomentosa* clone ‘LM50’, were used to detect the transcript abundance of miRNAs according to the method described by Xie *et al*. (2016), and three individuals were used as the biological replicates. TPM (transcripts per million) was used to normalized the expression of miRNA transcripts. The lncRNA data sets identified in leaf, cambium, developing xylem and mature xylem have been reported previously (Tian *et al*., 2016; Zhou *et al*., 2017). The methods for predicting miRNAs and *cis*/*trans*‐lncRNAs for lignin biosynthetic genes are described in Methods S1.

### Determination of cis‐regulatory elements and TFs

We obtained the 2000‐bp upstream sequences from the 5′ end of the 203 lignin biosynthetic genes in the *Populus* annotation. The PlantPAN v2.0 (http://PlantPAN2.itps.ncku.edu.tw) was used to analyse the combinatorial *cis*‐regulatory elements and detect the corresponding TFs, using the promoter sequences of the *Populus* genes as background (Chow *et al*., 2016). The significantly over‐represented TFBS were identified with a cut‐off of *P *<* *0.05 and represented in at least 90% of the genes examined, information that was used for determining the regulatory TFs with each gene.

### Genotyping by resequencing

The 435 unrelated individuals of *P. tomentosa* were resequenced using the Illumina GA II platform with a depth of > 15 × (raw data). The filtered reads were mapped to the *Populus* reference genome v3.0 (Tuskan *et al*., 2006), which were used for SNP calling (Methods S1). We obtained genotype data within the full‐length sequences of protein‐coding genes and lncRNA genes, including promoter regions (2000‐bp upstream) and flanking regions (500‐bp downstream), using VCF tools (Danecek *et al*., 2011). BLASTN and BLASTX were used with a cut‐off *E*‐value <1E‐10 to obtain the location of lncRNA genes and miRNA genes. The lncRNA genes clustered in the same genomic location were treated as one lncRNA locus, totalling 71 lncRNA loci (Data S3). The genomic DNA sequences of miRNA genes contained the pre‐miRNA and the 600‐bp flanking regions on each side of the pre‐miRNA. The SNPs used in our analysis are listed in Data S9–S12. The methods for assessing nucleotide diversity and LD are listed in Methods S1.

### SNP‐based association mapping

The mixed linear model (MLM) in TASSEL v5.0 (Bradbury *et al*., 2007) was conducted to test the statistical association between SNPs and tree growth and wood property traits in the association population, accounting for the population structure (Q) and pairwise kinship coefficients (*K*). The K matrix was assessed by SPAGeDi v1.3 (Hardy and Vekemans, 2002), which was reported previously (Du *et al*., 2012), and the Q matrix was evaluated via STRUCTURE v2.3.4 (Evanno *et al*., 2005) based on significant subpopulations (*k* = 3). Additionally, we used GEC software to calculate the effective number of independent SNPs (Li *et al*., 2012a), considering that many of the SNPs should be in LD. The *P*‐value was calculated for each association, and the significance was defined with a suggestive *P*‐value ≤6.89E‐05 (*P *=* *1/*n*;* n* represents the independent marker number, which is roughly a Bonferroni correction).

### Multi‐SNP‐based epistatic interaction analysis

The EPISNP1 package in epiSNP software (Ma *et al*., 2008) was used to test the pairwise epistatic effect with *P*‐value < 1E‐04. Only the SNPs showing significance with *P*‐value <0.01 in SNP‐based association mapping were used for epistasis analysis. The two‐locus interaction effect was divided into four components: AA, AD, DA and DD interactions. A multifactor dimensionality reduction (MDR) algorithm was conducted to investigate the genotype combination effects in our studies (Hahn *et al*., 2003).

### eQTN mapping

The eQTNs, defined as associations between candidate SNPs and the expression level of genes, were performed using the same method as the SNP‐based association studies. Total RNAs extracted from the mature xylem of 435 unrelated individuals of *P. tomentosa* were used for RNA‐seq in 2016, using the methods described above. Library construction and sequencing were performed by Beijing Biomarker Technology Cooperation (Beijing, China). Transcripts expressed in more than 80% of the 435 individuals were retained for eQTN analysis (Data S13). The detected eQTNs located within a 10‐Kb window surrounding the TSS of its targets were regarded as *cis*‐eQTNs, and the others were treated as *trans*‐eQTNs.

### Accession numbers

The lncRNA sequencing data have been deposited in NCBI under the accession number of SRP073689 and the Genome Sequence Archive in BIG Data Center, Beijing Institute of Genomics (BIG), Chinese Academy of Sciences (CAS), under accession number of CRA000992 that is publicly accessible at http://bigd.big.ac.cn/gsa/. The miRNA sequencing data, degradome sequencing data and the raw data of genome resequencing have been deposited in the Genome Sequence Archive in BIG Data Center (BIG, CAS, China) under accession number of CRA000983, CRA000989 and CRA000903, respectively.

## Authors’ contributions

D.Z. designed the experiments; M.Q., Q.D., L.X. and W.L. collected and analysed the data; L.X., W.L., J.X., L.W., Y.S. and B.X. performed the experiments; M.Q. and D.Z. wrote the manuscript; D.Z. obtained funding and is responsible for this article. All authors read and approved the manuscript.

## Supporting information


**Figure S1** The distribution of enriched transcription factor binding sites (TFBS) in the promoters of 203 lignin biosynthetic genes.
**Figure S2** Expression profiles of candidate genes related to the lignin biosynthesis pathway. (a) Transcript abundance of 40 lignin biosynthetic genes selected from the 13 gene families revealed by RNA‐seq (left) and RT‐qPCR (right). (b) Plot of correlation between RNA‐seq and RT‐qPCR for 40 lignin biosynthetic genes in four tissues. (c) The expression patterns of six lncRNAs, six miRNAs, and nine TF genes selected from the candidate genetic factors revealed by RT‐qPCR in four tissues of *P. tomentosa*. (d) Plot of correlation for expression correlations between RNA‐seq and RT‐qPCR of ncRNA/TFs and their corresponding genes.
**Figure S3** Pairwise linkage disequilibrium (LD) between SNP markers within the same chromosome and haplotype blocks across 19 chromosomes. The adjacent SNPs in significant LD are coloured red, and high‐LD blocks (*r* ^2^ ≥ 0.75, *P *≤* *1.0E‐03) are shown in black triangles.
**Figure S4** Decay of LD of candidate genes at the chromosome level in the association population of *P. tomentosa*. Nonlinear regressions of *r* ^2^ onto the physical distance are described by separate curves for each chromosome.
**Figure S5** Manhattan (left) and quantile–quantile plots (right) resulting from the SNP‐based association studies for ten tree growth and wood property traits in the association population of *P. tomentosa*. The red line in each Manhattan plot depicts the Bonferroni‐adjusted significance threshold (6.89 × 10^−5^). The *x* and *y* axes show the genomic position and the significance denoted as −log_10_ (*P*), respectively.
**Figure S6** Significant SNPs and genes identified by SNP‐based association studies. (a) diameter at breast height (DBH), (b) tree height (H), and (c) fibre length (FL). Top, association results of tree growth and wood property traits, the significant genes are marked. Middle, the association results on the same chromosome of significant SNPs whose positions are indicated by black dashed lines. Bottom, the structure of genes with significant SNPs. The red line in each Manhattan plot depicts the Bonferroni‐adjusted significance threshold (6.89 × 10^−5^). The *x* and *y* axes show the genomic position and the significance denoted as –log_10_ (*P*), respectively.
**Figure S7** The epistatic interactions of SNP pairs in the natural population of *P. tomentosa*. Epistatic interactions of SNP pairs for fibre width (a–c), diameter at breast height (d–f), and stem volume (g–i) in the association population of *P. tomentosa*. (a–b, d–e, g–h) The single‐locus effects for the traits. (c, f, i) The epistatic effects of genotype combinations for the traits.
**Figure S8** Summary of the distribution of the eQTNs. (a) The distribution of eQTNs in the four types of genetic factors. (b) The detailed distribution of eQTNs in lignin biosynthetic genes and transcription factor genes (c).
**Figure S9** The interpretation of causal SNPs for phenotypes by eQTNs. (a) Manhattan plot displaying the association results for *Ptr‐COMT30* expression and lignin content. (b) The overlapped associated loci are marked with dashed lines. (c–d) The genotype effects of Ptr‐HD‐ZIP54_SNP13 and L57_SNP8 for lignin content and *Ptr‐COMT30* expressions, respectively. We discarded L57_SNP9 as it was in LD with L57_SNP8. (e) Plot of correlations between lignin content and normalized expression levels of *Ptr‐COMT30*,* Ptr‐HCT12*, and *Ptr‐PO54*. The *r* value indicated Pearson's correlation coefficient. (f) Plot of correlations between fibre width and normalized expression levels of *Ptr‐LAC27*. (g) Manhattan plot displaying the association results for *Ptr‐LAC27* expression marking the causal loci with epistatic interactions. (h) The epistatic effects of four loci for the variations in expression of *LAC‐27*. The blue values indicate the single variant effects and the red values represent the pairwise effects. (i) Box plots revealing the epistatic effects of different genotypic combinations for phenotypic variations of *Ptr‐LAC27* expression.
**Methods S1** The detailed experimental procedures were described for some methods.
**Table S1** Pearson's correlation coefficients for each lncRNA‐mRNA pair.
**Table S2** Pearson's correlation coefficients for each miRNA‐mRNA pair.
**Table S3** The enriched transcription factor binding motifs in the promoters of lignin biosynthesis genes.
**Table S4** Details of single nucleotide polymorphisms (SNPs) within all the candidate genes.
**Table S5** Details of significant SNPs within candidate genes associated with growth and wood properties in the association population of *P. tomentosa*.
**Table S6** Detailed information of significant epistatic SNP‐SNP pairs for each trait in the association population of *P. tomentosa*.
**Table S7** Details of eQTNs identified for each gene in the lignin biosynthesis pathway.
**Table S8** Phenotypic variation of ten growth and wood property traits in the association population of *P. tomentosa*.
**Table S9** Phenotypic correlations for tree growth and wood property traits in the association population of *P. tomentosa*.

**Data S1** The expression profiles of lignin biosynthetic genes detected by RNA‐seq in *P. tomentosa*.
**Data S2** The lncRNA‐mRNA pairs identified in our studies.
**Data S3** The expression abundance of lncRNAs detected by RNA‐seq and lncRNA loci used in our analysis.
**Data S4** The miRNA‐mRNA pairs identified in our studies.
**Data S5** The expression abundance of miRNAs detected by RNA‐seq in our analysis.
**Data S6** The TFBS in each promoter of the lignin biosynthesis genes.
**Data S7** Pearson's correlation coefficients for each TF‐mRNA pair.
**Data S8** The expression abundance of TF genes detected by RNA‐seq used in our analysis.
**Data S9** SNPs within lignin biosynthetic genes used for association analysis.
**Data S10** SNPs within transcription factor genes used for association analysis.
**Data S11** SNPs within lncRNA loci used for association analysis.
**Data S12** SNPs within miRNA genes used for association analysis.
**Data S13** The gene expression data used for expression QTN mapping in our analysis.
**Data S14** Detailed information of significant eQTNs for lignin biosynthetic genes in the association population of *P. tomentosa*.
**Data S15** Real‐time quantitative PCR primers used in our studies.
