# Supplementary material for: Genetic architecture underlying the lignin biosynthesis pathway involves noncoding RNAs and transcription factors for growth and wood properties in Populus
Source: Plant Biotechnol J. 2018 Jul 16;17(1):302–15. doi: 10.1111/pbi.12978 (PMC6330548; doi:10.1111/pbi.12978)
Supplement: Supplementary file 1 — Figure S1 The distribution of enriched transcription factor binding sites (TFBS) in the promoters of 203 lignin biosynthetic genes. Figure S2 Expression profiles of candidate genes related to the lignin biosynthesis pathway. (a) Transcript abundance of 40 lignin biosynthetic genes selected from the 13 gene families revealed by RNA‐seq (left) and RT‐qPCR (right). (b) Plot of correlation between RNA‐seq and RT‐qPCR for 40 lignin biosynthetic genes in four tissues. (c) The expression patterns of six lncRNAs, six miRNAs, and nine TF genes selected from the candidate genetic factors revealed by RT‐qPCR in four tissues of P. tomentosa. (d) Plot of correlation for expression correlations between RNA‐seq and RT‐qPCR of ncRNA/TFs and their corresponding genes. Figure S3 Pairwise linkage disequilibrium (LD) between SNP markers within the same chromosome and haplotype blocks across 19 chromosomes. The adjacent SNPs in significant LD are coloured red, and high‐LD blocks (r 2 ≥ 0.75, P ≤ 1.0E‐03) are shown in black triangles. Figure S4 Decay of LD of candidate genes at the chromosome level in the association population of P. tomentosa. Nonlinear regressions of r 2 onto the physical distance are described by separate curves for each chromosome. Figure S5 Manhattan (left) and quantile–quantile plots (right) resulting from the SNP‐based association studies for ten tree growth and wood property traits in the association population of P. tomentosa. The red line in each Manhattan plot depicts the Bonferroni‐adjusted significance threshold (6.89 × 10^−5^). The x and y axes show the genomic position and the significance denoted as −log10 (P), respectively. Figure S6 Significant SNPs and genes identified by SNP‐based association studies. (a) diameter at breast height (DBH), (b) tree height (H), and (c) fibre length (FL). Top, association results of tree growth and wood property traits, the significant genes are marked. Middle, the association results on the same chromosome of significant S [file PBI-17-302-s002.doc]

## Supporting Information

**Figure S1.** The distribution of enriched transcription factor binding sites (TFBS) in the promoters of 203 lignin biosynthetic genes.

**Figure S2.** Expression profiles of candidate genes related to the lignin biosynthesis pathway.

**Figure S3.** Pairwise linkage disequilibrium (LD) between SNP markers within the same chromosome and haplotype blocks across 19 chromosomes.

**Figure S4.** Decay of LD of candidate genes at the chromosome level in the association population of *P. tomentosa*.

**Figure S5.** Manhattan (left) and quantile-quantile plots (right) resulting from the SNP-based association studies for ten tree growth and wood property traits in the association population of *P. tomentosa*.

**Figure S6.** Significant SNPs and genes identified by SNP-based association studies.

**Figure S7.** The epistatic interactions of SNP pairs in the natural population of *P. tomentosa*.

**Figure S8.** Summary of the distribution of the eQTNs.

**Figure S9.** The interpretation of causal SNPs for phenotypes by eQTNs.

**Table S1.** Pearson’s correlation coefficients for each lncRNA-mRNA pair.

**Table S2.** Pearson’s correlation coefficients for each miRNA-mRNA pair.

**Table S3.** The enriched transcription factor binding motifs in the promoters of lignin biosynthesis genes.

**Table S4.** Details of single nucleotide polymorphisms (SNPs) within all the candidate genes.

**Table S5.** Details of significant SNPs within candidate genes associated with growth and wood properties in the association population of *P. tomentosa*.

**Table S6.** Detailed information of significant epistatic SNP-SNP pairs for each trait in the association population of *P. tomentosa*.

**Table S7.** Details of eQTNs identified for each gene in the lignin biosynthesis pathway.

**Table S8.** Phenotypic variation of ten growth and wood property traits in the association population of *P. tomentosa*.

**Table S9.** Phenotypic correlations for tree growth and wood property traits in the association population of *P. tomentosa*.

**Data S1-S15.** were provided in other formats (Excel), which were submitted as a separate files.

**Data S1.** The expression profiles of lignin biosynthetic genes detected by RNA-seq in *P. tomentosa*.

**Data S2.** The lncRNA-mRNA pairs identified in our studies.

**Data S3.** The expression abundance of lncRNAs detected by RNA-seq and lncRNA loci used in our analysis.

**Data S4.** The miRNA-mRNA pairs identified in our studies.

**Data S5.** The expression abundance of miRNAs detected by RNA-seq in our analysis.

**Data S6.** The TFBS in each promoter of the lignin biosynthesis genes.

**Data S7.** The TFBS in each promoter of the lignin biosynthesis genes.

**Data S8.** The expression abundance of TF genes detected by RNA-seq used in our analysis.

**Data S9.** SNPs within lignin biosynthetic genes used for association analysis.

**Data S10.** SNPs within transcription factor genes used for association analysis.

**Data S11.** SNPs within lncRNA loci used for association analysis.

**Data S12.** SNPs within miRNA genes used for association analysis.

**Data S13.** The gene expression data used for expression QTN mapping in our analysis.

**Data S14.** Detailed information of significant eQTNs for lignin biosynthetic genes in the association population of *P. tomentosa*.

**Data S15.** Real-time quantitative PCR primers used in our studies.

**Methods S1.** The detailed experimental procedures were described for some methods

1. **Transcriptome sequencing and data processing.**

Total RNAs were extracted from the stem cambium, developing xylem, mature xylem, and leaves of 1-year-old *P. tomentosa* clone “LM50” planted in Guan Xian Country, using the Qiagen RNAeasy kit (Qiagen China, Shanghai, China) following the manufacturer’s instructions. For biological replicates, three individuals were used in our studies. Additional on-column DNase digestions were performed three times during RNA purification using the RNase-Free DNase Set (Qiagen). The four strand-specific RNA-seq libraries were constructed after the assessment the quality of RNA samples by NanoDrop ND-1000 and Agilent Bioanalyzer 2100. After quantification by Qubit 2.0 Fluorometer and Agilent 2100 bioanalyzer, the four RNA libraries were sequenced on Illumina Hiseq2500 platform. Library construction and sequencing were performed by Shanghai Biotechnology Cooperation (Shanghai, China).

High-quality reads, filtered by FASTX-Toolkit version 0.0.13 (<http://hannonlab.cshl.edu/fastx_toolkit/index.html>) by removing low quality reads (Q20 < 90%), adaptor sequences, and sequences shorter than 20 nucleotides, were aligned to *Populus* reference genome v3.0 (Tuskan et al., 2006), using Tophat v2.0.9 (Trapnell et al., 2012). Genomic annotations were obtained from Phytozome (http://www.phytozome.net/). Cufflinks v2.1.1 was used to calculate and normalize the expression of assembled transcripts using FPKM (fragments per kilobase of transcript per million fragments) (Trapnell et al., 2012). Transcripts with mapping coverage less than half of the transcript length were removed.

**(2) Determination of *cis*/*trans* lncRNAs and miRNAs for lignin biosynthetic genes in our studies.**

The potential lncRNAs of lignin biosynthetic genes were classified into *cis* and *trans*-acting based on their regulatory effects, which were predicted by two independent algorithms. The prediction of potential *cis*-lncRNAs was based on the physically location to the genes, which the lncRNAs transcribed within a 10kb window upstream or downstream of genes were considered as the *cis*-lncRNAs (Jia et al., 2010). The method searched for the *trans*-lncRNAs was based on sequence complementarity and RNA duplex energy. First, we used BLAST to screen the lncRNAs that were complementary to the genes with cutoff of E-value <1E-05 and identity ≥ 95%. Then, RNAplex was used to calculate the complementary energy between two sequences for the second-round screening with E-value < -60 (Tafer and Hofacker, 2008).

Two methods were used to determine the miRNAs targeted to lignin biosynthetic genes. One is psRNATarget, a small RNA target analysis server (<http://plantgrn.noble.org/psRNATarget>), with expectation ≤ 5.0. Another is determined by degradome sequencing, which was used to identify miRNA cleavage sites. The total RNA samples from the six tissues (leaf, shoot apex, phloem, cambium, developing xylem, and mature xylem) were pooled together with equal amount after RNA purification and integrity confirmation, which were used for degradome libraries construction and sequencing with the Illumina HiSeq2000 according to the methods reported previously (Shamimuzzaman and Vodkin, 2012). Briefly, using T4 RNA ligase (Ambion), a 5′ RNA adapter (5′-GUUCAGAGUUCUACA GUCCGAC-3′) was added to the cleavage products, which possess a free 5′ monophosphate at their 3′ termini. And then, the ligated products were purified and reverse transcribed using an oligo dT primer (5′-CGAGCACAGAATTAATACGACTTTTTTTTTTTTTTTTTTV-3′) by using SuperScript II RT (Invitrogen). The generated cDNA was amplified for six cycles, including 94 ℃ for 30s, 60 ℃ for 20s, and 72 ℃ for 3 min, with primers (forward: 5′-GTTCAGAGTTCTACAGTCCGAC-3′ ; reverse: 5′-CGAGCACAGAATTAATACGACT-3′), and then the PCR products were digested with MmeI and ligated by a double stranded DNA adapter. Finally, the ligation products were amplified, gel-purified, and applied for sequencing-by- synthesis by Illumina Hiseq2000. The CleaveLand pipeline (Addo-Quaye et al., 2009) was conducted to analyze the miRNA cleavage sites based on *P. trichocarpa* genome transcripts v3.0 (CRA000989).

**(3) Re-sequencing data processing and SNP calling**

The 435 unrelated individuals of *P. tomentosa* were re-sequenced using Illumina GA II platform with a depth of >15× (raw data), and the libraries were constructed based on genomic DNA following the manufacturer’s recommendations (Illumina). Paired-end short reads of 100 bp were controlled and clean data were collected by removing low quality reads (< 10% of nucleotides with quality < Q20). Then, the filtered reads were mapped to the *Populus* reference genome using the SOAPaligner (SOAP2 v2.20) with default options (Li et al., 2009). The mapping rate varied of 81% - 92% in different individuals and the effective mapping depth was about 11× for most accessions.

To get high-quality SNPs, the uniquely mapped paired-end reads were used for SNP calling. The genotype likelihood of the genomic position for each tree was calculated by SOAPsnp with default parameters (Li et al., 2009). Low-quality SNPs, whose minor allele frequencies (MAF) ≤ 5% and missing data ≥ 10%, were filtered in further analysis. To evaluate the results of SNP calling, we compared the results with previous SNP data of 10 candidate genes identified by PCR-Sanger sequencing in 120 individuals (Du et al., 2015), which found the accuracy of SNP calling was 97.5%.

**(4) Nucleotide diversity assessment and linkage disequilibrium (LD) tests in the association population of *P. tomentosa***

Based on the genotyping data, the nucleotide diversity parameters, *i.e.* the average number of pair-wise differences per site between sequences (π) (Nei, 1987) and the number of segregating sites (θ_w_) (Watterson, 1975), were calculated using TASSEL v5.0 (Bradbury et al., 2007) for each candidate gene. The squared correlation of allele frequencies (*r^2^*) across each pair of common SNPs was calculated by TASSEL v5.0 with 10^5^ permutations (Bradbury et al., 2007). The decay of LD with physical distances (bp) between the common SNPs were estimated by non-linear regression (Remington et al., 2001). The Haploview v4.2 was used to detect the high-LD haplotype blocks within each chromosome (Barrett et al., 2005).

**References:**

Addo-Quaye C, Miller W and Axtell MJ (2009) CleaveLand: a pipeline for using degradome data to find cleaved small RNA targets. *Bioinformatics* **25**:130-131.

Barrett JC, Fry B, Maller J and Daly MJ (2005) Haploview: analysis and visualization of LD and haplotype maps. *Bioinformatics* **21**:263-265.

Bradbury PJ, Zhang Z, Kroon DE, Casstevens TM, Ramdoss Y and Buckler ES (2007) TASSEL: software for association mapping of complex traits in diverse samples. *Bioinformatics* **23**:2633-2635.

Du Q, Tian J, Yang X, Pan W, Xu B, Li B, Ingvarsson PK and Zhang D (2015) Identification of additive, dominant, and epistatic variation conferred by key genes in cellulose biosynthesis pathway in *Populus tomentosa*. *DNA Research* **22**:53-67.

Jia H, Osak M, Bogu GK, Stanton LW, Johnson R and Lipovich L (2010) Genome-wide computational identification and manual annotation of human long noncoding RNA genes. *RNA* **16**:1478-1487.

Li R, Yu C, Li Y, Lam T, Yiu S, Kristiansen K and Wang J (2009) SOAP2: an improved ultrafast tool for short read alignment. *Bioinformatics* **25**:1966-1967.

Nei M (1987) Molecular evolutionary genetics, Columbia University Press, New York.

Remington DL, Thornsberry JM, Matsuoka Y, Wilson LM, Whitt SR, Doebley J, Kresovich S, Goodman MM and Buckler ET (2001) Structure of linkage disequilibrium and phenotypic associations in the maize genome. *Proceedings of the National Academy of Sciences, USA* **98**:11479-11484.

Shamimuzzaman M and Vodkin L (2012) Identification of soybean seed developmental stage-specific and tissue-specific miRNA targets by degradome sequencing. *BMC GENOMICS* **13**:310.

Tafer H and Hofacker IL (2008) RNAplex: a fast tool for RNA-RNA interaction search. *Bioinformatics* **24**:2657-2663.

Trapnell C, Roberts A, Goff L, Pertea G, Kim D, Kelley DR, Pimentel H, Salzberg SL, Rinn JL and Pachter L (2012) Differential gene and transcript expression analysis of RNA-seq experiments with TopHat and Cufflinks. *Nature Protocols* **7**:562-578.

Tuskan GA, Difazio S, Jansson S, Bohlmann J, Grigoriev I, Hellsten U, Putnam N, Ralph S, Rombauts S, Salamov A, et al (2006) The genome of black cottonwood, *Populus trichocarpa* (Torr. & Gray). *Science* **313**:1596-1604.

Watterson GA (1975) On the number of segregating sites in genetical models without recombination. *Theoretical Population Biology* **7**:256-276.

**
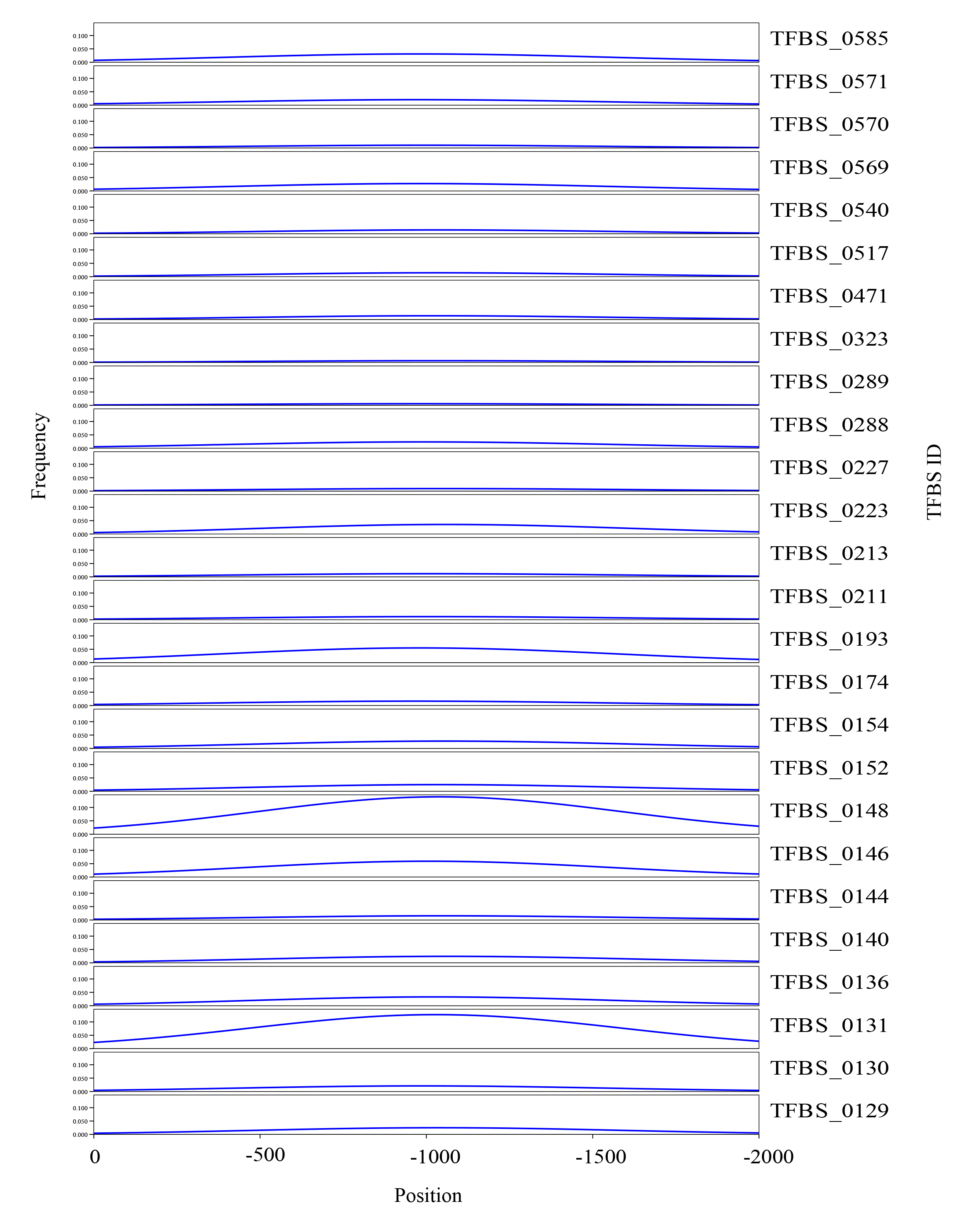
**

**Figure S1. The distribution of enriched transcription factor binding sites (TFBS) in the promoters of 203 lignin biosynthetic genes.**

**
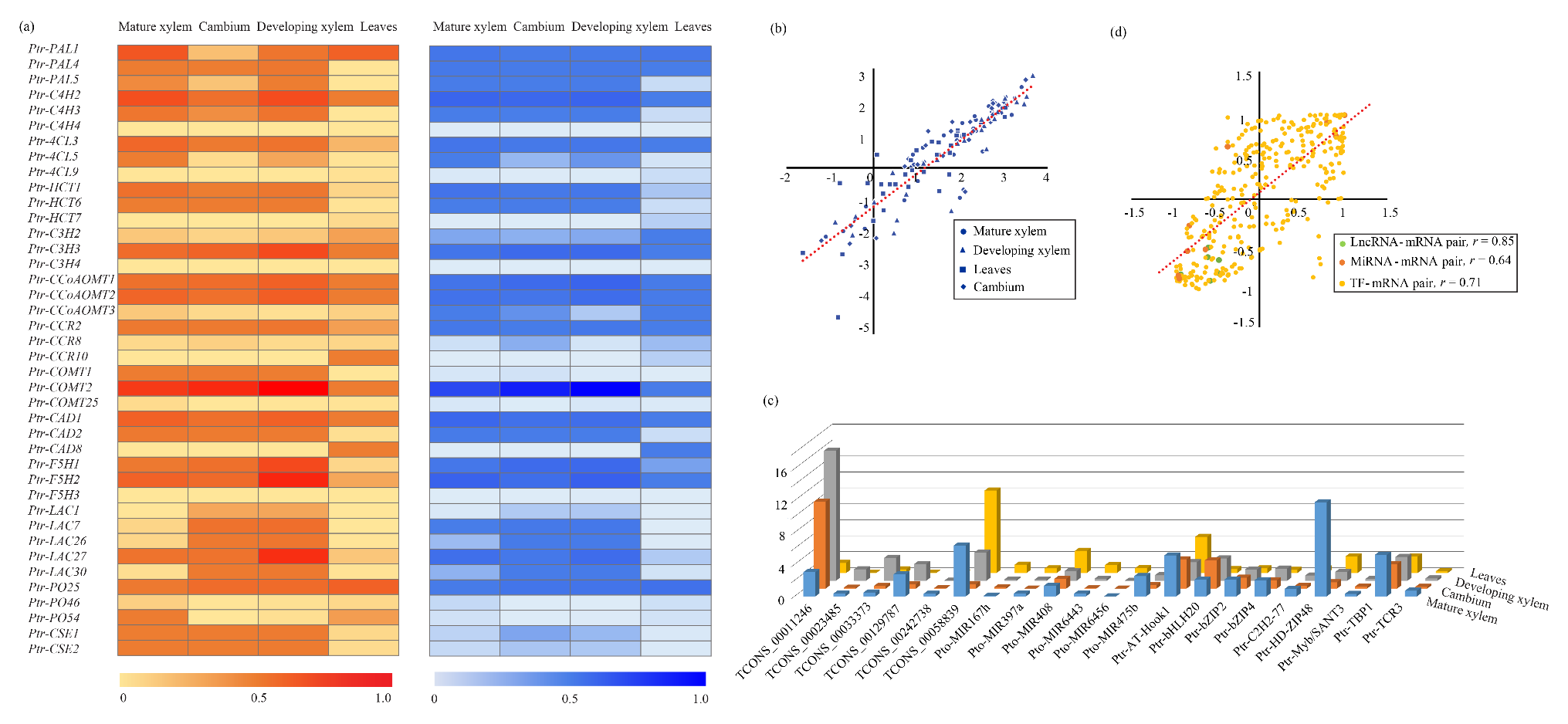
Figure S2. Expression profiles of candidate genes related to the lignin biosynthesis pathway.** (a) Transcript abundance of 40 lignin biosynthetic genes selected from the 13 gene families revealed by RNA-seq (left) and RT-qPCR (right). (b) Plot of correlation between RNA-seq and RT-qPCR for 40 lignin biosynthetic genes in four tissues. (c) The expression patterns of six lncRNAs, six miRNAs, and nine TF genes selected from the candidate genetic factors revealed by RT-qPCR in four tissues of *P. tomentosa*. (d) Plot of correlation for expression correlations between RNA-seq and RT-qPCR of ncRNA/TFs and their corresponding genes.


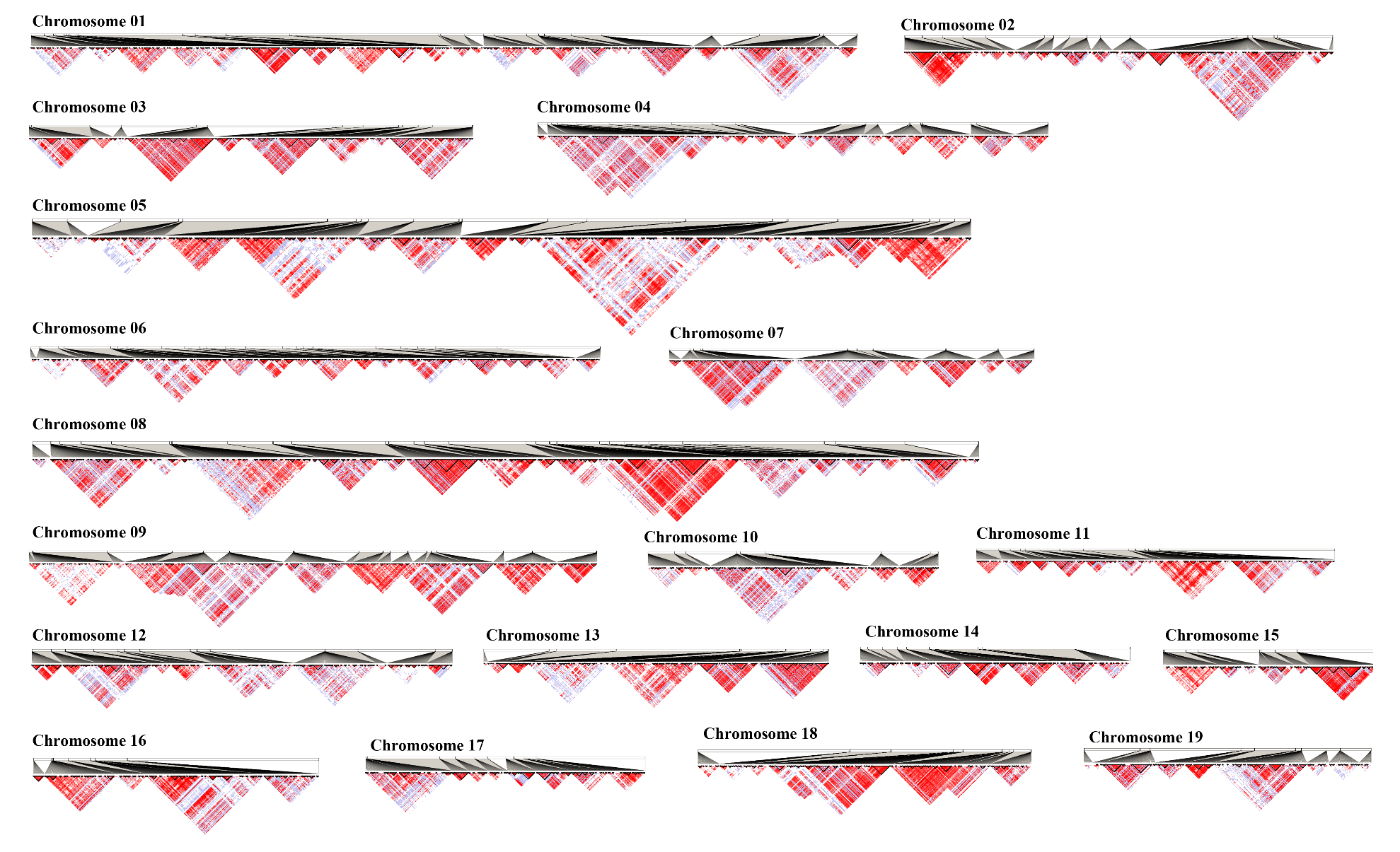
**Figure S3. Pairwise linkage disequilibrium (LD) between SNP markers within the same chromosome and haplotype blocks across 19 chromosomes.** The adjacent SNPs in significant LD are colored red, and high-LD blocks (*r*^2^ ≥ 0.75, *P* ≤ 1.0E-03) are shown in black triangles.


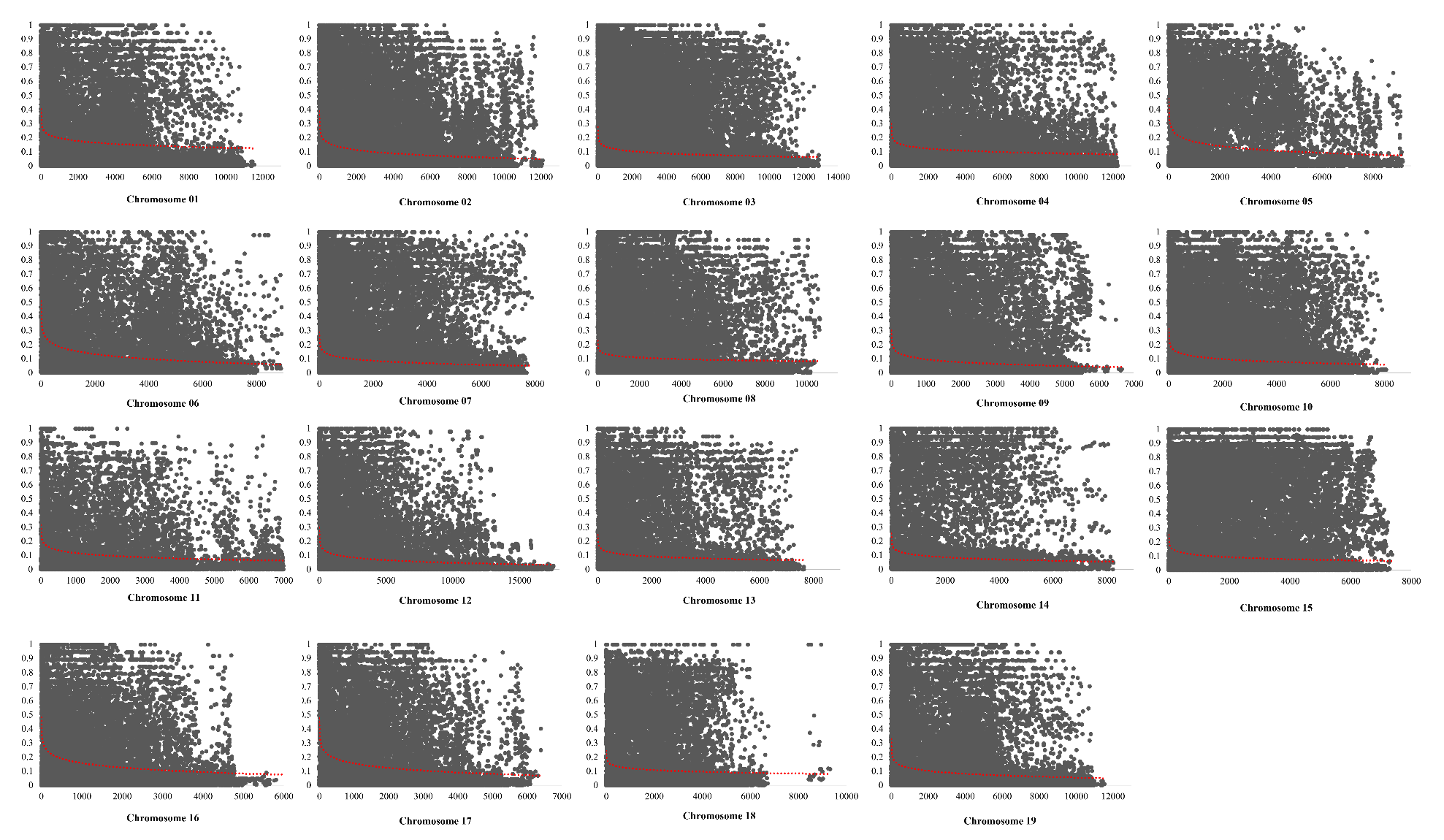


**Figure S4. Decay of LD of candidate genes at the chromosome level in the association population of *P. tomentosa*.** Nonlinear regressions of *r*^2^ onto the physical distance are described by separate curves for each chromosome.

**
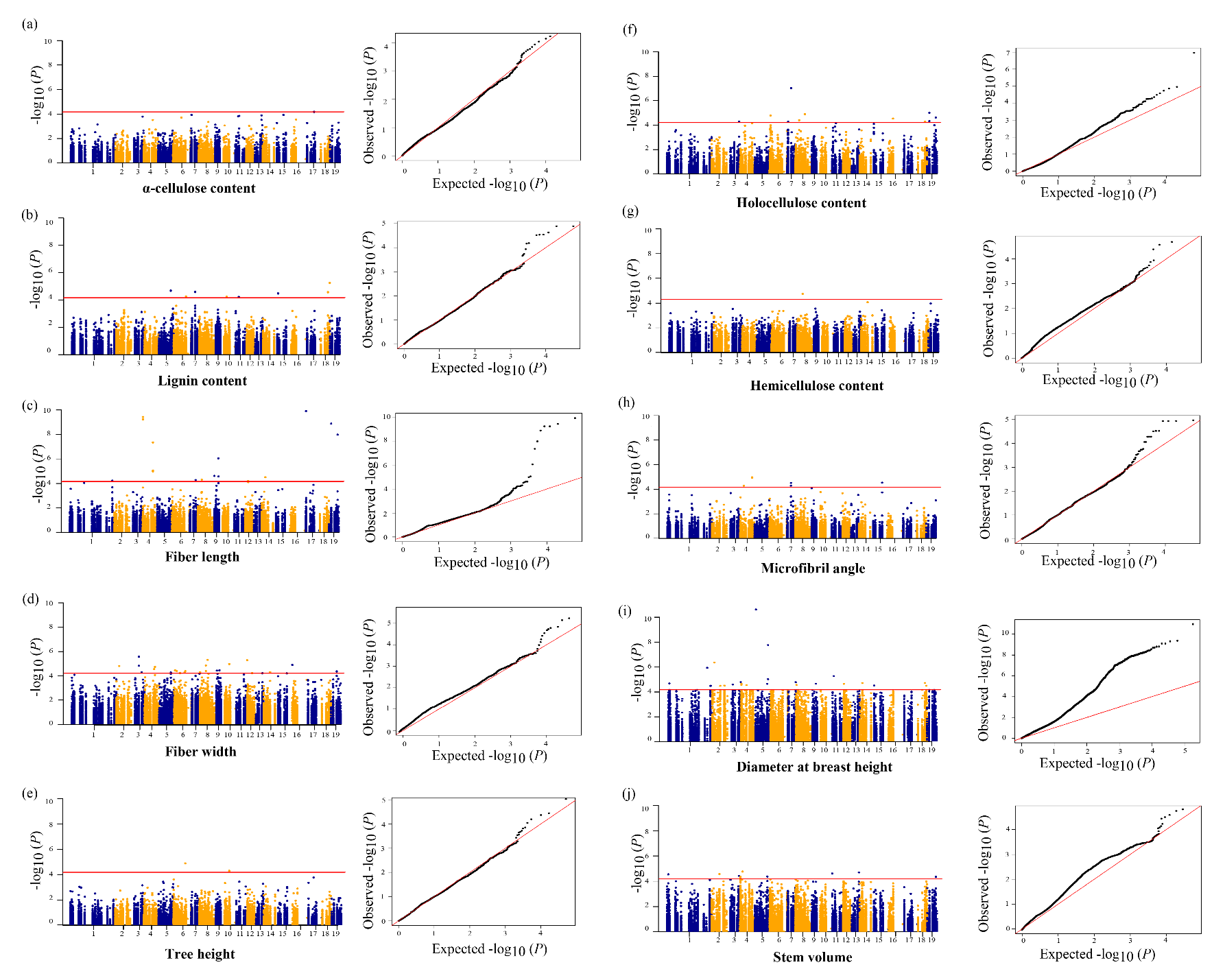
**

**Figure S5. Manhattan (left) and quantile-quantile plots (right) resulting from the SNP-based association studies for ten tree growth and wood property traits in the association population of *P. tomentosa*.** The red line in each Manhattan plot depicts the Bonferroni-adjusted significance threshold (6.89×10^-5^). The *x* and *y* axes show the genomic position and the significance denoted as -log_10_ (*P*), respectively.

**
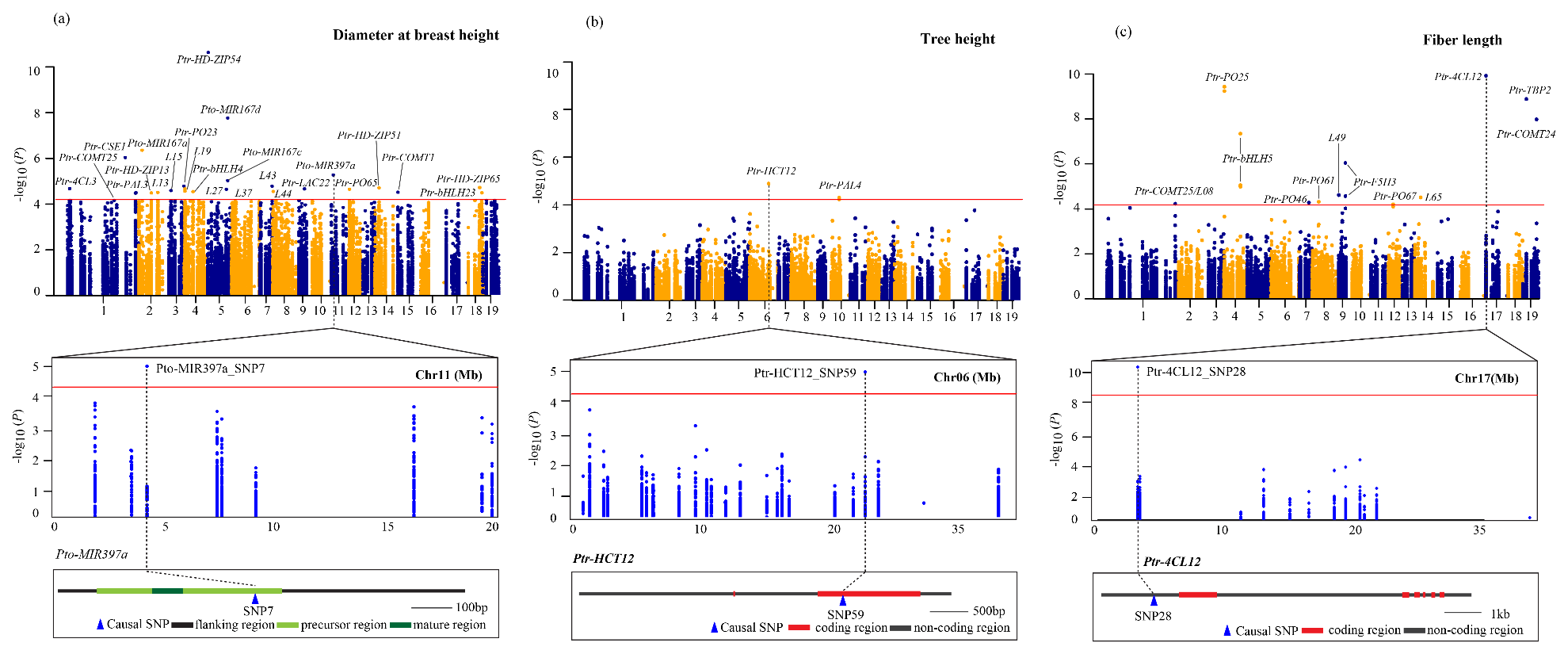
Figure S6. Significant SNPs and genes identified by SNP-based association studies**. (a) diameter at breast height (DBH), (b) tree height (H), and (c) fiber length (FL). Top, association results of tree growth and wood property traits, the significant genes are marked. Middle, the association results on the same chromosome of significant SNPs whose positions are indicated by black dashed lines. Bottom, the structure of genes with significant SNPs. The red line in each Manhattan plot depicts the Bonferroni-adjusted significance threshold (6.89×10^-5^). The *x* and *y* axes show the genomic position and the significance denoted as –log_10_ (*P*), respectively.


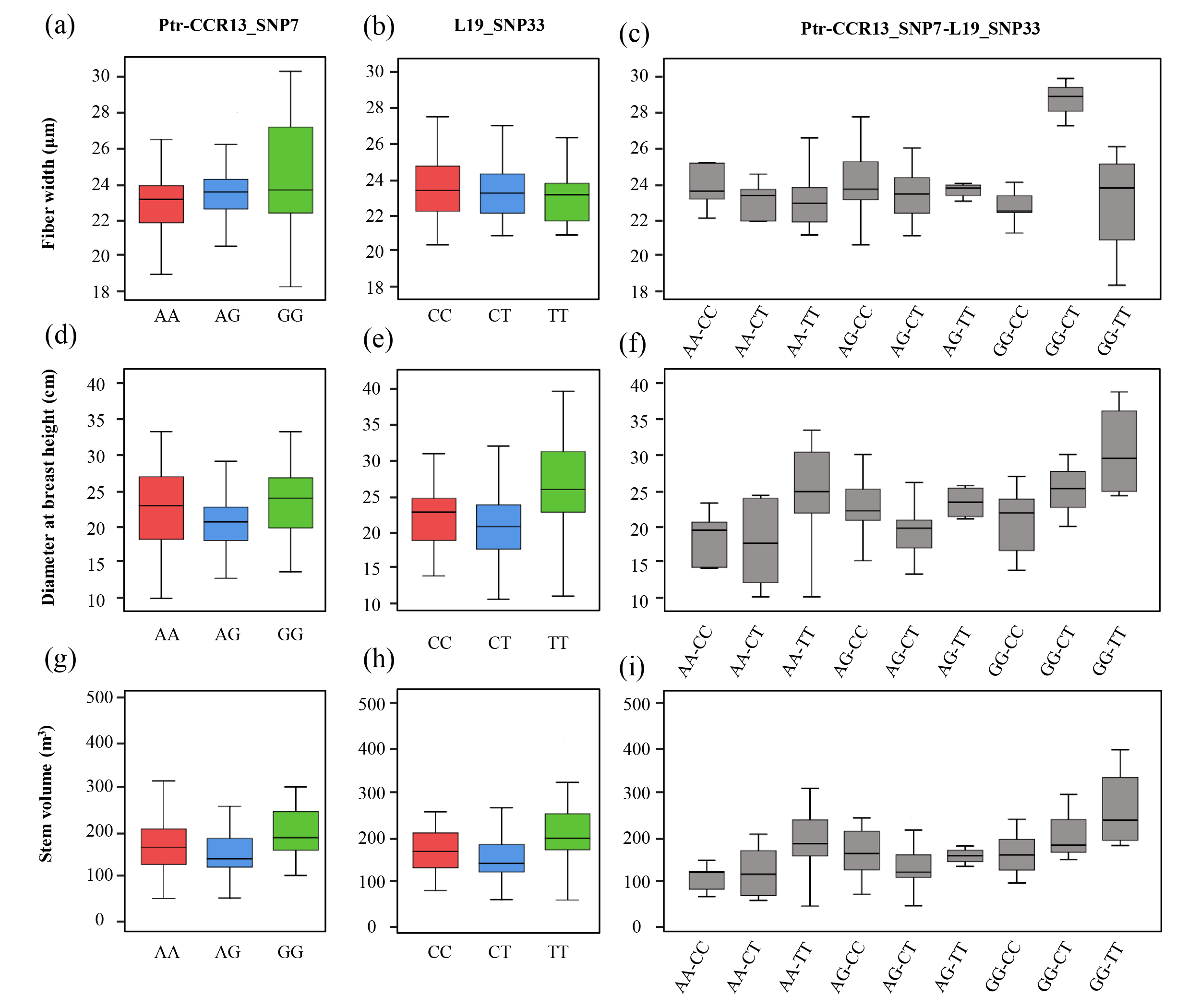
**Figure S7. The epistatic interactions of SNP pairs in the natural population of *P. tomentosa*.** Epistatic interactions of SNP pairs for fiber width (a-c), diameter at breast height (d-f), and stem volume (g-i) in the association population of *P. tomentosa*. (a-b, d-e, g-h) The single locus effects for the traits. (c, f, i) The epistatic effects of genotype combinations for the traits.


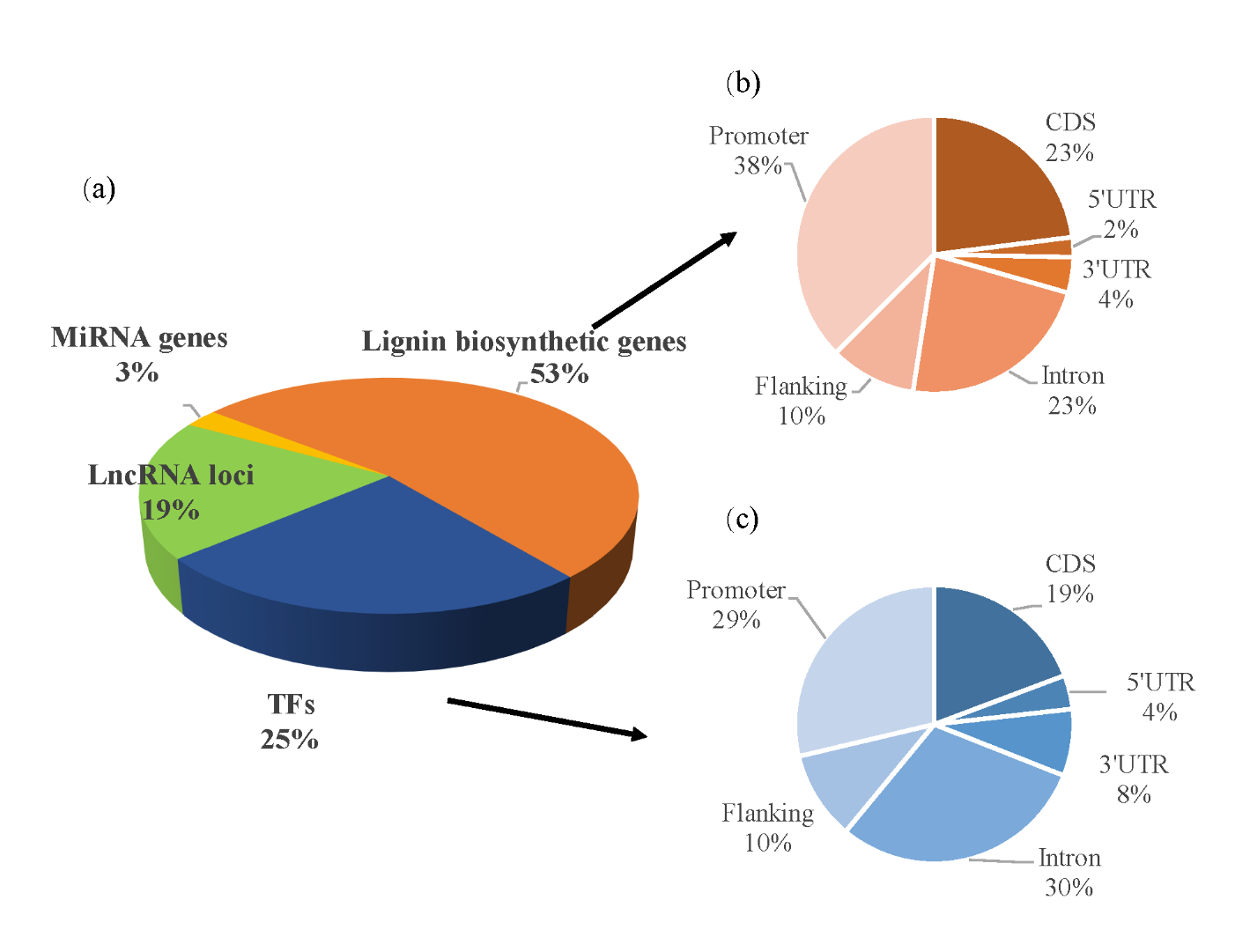


**Figure S8. Summary of the distribution of the eQTNs.** (a) The distribution of eQTNs in the four types of genetic factors. (b) The detailed distribution of eQTNs in lignin biosynthetic genes and transcription factor genes (c).


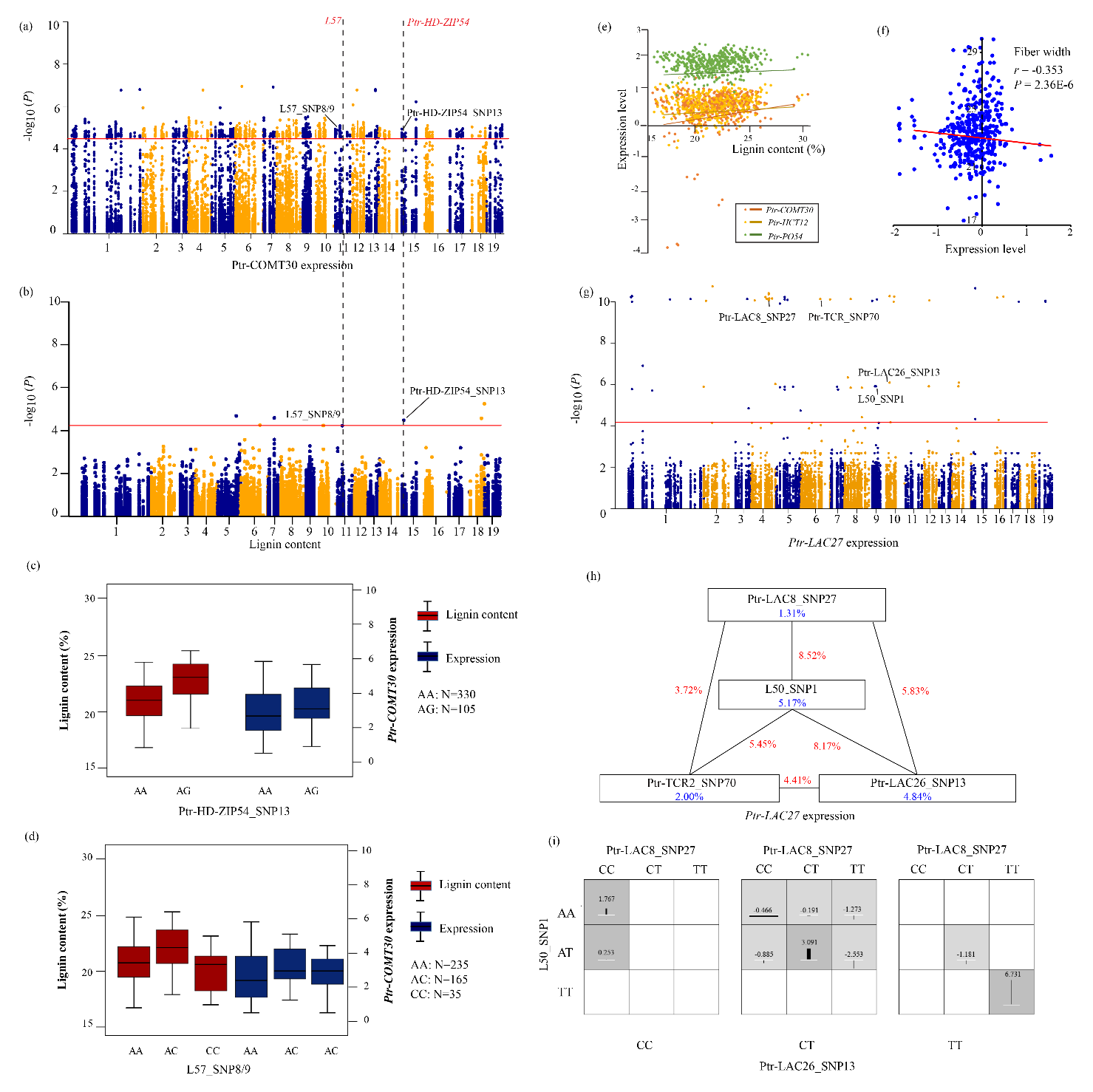


**Figure S9.** **The interpretation of causal SNPs for phenotypes by eQTNs.** (a) Manhattan plot displaying the association results for *Ptr-COMT30* expression and lignin content. (b) The overlapped associated loci are marked with dashed lines. (c–d) The genotype effects of Ptr-HD-ZIP54_SNP13 and L57_SNP8 for lignin content and *Ptr-COMT30* expressions, respectively. We discarded L57_SNP9 as it was in LD with L57_SNP8. (e) Plot of correlations between lignin content and normalized expression levels of *Ptr-COMT30*, *Ptr-HCT12*, and *Ptr-PO54*. The *r* value indicated Pearson’s correlation coefficient. (f) Plot of correlations between fiber width and normalized expression levels of *Ptr-LAC27*. (g) Manhattan plot displaying the association results for *Ptr-LAC27* expression marking the causal loci with epistatic interactions. (h) The epistatic effects of four loci for the variations in expression of *LAC-27*. The blue values indicate the single variant effects and the red values represent the pairwise effects. (i) Box plots revealing the epistatic effects of different genotypic combinations for phenotypic variations of *Ptr-LAC27* expression.

**Table S1.** Pearson’s correlation coefficients for each lncRNA-mRNA pair.

| **LncRNA ID** | **Target Gene Model** | **Gene ID** | **Type** | **Coefficients** |
| --- | --- | --- | --- | --- |
| TCONS_00003569 | Potri.001G054600 | *Ptr-LAC1* | *cis* | 0.588** |
| TCONS_00004219 | Potri.001G145800 | *Ptr-PO9* | *cis* | 0.986** |
| TCONS_00004219 | Potri.001G145800 | *Ptr-PO9* | *trans* | 0.986** |
| TCONS_00004223 | Potri.001G145800 | *Ptr-PO9* | *cis* | -0.594** |
| TCONS_00004224 | Potri.001G145800 | *Ptr-PO9* | *cis* | -0.577** |
| TCONS_00006484 | Potri.001G458700 | *Ptr-PO13* | *cis* | -0.369** |
| TCONS_00010737 | Potri.001G145800 | *Ptr-PO9* | *cis* | 0.954** |
| TCONS_00011246 | Potri.001G341600 | *Ptr-LAC5* | *trans* | 0.466** |
| TCONS_00011246 | Potri.001G401300 | *Ptr-LAC7* | *cis* | 0.994** |
| TCONS_00011246 | Potri.001G401300 | *Ptr-LAC7* | *trans* | 0.994** |
| TCONS_00011246 | Potri.011G120300 | *Ptr-LAC30* | *trans* | 0.995** |
| TCONS_00011246 | Potri.001G054600 | *Ptr-LAC1* | *trans* | 0.999** |
| TCONS_00014447 | Potri.001G036900 | *Ptr-4CL3* | *cis* | 0.059 |
| TCONS_00014485 | Potri.001G054600 | *Ptr-LAC1* | *cis* | -0.702** |
| TCONS_00014486 | Potri.001G054600 | *Ptr-LAC1* | *cis* | 0.759** |
| TCONS_00014702 | Potri.001G145800 | *Ptr-PO9* | *cis* | 0.783** |
| TCONS_00016920 | Potri.001G145800 | *Ptr-PO9* | *cis* | 0.932** |
| TCONS_00017033 | Potri.001G184300 | *Ptr-LAC2* | *cis* | -0.272** |
| TCONS_00020491 | Potri.003G214900 | *Ptr-PO23* | *trans* | 1.000** |
| TCONS_00020570 | Potri.001G042900 | *Ptr-HCT6* | *cis* | -0.119* |
| TCONS_00020570 | Potri.001G042900 | *Ptr-HCT6* | *trans* | -0.119* |
| TCONS_00020570 | Potri.003G183900 | *Ptr-HCT1* | *trans* | -0.089 |
| TCONS_00020604 | Potri.001G054600 | *Ptr-LAC1* | *cis* | 0.700** |
| TCONS_00020605 | Potri.001G054600 | *Ptr-LAC1* | *cis* | 0.056 |
| TCONS_00020841 | Potri.001G145800 | *Ptr-PO9* | *cis* | 0.944** |
| TCONS_00022861 | Potri.001G145800 | *Ptr-PO9* | *cis* | -0.480** |
| TCONS_00023485 | Potri.001G451100 | *Ptr-COMT25* | *cis* | -0.376** |
| TCONS_00023485 | Potri.001G451100 | *Ptr-COMT25* | *trans* | -0.376** |
| TCONS_00025559 | Potri.001G054600 | *Ptr-LAC1* | *cis* | -0.739** |
| TCONS_00028217 | Potri.001G042900 | *Ptr-HCT6* | *cis* | 0.018 |
| TCONS_00028217 | Potri.001G042900 | *Ptr-HCT6* | *trans* | 0.018 |
| TCONS_00028217 | Potri.003G183900 | *Ptr-HCT1* | *trans* | 0.732** |
| TCONS_00030681 | Potri.003G183900 | *Ptr-HCT1* | *trans* | 0.457** |
| TCONS_00030681 | Potri.001G042900 | *Ptr-HCT6* | *cis* | 0.920** |
| TCONS_00030681 | Potri.001G042900 | *Ptr-HCT6* | *trans* | 0.920** |
| TCONS_00031093 | Potri.001G145800 | *Ptr-PO9* | *cis* | 0.158* |
| TCONS_00031094 | Potri.001G145800 | *Ptr-PO9* | *cis* | 0.920** |
| TCONS_00031095 | Potri.001G145800 | *Ptr-PO9* | *cis* | 0.961** |
| TCONS_00033373 | Potri.002G004100 | *Ptr-CCR10* | *cis* | -0.825** |
| TCONS_00036686 | Potri.014G106600 | *Ptr-COMT3* | *trans* | -0.388** |
| TCONS_00036686 | Potri.002G180600 | *Ptr-COMT4* | *cis* | 1.000** |
| TCONS_00036686 | Potri.002G180600 | *Ptr-COMT4* | *trans* | 1.000** |
| TCONS_00036686 | Potri.002G180500 | *Ptr-COMT27* | *cis* | 1.000** |
| TCONS_00036686 | Potri.002G180500 | *Ptr-COMT27* | *trans* | 1.000** |
| TCONS_00036687 | Potri.014G106600 | *Ptr-COMT3* | *trans* | -0.388** |
| TCONS_00036687 | Potri.002G180500 | *Ptr-COMT27* | *cis* | 1.000** |
| TCONS_00036687 | Potri.002G180500 | *Ptr-COMT27* | *trans* | 1.000** |
| TCONS_00036687 | Potri.002G180600 | *Ptr-COMT4* | *cis* | 1.000** |
| TCONS_00036687 | Potri.002G180600 | *Ptr-COMT4* | *trans* | 1.000** |
| TCONS_00036688 | Potri.014G106600 | *Ptr-COMT3* | *trans* | -0.527** |
| TCONS_00036688 | Potri.002G180600 | *Ptr-COMT4* | *cis* | 0.999** |
| TCONS_00036688 | Potri.002G180600 | *Ptr-COMT4* | *trans* | 0.999** |
| TCONS_00036688 | Potri.002G180500 | *Ptr-COMT27* | *cis* | 1.000** |
| TCONS_00036688 | Potri.002G180500 | *Ptr-COMT27* | *trans* | 1.000** |
| TCONS_00038885 | Potri.014G106600 | *Ptr-COMT3* | *trans* | -0.437** |
| TCONS_00038885 | Potri.002G180500 | *Ptr-COMT27* | *cis* | 1.000** |
| TCONS_00038885 | Potri.002G180500 | *Ptr-COMT27* | *trans* | 1.000** |
| TCONS_00038885 | Potri.002G180600 | *Ptr-COMT4* | *cis* | 1.000** |
| TCONS_00038885 | Potri.002G180600 | *Ptr-COMT4* | *trans* | 1.000** |
| TCONS_00038887 | Potri.014G106600 | *Ptr-COMT3* | *trans* | -0.378** |
| TCONS_00038887 | Potri.002G180600 | *Ptr-COMT4* | *trans* | 1.000** |
| TCONS_00038887 | Potri.002G180500 | *Ptr-COMT27* | *trans* | 1.000** |
| TCONS_00038904 | Potri.002G183600 | *Ptr-CCoAOMT6* | *cis* | -0.530** |
| TCONS_00038904 | Potri.002G183600 | *Ptr-CCoAOMT6* | *trans* | -0.530** |
| TCONS_00038905 | Potri.002G183600 | *Ptr-CCoAOMT6* | *cis* | 0.517** |
| TCONS_00038905 | Potri.002G183600 | *Ptr-CCoAOMT6* | *trans* | 0.517** |
| TCONS_00042683 | Potri.002G183600 | *Ptr-CCoAOMT6* | *trans* | -0.528** |
| TCONS_00044238 | Potri.014G106600 | *Ptr-COMT3* | *trans* | -0.385** |
| TCONS_00044238 | Potri.002G180600 | *Ptr-COMT4* | *cis* | 1.000** |
| TCONS_00044238 | Potri.002G180600 | *Ptr-COMT4* | *trans* | 1.000** |
| TCONS_00044238 | Potri.002G180500 | *Ptr-COMT27* | *cis* | 1.000** |
| TCONS_00044238 | Potri.002G180500 | *Ptr-COMT27* | *trans* | 1.000** |
| TCONS_00044242 | Potri.014G106600 | *Ptr-COMT3* | *trans* | -0.390** |
| TCONS_00044242 | Potri.002G180500 | *Ptr-COMT27* | *trans* | 1.000** |
| TCONS_00044242 | Potri.002G180600 | *Ptr-COMT4* | *trans* | 1.000** |
| TCONS_00046024 | Potri.005G257700 | *Ptr-CCR25* | *trans* | -0.487** |
| TCONS_00046024 | Potri.002G004100 | *Ptr-CCR10* | *trans* | -0.313** |
| TCONS_00046510 | Potri.014G106600 | *Ptr-COMT3* | *trans* | -0.391** |
| TCONS_00046510 | Potri.002G180500 | *Ptr-COMT27* | *cis* | 1.000** |
| TCONS_00046510 | Potri.002G180500 | *Ptr-COMT27* | *trans* | 1.000** |
| TCONS_00046510 | Potri.002G180600 | *Ptr-COMT4* | *trans* | 1.000** |
| TCONS_00048406 | Potri.002G183600 | *Ptr-CCoAOMT6* | *cis* | 0.683** |
| TCONS_00048406 | Potri.002G183600 | *Ptr-CCoAOMT6* | *trans* | 0.683** |
| TCONS_00049388 | Potri.002G183600 | *Ptr-CCoAOMT6* | *trans* | 0.678** |
| TCONS_00049389 | Potri.002G183600 | *Ptr-CCoAOMT6* | *cis* | -0.305** |
| TCONS_00049389 | Potri.002G183600 | *Ptr-CCoAOMT6* | *trans* | -0.305** |
| TCONS_00050977 | Potri.002G183600 | *Ptr-CCoAOMT6* | *cis* | -0.264** |
| TCONS_00050977 | Potri.002G183600 | *Ptr-CCoAOMT6* | *trans* | -0.264** |
| TCONS_00052181 | Potri.005G195600 | *Ptr-PO36* | *trans* | -0.653** |
| TCONS_00052181 | Potri.005G195700 | *Ptr-PO37* | *trans* | -0.418** |
| TCONS_00052181 | Potri.002G065300 | *Ptr-PO16* | *cis* | 0.210** |
| TCONS_00052181 | Potri.002G065300 | *Ptr-PO16* | *trans* | 0.210** |
| TCONS_00052611 | Potri.002G180500 | *Ptr-COMT27* | *cis* | -0.341** |
| TCONS_00052630 | Potri.002G183600 | *Ptr-CCoAOMT6* | *cis* | 0.764** |
| TCONS_00052630 | Potri.002G183600 | *Ptr-CCoAOMT6* | *trans* | 0.764** |
| TCONS_00052631 | Potri.002G183600 | *Ptr-CCoAOMT6* | *cis* | 0.010 |
| TCONS_00052631 | Potri.002G183600 | *Ptr-CCoAOMT6* | *trans* | 0.010 |
| TCONS_00056430 | Potri.003G183900 | *Ptr-HCT1* | *cis* | 0.372** |
| TCONS_00058839 | Potri.003G099700 | *Ptr-4CL9* | *cis* | -0.842** |
| TCONS_00059933 | Potri.003G099700 | *Ptr-4CL9* | *cis* | 0.668** |
| TCONS_00060903 | Potri.003G099700 | *Ptr-4CL9* | *cis* | -0.521** |
| TCONS_00060904 | Potri.003G099700 | *Ptr-4CL9* | *cis* | -0.165* |
| TCONS_00061082 | Potri.003G183900 | *Ptr-HCT1* | *cis* | -0.603** |
| TCONS_00062052 | Potri.003G099700 | *Ptr-4CL9* | *cis* | -0.200* |
| TCONS_00066343 | Potri.003G099700 | *Ptr-4CL9* | *cis* | 0.966** |
| TCONS_00066343 | Potri.003G099700 | *Ptr-4CL9* | *trans* | 0.966** |
| TCONS_00066345 | Potri.003G099700 | *Ptr-4CL9* | *cis* | 0.976** |
| TCONS_00066345 | Potri.003G099700 | *Ptr-4CL9* | *trans* | 0.976** |
| TCONS_00066346 | Potri.003G099700 | *Ptr-4CL9* | *cis* | 0.990** |
| TCONS_00066346 | Potri.003G099700 | *Ptr-4CL9* | *trans* | 0.990** |
| TCONS_00066347 | Potri.003G099700 | *Ptr-4CL9* | *trans* | 1.000** |
| TCONS_00066495 | Potri.003G183900 | *Ptr-HCT1* | *cis* | -0.780** |
| TCONS_00067111 | Potri.003G099700 | *Ptr-4CL9* | *cis* | 0.990** |
| TCONS_00067111 | Potri.003G099700 | *Ptr-4CL9* | *trans* | 0.990** |
| TCONS_00068877 | Potri.003G053700 | *Ptr-PO17* | *cis* | 0.340** |
| TCONS_00069338 | Potri.003G183900 | *Ptr-HCT1* | *cis* | 0.123* |
| TCONS_00071476 | Potri.004G017600 | *Ptr-HCT8* | *cis* | -0.584** |
| TCONS_00073636 | Potri.004G017600 | *Ptr-HCT8* | *cis* | -0.009 |
| TCONS_00073639 | Potri.004G017600 | *Ptr-HCT8* | *cis* | -0.390** |
| TCONS_00075891 | Potri.004G017600 | *Ptr-HCT8* | *cis* | 0.988** |
| TCONS_00076209 | Potri.004G161600 | *Ptr-F5H4* | *cis* | 0.787** |
| TCONS_00077099 | Potri.004G144600 | *Ptr-PO30* | *cis* | 0.989** |
| TCONS_00077099 | Potri.004G144600 | *Ptr-PO30* | *trans* | 0.989** |
| TCONS_00080767 | Potri.004G017600 | *Ptr-HCT8* | *cis* | 0.992** |
| TCONS_00080767 | Potri.004G017600 | *Ptr-HCT8* | *trans* | 0.992** |
| TCONS_00080827 | Potri.004G050500 | *Ptr-COMT10* | *cis* | 0.999** |
| TCONS_00080827 | Potri.004G050500 | *Ptr-COMT10* | *trans* | 0.999** |
| TCONS_00080827 | Potri.011G059600 | *Ptr-COMT11* | *trans* | -0.700** |
| TCONS_00080827 | Potri.004G050400 | *Ptr-COMT9* | *cis* | 1.000** |
| TCONS_00088562 | Potri.005G108900 | *Ptr-PO33* | *cis* | -0.178* |
| TCONS_00088565 | Potri.005G108900 | *Ptr-PO33* | *cis* | -0.843** |
| TCONS_00088566 | Potri.005G108900 | *Ptr-PO33* | *cis* | -0.813** |
| TCONS_00089608 | Potri.005G257700 | *Ptr-CCR25* | *cis* | -0.446** |
| TCONS_00089609 | Potri.005G257700 | *Ptr-CCR25* | *cis* | -0.060 |
| TCONS_00092470 | Potri.005G257700 | *Ptr-CCR25* | *cis* | -0.100 |
| TCONS_00095780 | Potri.005G108900 | *Ptr-PO33* | *cis* | 0.848** |
| TCONS_00095781 | Potri.005G108900 | *Ptr-PO33* | *cis* | -0.839** |
| TCONS_00097134 | Potri.005G257700 | *Ptr-CCR25* | *cis* | -0.448** |
| TCONS_00099492 | Potri.005G200700 | *Ptr-LAC10* | *cis* | -0.573** |
| TCONS_00099492 | Potri.005G200700 | *Ptr-LAC10* | *trans* | -0.573** |
| TCONS_00099492 | Potri.005G200600 | *Ptr-LAC9* | *cis* | 1.000** |
| TCONS_00099492 | Potri.005G200600 | *Ptr-LAC9* | *trans* | 1.000** |
| TCONS_00099492 | Potri.019G088500 | *Ptr-LAC43* | *trans* | -0.539** |
| TCONS_00099492 | Potri.019G088600 | *Ptr-LAC44* | *trans* | -0.595** |
| TCONS_00099492 | Potri.001G206200 | *Ptr-LAC3* | *trans* | 1.000** |
| TCONS_00099492 | Potri.011G071100 | *Ptr-LAC28* | *trans* | 0.980** |
| TCONS_00099493 | Potri.005G200700 | *Ptr-LAC10* | *cis* | -0.573** |
| TCONS_00099493 | Potri.005G200700 | *Ptr-LAC10* | *trans* | -0.573** |
| TCONS_00099493 | Potri.019G088500 | *Ptr-LAC43* | *trans* | -0.539** |
| TCONS_00099493 | Potri.005G200600 | *Ptr-LAC9* | *cis* | 1.000** |
| TCONS_00099493 | Potri.019G088600 | *Ptr-LAC44* | *trans* | 1.000** |
| TCONS_00099493 | Potri.005G200600 | *Ptr-LAC9* | *trans* | -0.595** |
| TCONS_00099493 | Potri.011G071100 | *Ptr-LAC28* | *trans* | 0.985** |
| TCONS_00099629 | Potri.005G257700 | *Ptr-CCR25* | *cis* | 0.959** |
| TCONS_00101210 | Potri.005G243700 | *Ptr-CAD18* | *cis* | -0.748** |
| TCONS_00101824 | Potri.005G108900 | *Ptr-PO33* | *cis* | 0.541** |
| TCONS_00104764 | Potri.005G257700 | *Ptr-CCR25* | *cis* | -0.679** |
| TCONS_00105103 | Potri.005G108900 | *Ptr-PO33* | *cis* | 0.812** |
| TCONS_00107215 | Potri.007G139400 | *Ptr-HCT13* | *trans* | -0.402** |
| TCONS_00107215 | Potri.006G010300 | *Ptr-HCT11* | *cis* | 0.980** |
| TCONS_00107215 | Potri.006G010300 | *Ptr-HCT11* | *trans* | 0.980** |
| TCONS_00107218 | Potri.006G010300 | *Ptr-HCT11* | *cis* | 0.977** |
| TCONS_00107218 | Potri.006G010300 | *Ptr-HCT11* | *trans* | 0.977** |
| TCONS_00110962 | Potri.006G267400 | *Ptr-PO41* | *cis* | -0.943** |
| TCONS_00113600 | Potri.006G267400 | *Ptr-PO41* | *cis* | -0.604** |
| TCONS_00114236 | Potri.016G031100 | *Ptr-C3H2* | *trans* | -0.717** |
| TCONS_00114236 | Potri.006G033300 | *Ptr-C3H3* | *cis* | 0.979** |
| TCONS_00114236 | Potri.006G033300 | *Ptr-C3H3* | *trans* | 0.979** |
| TCONS_00116110 | Potri.006G199100 | *Ptr-CAD8* | *cis* | 1.000** |
| TCONS_00116289 | Potri.006G267400 | *Ptr-PO41* | *cis* | -0.981** |
| TCONS_00121060 | Potri.006G033300 | *Ptr-C3H3* | *cis* | 0.883** |
| TCONS_00121062 | Potri.006G033300 | *Ptr-C3H3* | *cis* | 0.858** |
| TCONS_00122769 | Potri.006G024300 | *Ptr-CAD16* | *cis* | -0.226** |
| TCONS_00125387 | Potri.006G010300 | *Ptr-HCT11* | *cis* | 0.999** |
| TCONS_00125387 | Potri.006G010300 | *Ptr-HCT11* | *trans* | 0.999** |
| TCONS_00126792 | Potri.007G019300 | *Ptr-PO42* | *cis* | -0.241** |
| TCONS_00127123 | Potri.007G067200 | *Ptr-PO43* | *cis* | 0.092 |
| TCONS_00127616 | Potri.007G139400 | *Ptr-HCT13* | *cis* | -0.333** |
| TCONS_00127617 | Potri.007G139400 | *Ptr-HCT13* | *cis* | 0.988** |
| TCONS_00128181 | Potri.007G019300 | *Ptr-PO42* | *cis* | -0.288** |
| TCONS_00128964 | Potri.007G019300 | *Ptr-PO42* | *cis* | 0.986** |
| TCONS_00129611 | Potri.007G023300 | *Ptr-LAC16* | *cis* | -0.193* |
| TCONS_00129787 | Potri.007G096200 | *Ptr-PO46* | *cis* | 0.897** |
| TCONS_00130261 | Potri.007G019300 | *Ptr-PO42* | *cis* | 0.999** |
| TCONS_00130978 | Potri.007G019300 | *Ptr-PO42* | *cis* | -0.969** |
| TCONS_00132352 | Potri.007G067200 | *Ptr-PO43* | *cis* | -0.725** |
| TCONS_00132354 | Potri.007G067200 | *Ptr-PO43* | *cis* | -0.131* |
| TCONS_00132884 | Potri.007G019300 | *Ptr-PO42* | *cis* | -0.698** |
| TCONS_00132885 | Potri.007G019300 | *Ptr-PO42* | *cis* | 0.614** |
| TCONS_00132994 | Potri.007G067200 | *Ptr-PO43* | *cis* | -0.449** |
| TCONS_00133417 | Potri.007G096200 | *Ptr-PO46* | *cis* | 0.494** |
| TCONS_00135410 | Potri.007G019300 | *Ptr-PO42* | *cis* | 0.989** |
| TCONS_00135622 | Potri.007G067200 | *Ptr-PO43* | *cis* | -0.725** |
| TCONS_00135623 | Potri.007G067200 | *Ptr-PO43* | *cis* | -0.292** |
| TCONS_00135717 | Potri.007G096200 | *Ptr-PO46* | *cis* | -0.485** |
| TCONS_00136085 | Potri.007G019300 | *Ptr-PO42* | *cis* | 0.999** |
| TCONS_00138144 | Potri.008G038200 | *Ptr-PAL2* | *cis* | 0.885** |
| TCONS_00143999 | Potri.008G110600 | *Ptr-PO55* | *cis* | -0.574** |
| TCONS_00146032 | Potri.008G038200 | *Ptr-PAL2* | *cis* | -0.615** |
| TCONS_00148002 | Potri.008G038200 | *Ptr-PAL2* | *cis* | 0.138* |
| TCONS_00149417 | Potri.008G038200 | *Ptr-PAL2* | *cis* | 0.226** |
| TCONS_00149563 | Potri.008G110600 | *Ptr-PO55* | *cis* | 0.884** |
| TCONS_00150322 | Potri.008G110600 | *Ptr-PO55* | *cis* | -0.127* |
| TCONS_00152392 | Potri.008G110600 | *Ptr-PO55* | *cis* | -0.497** |
| TCONS_00153160 | Potri.008G110600 | *Ptr-PO55* | *cis* | -0.333** |
| TCONS_00155396 | Potri.009G123600 | *Ptr-F5H3* | *cis* | -0.026 |
| TCONS_00155581 | Potri.001G184300 | *Ptr-LAC2* | *trans* | 0.902** |
| TCONS_00156611 | Potri.009G123600 | *Ptr-F5H3* | *cis* | 0.598** |
| TCONS_00156688 | Potri.001G184300 | *Ptr-LAC2* | *trans* | 0.620** |
| TCONS_00157438 | Potri.001G184300 | *Ptr-LAC2* | *trans* | 0.793** |
| TCONS_00158035 | Potri.009G076300 | *Ptr-CCR9* | *cis* | 0.777** |
| TCONS_00158035 | Potri.009G076300 | *Ptr-CCR9* | *trans* | 0.777** |
| TCONS_00160255 | Potri.009G076300 | *Ptr-CCR9* | *cis* | 0.517** |
| TCONS_00160256 | Potri.009G076300 | *Ptr-CCR9* | *cis* | 0.415** |
| TCONS_00160352 | Potri.009G139800 | *Ptr-COMT15* | *cis* | 0.667** |
| TCONS_00161733 | Potri.009G095800 | *Ptr-CAD1* | *cis* | -0.879** |
| TCONS_00161734 | Potri.009G095800 | *Ptr-CAD1* | *cis* | 0.049 |
| TCONS_00164642 | Potri.009G076300 | *Ptr-CCR9* | *cis* | 0.413** |
| TCONS_00164817 | Potri.009G123600 | *Ptr-F5H3* | *cis* | -0.215** |
| TCONS_00168298 | Potri.010G134500 | *Ptr-PO58* | *cis* | -0.659** |
| TCONS_00172906 | Potri.010G057000 | *Ptr-4CL14* | *cis* | 0.551** |
| TCONS_00178109 | Potri.010G134500 | *Ptr-PO58* | *cis* | -0.409** |
| TCONS_00182353 | Potri.010G230200 | *Ptr-4CL13* | *cis* | -0.409** |
| TCONS_00183478 | Potri.010G036100 | *Ptr-PO57* | *cis* | 0.620** |
| TCONS_00183563 | Potri.010G057000 | *Ptr-4CL14* | *cis* | -0.803** |
| TCONS_00184211 | Potri.010G230200 | *Ptr-4CL13* | *cis* | 0.881** |
| TCONS_00186169 | Potri.004G052100 | *Ptr-PO27* | *trans* | -0.284** |
| TCONS_00186169 | Potri.011G062300 | *Ptr-PO64* | *cis* | 0.939** |
| TCONS_00186169 | Potri.011G062300 | *Ptr-PO64* | *trans* | 0.939** |
| TCONS_00187699 | Potri.001G341600 | *Ptr-LAC5* | *trans* | 0.665** |
| TCONS_00187699 | Potri.001G401300 | *Ptr-LAC7* | *trans* | 0.939** |
| TCONS_00187699 | Potri.011G120300 | *Ptr-LAC30* | *cis* | 0.941** |
| TCONS_00187699 | Potri.011G120300 | *Ptr-LAC30* | *trans* | 0.941** |
| TCONS_00187699 | Potri.001G054600 | *Ptr-LAC1* | *trans* | 0.980** |
| TCONS_00189620 | Potri.001G341600 | *Ptr-LAC5* | *trans* | 0.308** |
| TCONS_00189620 | Potri.001G054600 | *Ptr-LAC1* | *trans* | 0.977** |
| TCONS_00189620 | Potri.011G120300 | *Ptr-LAC30* | *cis* | 0.997** |
| TCONS_00189620 | Potri.011G120300 | *Ptr-LAC30* | *trans* | 0.997** |
| TCONS_00189620 | Potri.001G401300 | *Ptr-LAC7* | *trans* | 0.998** |
| TCONS_00193338 | Potri.001G341600 | *Ptr-LAC5* | *trans* | 0.543** |
| TCONS_00193338 | Potri.001G401300 | *Ptr-LAC7* | *trans* | 0.977** |
| TCONS_00193338 | Potri.011G120300 | *Ptr-LAC30* | *cis* | 0.979** |
| TCONS_00193338 | Potri.011G120300 | *Ptr-LAC30* | *trans* | 0.979** |
| TCONS_00193338 | Potri.001G054600 | *Ptr-LAC1* | *trans* | 0.996** |
| TCONS_00197420 | Potri.012G095000 | *Ptr-4CL17* | *cis* | 0.780** |
| TCONS_00197420 | Potri.012G095000 | *Ptr-4CL17* | *trans* | 0.780** |
| TCONS_00198407 | Potri.012G095000 | *Ptr-4CL17* | *cis* | 0.712** |
| TCONS_00198407 | Potri.012G095000 | *Ptr-4CL17* | *trans* | 0.712** |
| TCONS_00204979 | Potri.012G095000 | *Ptr-4CL17* | *trans* | 0.645** |
| TCONS_00204982 | Potri.012G095000 | *Ptr-4CL17* | *cis* | 0.802** |
| TCONS_00204982 | Potri.012G095000 | *Ptr-4CL17* | *trans* | 0.802** |
| TCONS_00207615 | Potri.013G156800 | *Ptr-PO73* | *cis* | -0.784** |
| TCONS_00209346 | Potri.013G152700 | *Ptr-LAC32* | *cis* | 0.808** |
| TCONS_00210073 | Potri.013G156800 | *Ptr-PO73* | *cis* | 0.244** |
| TCONS_00211536 | Potri.013G152700 | *Ptr-LAC32* | *cis* | -0.409** |
| TCONS_00212825 | Potri.013G152700 | *Ptr-LAC32* | *cis* | -0.026 |
| TCONS_00213789 | Potri.013G152700 | *Ptr-LAC32* | *cis* | 0.191* |
| TCONS_00213790 | Potri.013G152700 | *Ptr-LAC32* | *cis* | -0.074 |
| TCONS_00216541 | Potri.013G120800 | *Ptr-COMT13* | *cis* | 0.998** |
| TCONS_00216541 | Potri.013G120800 | *Ptr-COMT13* | *trans* | 0.998** |
| TCONS_00216541 | Potri.013G121400 | *Ptr-COMT18* | *trans* | 0.999** |
| TCONS_00216541 | Potri.013G122500 | *Ptr-COMT19* | *trans* | 0.999** |
| TCONS_00216541 | Potri.013G121800 | *Ptr-COMT16* | *trans* | 1.000** |
| TCONS_00218750 | Potri.014G100600 | *Ptr-LAC33* | *cis* | -0.002 |
| TCONS_00218751 | Potri.014G100600 | *Ptr-LAC33* | *cis* | -0.380** |
| TCONS_00220411 | Potri.014G100600 | *Ptr-LAC33* | *cis* | 0.382** |
| TCONS_00222296 | Potri.014G100600 | *Ptr-LAC33* | *cis* | 0.252** |
| TCONS_00222297 | Potri.014G100600 | *Ptr-LAC33* | *cis* | 0.980** |
| TCONS_00228084 | Potri.014G100600 | *Ptr-LAC33* | *cis* | 0.538** |
| TCONS_00235183 | Potri.015G003600 | *Ptr-PO76* | *cis* | -0.281** |
| TCONS_00237001 | Potri.015G003600 | *Ptr-PO76* | *cis* | -0.067 |
| TCONS_00239128 | Potri.015G003600 | *Ptr-PO76* | *cis* | -0.515** |
| TCONS_00242738 | Potri.006G033300 | *Ptr-C3H3* | *trans* | -0.576** |
| TCONS_00242738 | Potri.016G031100 | *Ptr-C3H2* | *cis* | 0.792** |
| TCONS_00242738 | Potri.016G031100 | *Ptr-C3H2* | *trans* | 0.792** |
| TCONS_00246863 | Potri.016G031100 | *Ptr-C3H2* | *cis* | 0.962** |
| TCONS_00246863 | Potri.016G031100 | *Ptr-C3H2* | *trans* | 0.962** |
| TCONS_00246864 | Potri.016G031100 | *Ptr-C3H2* | *cis* | 0.962** |
| TCONS_00246864 | Potri.016G031100 | *Ptr-C3H2* | *trans* | 0.962** |
| TCONS_00247460 | Potri.016G031100 | *Ptr-C3H2* | *cis* | 0.962** |
| TCONS_00248207 | Potri.011G150500 | *Ptr-COMT30* | *trans* | 0.899** |
| TCONS_00248936 | Potri.016G031100 | *Ptr-C3H2* | *cis* | 0.962** |
| TCONS_00249407 | Potri.016G031100 | *Ptr-C3H2* | *cis* | 0.955** |
| TCONS_00249497 | Potri.001G451100 | *Ptr-COMT25* | *trans* | -0.622** |
| TCONS_00249497 | Potri.011G150500 | *Ptr-COMT30* | *trans* | -0.258** |
| TCONS_00263286 | Potri.018G109900 | *Ptr-HCT19* | *cis* | -0.333** |
| TCONS_00265836 | Potri.018G109900 | *Ptr-HCT19* | *cis* | -0.742** |
| TCONS_00266501 | Potri.018G109900 | *Ptr-HCT19* | *cis* | 0.996** |
| TCONS_00267276 | Potri.018G109900 | *Ptr-HCT19* | *cis* | 0.278** |
| TCONS_00267807 | Potri.018G109900 | *Ptr-HCT19* | *cis* | -0.564** |
| TCONS_00269594 | Potri.018G109900 | *Ptr-HCT19* | *cis* | 0.979** |
| TCONS_00271007 | Potri.018G136900 | *Ptr-PO90* | *cis* | 0.999** |
| TCONS_00275478 | Potri.019G102900 | *Ptr-COMT24* | *cis* | 0.843** |
| TCONS_00277604 | Potri.019G088600 | *Ptr-LAC44* | *cis* | -0.595** |
| TCONS_00277604 | Potri.019G088500 | *Ptr-LAC43* | *cis* | -0.539** |
| TCONS_00280147 | Potri.019G102900 | *Ptr-COMT24* | *cis* | -0.059 |
| Negative correlations are marked by "−" ; *P < 0.05 level of significance; ** P < 0.01 level of significance. | | | | |

**Table S2.** Pearson’s correlation coefficients for each miRNA-mRNA pairs.

| **MiRNA ID** | **Target Gene Model** | **Gene ID** | **Coefficients** |
| --- | --- | --- | --- |
| Pto-miR160e | Potri.003G051700 | *Ptr-COMT6* | -0.189** |
| Pto-miR167a | Potri.006G267400 | *Ptr-PO41* | 0.963** |
| Pto-miR167b | Potri.006G267400 | *Ptr-PO41* | 0.997** |
| Pto-miR167c | Potri.006G267400 | *Ptr-PO41* | 0.999** |
| Pto-miR167d | Potri.006G267400 | *Ptr-PO41* | 1.000** |
| Pto-miR167e | Potri.006G267400 | *Ptr-PO41* | 1.000** |
| Pto-miR167f | Potri.006G267400 | *Ptr-PO41* | 1.000** |
| Pto-miR167g | Potri.006G267400 | *Ptr-PO41* | 1.000** |
| Pto-miR167h | Potri.001G054600 | *Ptr-LAC1* | -0.633** |
| Pto-miR167h | Potri.001G401300 | *Ptr-LAC7* | -0.597** |
| Pto-miR172e | Potri.013G136400 | *Ptr-COMT22* | -0.427** |
| Pto-miR172e | Potri.019G102900 | *Ptr-COMT24* | -0.427** |
| Pto-miR172g | Potri.013G136400 | *Ptr-COMT22* | -0.477** |
| Pto-miR172g | Potri.019G102900 | *Ptr-COMT24* | -0.477** |
| Pto-miR172h | Potri.013G136400 | *Ptr-COMT22* | -0.477** |
| Pto-miR172h | Potri.019G102900 | *Ptr-COMT24* | -0.477** |
| Pto-miR394a | Potri.003G059200 | *Ptr-CSE2* | -0.960** |
| Pto-miR394b | Potri.003G059200 | *Ptr-CSE2* | -0.272** |
| Pto-miR396g | Potri.010G183500 | *Ptr-LAC25* | -0.430** |
| Pto-miR397a | Potri.001G054600 | *Ptr-LAC1* | -0.941** |
| Pto-miR397a | Potri.001G184300 | *Ptr-LAC2* | -0.868** |
| Pto-miR397a | Potri.001G248700 | *Ptr-LAC4* | -0.909** |
| Pto-miR397a | Potri.001G401300 | *Ptr-LAC7* | -0.926** |
| Pto-miR397a | Potri.004G156400 | *Ptr-LAC8* | -0.885** |
| Pto-miR397a | Potri.006G094100 | *Ptr-LAC13* | 0.829** |
| Pto-miR397a | Potri.008G073800 | *Ptr-LAC19* | -0.911** |
| Pto-miR397a | Potri.009G042500 | *Ptr-LAC21* | -0.937** |
| Pto-miR397a | Potri.009G102700 | *Ptr-LAC22* | -0.925** |
| Pto-miR397a | Potri.010G183600 | *Ptr-LAC26* | -0.897** |
| Pto-miR397a | Potri.011G120300 | *Ptr-LAC30* | -0.926** |
| Pto-miR397a | Potri.016G106000 | *Ptr-LAC37* | 0.251** |
| Pto-miR397a | Potri.016G112000 | *Ptr-LAC40* | -0.943** |
| Pto-miR397b | Potri.001G054600 | *Ptr-LAC1* | -0.591** |
| Pto-miR397b | Potri.001G184300 | *Ptr-LAC2* | -0.558** |
| Pto-miR397b | Potri.001G248700 | *Ptr-LAC4* | -0.557** |
| Pto-miR397b | Potri.001G401300 | *Ptr-LAC7* | -0.578** |
| Pto-miR397b | Potri.004G156400 | *Ptr-LAC8* | -0.546** |
| Pto-miR397b | Potri.006G094100 | *Ptr-LAC13* | 1.000** |
| Pto-miR397b | Potri.008G073800 | *Ptr-LAC19* | -0.526** |
| Pto-miR397b | Potri.009G042500 | *Ptr-LAC21* | -0.582** |
| Pto-miR397b | Potri.009G102700 | *Ptr-LAC22* | -0.570** |
| Pto-miR397b | Potri.010G183600 | *Ptr-LAC26* | -0.565** |
| Pto-miR397b | Potri.011G120300 | *Ptr-LAC30* | -0.576** |
| Pto-miR397b | Potri.016G106000 | *Ptr-LAC37* | -0.333** |
| Pto-miR397b | Potri.016G112000 | *Ptr-LAC40* | -0.601** |
| Pto-miR408 | Potri.008G073800 | *Ptr-LAC19* | -0.563** |
| Pto-miR408 | Potri.010G183500 | *Ptr-LAC25* | -0.536** |
| Pto-miR408 | Potri.010G183600 | *Ptr-LAC26* | -0.602** |
| Pto-miR408 | Potri.013G152700 | *Ptr-LAC32* | -0.721** |
| Pto-miR475a | Potri.010G183500 | *Ptr-LAC25* | -0.642** |
| Pto-miR475b | Potri.010G183500 | *Ptr-LAC25* | -0.622** |
| Pto-miR475c | Potri.018G094200 | *Ptr-4CL2* | -0.590** |
| Pto-miR475d | Potri.010G183500 | *Ptr-LAC25* | -0.631** |
| Pto-miR482c | Potri.002G065300 | *Ptr-PO16* | -0.692** |
| Pto-miR482d | Potri.001G175000 | *Ptr-CSE1* | -0.256** |
| Pto-miR6425a | Potri.008G034200 | *Ptr-HCT16* | 1.000** |
| Pto-miR6425b | Potri.008G034200 | *Ptr-HCT16* | 0.671** |
| Pto-miR6425c | Potri.008G034200 | *Ptr-HCT16* | 1.000** |
| Pto-miR6425d | Potri.008G034200 | *Ptr-HCT16* | 0.725** |
| Pto-miR6425e | Potri.008G034200 | *Ptr-HCT16* | 1.000** |
| Pto-miR6427 | Potri.011G150500 | *Ptr-COMT30* | -0.569** |
| Pto-miR6427 | Potri.015G003600 | *Ptr-PO76* | -0.381** |
| Pto-miR6439a | Potri.004G052100 | *Ptr-PO27* | -0.440** |
| Pto-miR6443 | Potri.005G117500 | *Ptr-F5H1* | -0.484** |
| Pto-miR6443 | Potri.007G016400 | *Ptr-F5H2* | -0.476** |
| Pto-miR6446 | Potri.004G156400 | *Ptr-LAC8* | -0.546** |
| Pto-miR6452 | Potri.009G057600 | *Ptr-CCR30* | -0.654** |
| Pto-miR6456 | Potri.016G132800 | *Ptr-PO82* | -0.333** |
| Pto-miR6462 | Potri.001G175000 | *Ptr-CSE1* | -0.256** |
| Negative correlations are marked by "−" ; *P < 0.05 level of significance; ** P < 0.01 level of significance. | | | |

**Table S3.** The enriched transcription factor binding motifs in the promoters of lignin biosynthesis genes.

| **TFBS ID** | **Family of TFBS** | **Percentage (%)^†^** | **Copy numbers in each genes** | **The correspond TF genes associated with the TFBS** |
| --- | --- | --- | --- | --- |
| TFBS_0289 | HD-ZIP | 90.6 | 1-8 | Potri.001G229700; Potri.002G113400; Potri.002G136400; Potri.003G079800; Potri.005G147100; Potri.006G071600; Potri.006G193700; Potri.007G008200; Potri.008G129500; Potri.009G023600; Potri.014G045100; Potri.016G059000 |
| TFBS_0323 | Myb/SANT | 90.6 | 1-6 | Potri.004G074300; Potri.012G038300; Potri.015G030400 |
| TFBS_0540 | HD-ZIP | 91.1 | 1-12 | Potri.002G100600; Potri.005G071900; Potri.005G161500; Potri.008G148200; Potri.008G194400; Potri.010G093400; Potri.012G070900; Potri.015G065400; Potri.017G081700 |
| TFBS_0144 | AT-Hook | 91.1 | 1-16 | Potri.002G105500; Potri.005G156100 |
| TFBS_0154 | AT-Hook | 91.6 | 1-32 | Potri.002G105500; Potri.005G156100 |
| TFBS_0174 | bHLH | 90.1 | 1-15 | Potri.002G235400; Potri.002G248500; Potri.003G164500; Potri.004G099400; Potri.004G156000; Potri.005G121900; Potri.017G115300; Potri.005G146500; Potri.006G057200; Potri.006G135600; Potri.007G023600; Potri.008G113200; Potri.008G190800; Potri.009G117300; Potri.010G040000; Potri.010G136100; Potri.012G055700; Potri.012G072700; Potri.012G104900; Potri.014G148900; Potri.015G068100; Potri.015G104200; Potri.018G109500 |
| TFBS_0517 | HD-ZIP | 92.1 | 1-12 | Potri.002G100600; Potri.005G071900; Potri.005G161500; Potri.008G148200; Potri.008G194400; Potri.010G093400; Potri.012G070900; Potri.015G065400; Potri.017G081700 |
| TFBS_0471 | HD-ZIP | 92.1 | 1-11 | Potri.002G100600; Potri.005G071900; Potri.005G161500; Potri.008G148200; Potri.008G194400; Potri.010G093400; Potri.012G070900; Potri.015G065400; Potri.017G081700 |
| TFBS_0152 | AT-Hook | 92.1 | 1-18 | Potri.004G189200 |
| TFBS_0129 | AT-Hook | 92.1 | 1-18 | Potri.004G087500; Potri.017G129400 |
| TFBS_0140 | AT-Hook | 92.1 | 1-22 | Potri.002G105500; Potri.005G156100 |
| TFBS_0223 | TCR | 93.1 | 1-17 | Potri.010G083400 |
| TFBS_0227 | TCR | 93.1 | 1-10 | Potri.006G160400; Potri.018G083000 |
| TFBS_0570 | TBP | 93.1 | 1-11 | Potri.013G059300; Potri.019G033300 |
| TFBS_0288 | HD-ZIP | 93.1 | 1-23 | Potri.001G137800; Potri.001G184100; Potri.002G154700; Potri.002G230200; Potri.003G052400; Potri.003G096000; Potri.004G020400; Potri.011G025000; Potri.012G139300; Potri.014G075200; Potri.014G152000; Potri.015G034100; Potri.015G141800; Potri.017G144600 |
| TFBS_0136 | AT-Hook | 93.6 | 1-19 | Potri.002G105500; Potri.005G156100 |
| TFBS_0211 | C2H2 | 94.1 | 1-8 | Potri.001G295500; Potri.009G089400; Potri.014G017300 |
| TFBS_0213 | C2H2 | 94.1 | 1-8 | Potri.001G295500; Potri.009G089400; Potri.014G017300; Potri.002G143800 |
| TFBS_0130 | AT-Hook | 95 | 1-15 | Potri.004G189200 |
| TFBS_0571 | TBP | 97 | 1-14 | Potri.013G059300; Potri.019G033300 |
| TFBS_0569 | TBP | 97.5 | 1-15 | Potri.013G059300; Potri.019G033300 |
| TFBS_0146 | AT-Hook | 98.5 | 1-26 | Potri.002G105500; Potri.005G156100 |
| TFBS_0585 | TBP | 98.5 | 1-16 | Potri.013G059300; Potri.019G033300 |
| TFBS_0148 | AT-Hook | 99 | 1-46 | Potri.004G189200 |
| TFBS_0193 | bZIP | 99 | 1-19 | Potri.002G125400; Potri.004G140600; Potri.006G083000; Potri.009G101200; Potri.010G248300; Potri.014G028200 |
| TFBS_0131 | AT-Hook | 99.5 | 1-29 | Potri.004G189200 |
| ^†^The percentage of TFBS (transcription factor binding sites) in all the candidate promoters. | | | | |

**Table S4.** Details of single nucleotide polymorphisms (SNPs) within all the candidate genes.

| Gene model | *Gene ID* | Total length (bp) | SNP number | Frequency (bp-1) | π | θw |
| --- | --- | --- | --- | --- | --- | --- |
| Potri.018G094200 | *Ptr-4CL2* | 4386 | 92 | 48 | 0.06368 | 0.13136 |
| Potri.001G036900 | *Ptr-4CL3* | 6020 | 92 | 65 | 0.05400 | 0.17879 |
| Potri.019G049500 | *Ptr-4CL4* | 6982 | 96 | 73 | 0.05658 | 0.10550 |
| Potri.003G188500 | *Ptr-4CL5* | 6820 | 190 | 36 | 0.01434 | 0.03777 |
| Potri.001G055700 | *Ptr-4CL6* | 5079 | 69 | 74 | 0.03845 | 0.04331 |
| Potri.005G248500 | *Ptr-4CL7* | 3824 | 21 | 182 | 0.03069 | 0.12299 |
| Potri.003G099700 | *Ptr-4CL9* | 5462 | 130 | 42 | 0.05667 | 0.09627 |
| Potri.004G102000 | *Ptr-4CL11* | 5850 | 78 | 75 | 0.00882 | 0.03324 |
| Potri.017G033600 | *Ptr-4CL12* | 6584 | 146 | 45 | 0.04498 | 0.09357 |
| Potri.010G230200 | *Ptr-4CL13* | 4613 | 111 | 42 | 0.00998 | 0.03233 |
| Potri.010G057000 | *Ptr-4CL14* | 4945 | 47 | 105 | 0.05980 | 0.11625 |
| Potri.012G095000 | *Ptr-4CL17* | 10544 | 172 | 61 | 0.01340 | 0.04511 |
| Potri.006G169700 | *Ptr-4CL20* | 10190 | 135 | 75 | 0.05328 | 0.09930 |
| Potri.008G031500 | *Ptr-4CL21* | 4157 | 38 | 109 | 0.04002 | 0.11435 |
| Potri.016G031100 | *Ptr-C3H2* | 7107 | 134 | 53 | 0.02439 | 0.06303 |
| Potri.006G033300 | *Ptr-C3H3* | 3079 | 30 | 103 | 0.10054 | 0.14980 |
| Potri.001G025200 | *Ptr-C3H4* | 4491 | 82 | 55 | 0.00664 | 0.04275 |
| Potri.019G130700 | *Ptr-C4H2* | 4389 | 56 | 78 | 0.01512 | 0.06840 |
| Potri.018G146100 | *Ptr-C4H3* | 4981 | 127 | 39 | 0.04373 | 0.08433 |
| Potri.006G078100 | *Ptr-C4H4* | 3066 | 58 | 53 | 0.02135 | 0.07160 |
| Potri.009G095800 | *Ptr-CAD1* | 4744 | 55 | 86 | 0.07609 | 0.07491 |
| Potri.016G078300 | *Ptr-CAD2* | 6209 | 87 | 71 | 0.07642 | 0.14877 |
| Potri.009G062800 | *Ptr-CAD5* | 4515 | 114 | 40 | 0.03651 | 0.09881 |
| Potri.009G063400 | *Ptr-CAD6* | 4789 | 130 | 37 | 0.05695 | 0.08378 |
| Potri.001G268600 | *Ptr-CAD7* | 4721 | 56 | 84 | 0.02683 | 0.09130 |
| Potri.006G199100 | *Ptr-CAD8* | 5646 | 22 | 257 | 0.03069 | 0.14843 |
| Potri.001G307200 | *Ptr-CAD10* | 6937 | 215 | 32 | 0.06912 | 0.11788 |
| Potri.002G018300 | *Ptr-CAD12* | 5229 | 31 | 169 | 0.03931 | 0.14527 |
| Potri.003G196700 | *Ptr-CAD13* | 4148 | 99 | 42 | 0.06248 | 0.10697 |
| Potri.016G023300 | *Ptr-CAD14* | 4112 | 93 | 44 | 0.03916 | 0.11393 |
| Potri.006G024300 | *Ptr-CAD16* | 7326 | 108 | 68 | 0.03420 | 0.13410 |
| Potri.005G243700 | *Ptr-CAD18* | 2808 | 44 | 64 | 0.02974 | 0.06205 |
| Potri.006G024400 | *Ptr-CAD19* | 4032 | 34 | 119 | 0.10444 | 0.13389 |
| Potri.011G148100 | *Ptr-CAD22* | 5096 | 22 | 232 | 0.02072 | 0.06874 |
| Potri.009G099800 | *Ptr-CCoAOMT1* | 4115 | 40 | 103 | 0.02493 | 0.10189 |
| Potri.001G304800 | *Ptr-CCoAOMT2* | 4156 | 31 | 134 | 0.08696 | 0.14866 |
| Potri.008G136600 | *Ptr-CCoAOMT3* | 4064 | 95 | 43 | 0.01951 | 0.10208 |
| Potri.010G104400 | *Ptr-CCoAOMT4* | 4867 | 130 | 37 | 0.02978 | 0.08534 |
| Potri.018G070300 | *Ptr-CCoAOMT5* | 4134 | 120 | 34 | 0.08320 | 0.15558 |
| Potri.002G183600 | *Ptr-CCoAOMT6* | 6134 | 111 | 55 | 0.01675 | 0.03389 |
| Potri.003G181400 | *Ptr-CCR2* | 5375 | 148 | 36 | 0.02656 | 0.06913 |
| Potri.004G230900 | *Ptr-CCR8* | 6227 | 91 | 68 | 0.01833 | 0.05643 |
| Potri.009G076300 | *Ptr-CCR9* | 5737 | 139 | 41 | 0.06899 | 0.08031 |
| Potri.002G004100 | *Ptr-CCR10* | 5113 | 43 | 119 | 0.03376 | 0.10011 |
| Potri.002G004500 | *Ptr-CCR11* | 5089 | 38 | 134 | 0.07480 | 0.14678 |
| Potri.001G045100 | *Ptr-CCR13* | 4945 | 42 | 118 | 0.06309 | 0.09294 |
| Potri.001G045400 | *Ptr-CCR15* | 3829 | 85 | 45 | 0.01191 | 0.05805 |
| Potri.001G256400 | *Ptr-CCR23* | 7833 | 159 | 49 | 0.06348 | 0.11087 |
| Potri.005G257700 | *Ptr-CCR25* | 5447 | 58 | 94 | 0.08548 | 0.08702 |
| Potri.008G120200 | *Ptr-CCR26* | 5737 | 193 | 30 | 0.03526 | 0.04311 |
| Potri.009G052000 | *Ptr-CCR28* | 7097 | 140 | 51 | 0.04309 | 0.15602 |
| Potri.009G057500 | *Ptr-CCR29* | 5282 | 135 | 39 | 0.00760 | 0.04913 |
| Potri.009G057600 | *Ptr-CCR30* | 7568 | 177 | 43 | 0.00563 | 0.01736 |
| Potri.010G125400 | *Ptr-CCR33* | 5262 | 59 | 89 | 0.04070 | 0.12016 |
| Potri.015G003100 | *Ptr-COMT1* | 6235 | 46 | 136 | 0.05127 | 0.07127 |
| Potri.012G006400 | *Ptr-COMT2* | 6151 | 54 | 114 | 0.00835 | 0.06611 |
| Potri.014G106600 | *Ptr-COMT3* | 3935 | 46 | 86 | 0.01555 | 0.07537 |
| Potri.002G180600 | *Ptr-COMT4* | 4182 | 39 | 107 | 0.02065 | 0.12734 |
| Potri.003G051700 | *Ptr-COMT6* | 5429 | 125 | 43 | 0.01142 | 0.03441 |
| Potri.006G120000 | *Ptr-COMT7* | 5136 | 30 | 171 | 0.04098 | 0.12196 |
| Potri.011G059500 | *Ptr-COMT8* | 4186 | 100 | 42 | 0.01510 | 0.05135 |
| Potri.004G050400 | *Ptr-COMT9* | 4091 | 59 | 69 | 0.05486 | 0.11360 |
| Potri.004G050500 | *Ptr-COMT10* | 3949 | 55 | 72 | 0.03281 | 0.19865 |
| Potri.011G059600 | *Ptr-COMT11* | 3889 | 85 | 46 | 0.02041 | 0.07991 |
| Potri.019G093000 | *Ptr-COMT12* | 15123 | 122 | 124 | 0.01959 | 0.05910 |
| Potri.013G120800 | *Ptr-COMT13* | 3983 | 46 | 87 | 0.12145 | 0.13290 |
| Potri.009G139800 | *Ptr-COMT15* | 4283 | 45 | 95 | 0.05563 | 0.10866 |
| Potri.013G121800 | *Ptr-COMT16* | 4290 | 78 | 55 | 0.07222 | 0.12764 |
| Potri.013G121400 | *Ptr-COMT18* | 4075 | 61 | 67 | 0.04356 | 0.08019 |
| Potri.013G122500 | *Ptr-COMT19* | 10154 | 209 | 49 | 0.08529 | 0.14878 |
| Potri.013G136400 | *Ptr-COMT22* | 4484 | 132 | 34 | 0.11266 | 0.12301 |
| Potri.013G136300 | *Ptr-COMT23* | 4564 | 74 | 62 | 0.12219 | 0.13433 |
| Potri.019G102900 | *Ptr-COMT24* | 6442 | 76 | 85 | 0.07736 | 0.08915 |
| Potri.001G451100 | *Ptr-COMT25* | 4338 | 173 | 25 | 0.07434 | 0.09334 |
| Potri.002G180500 | *Ptr-COMT27* | 4368 | 46 | 95 | 0.02377 | 0.06679 |
| Potri.005G183800 | *Ptr-COMT28* | 3829 | 80 | 48 | 0.03387 | 0.14875 |
| Potri.009G139700 | *Ptr-COMT29* | 4247 | 87 | 49 | 0.03248 | 0.08725 |
| Potri.011G150500 | *Ptr-COMT30* | 4365 | 61 | 72 | 0.02108 | 0.05756 |
| Potri.014G106500 | *Ptr-COMT34* | 4963 | 109 | 46 | 0.03316 | 0.05752 |
| Potri.019G093100 | *Ptr-COMT38* | 3930 | 56 | 70 | 0.07458 | 0.08895 |
| Potri.001G175000 | *Ptr-CSE1* | 4537 | 56 | 81 | 0.07109 | 0.12701 |
| Potri.003G059200 | *Ptr-CSE2* | 4832 | 53 | 91 | 0.07226 | 0.12879 |
| Potri.005G117500 | *Ptr-F5H1* | 6177 | 87 | 71 | 0.05917 | 0.07480 |
| Potri.007G016400 | *Ptr-F5H2* | 4601 | 60 | 77 | 0.01464 | 0.03643 |
| Potri.009G123600 | *Ptr-F5H3* | 4552 | 85 | 54 | 0.04215 | 0.06566 |
| Potri.004G161600 | *Ptr-F5H4* | 4637 | 81 | 57 | 0.01043 | 0.06120 |
| Potri.003G183900 | *Ptr-HCT1* | 5253 | 94 | 56 | 0.06997 | 0.13362 |
| Potri.018G105500 | *Ptr-HCT2* | 5541 | 64 | 87 | 0.05752 | 0.08990 |
| Potri.001G042900 | *Ptr-HCT6* | 7733 | 56 | 138 | 0.09895 | 0.10843 |
| Potri.005G028000 | *Ptr-HCT7* | 4418 | 45 | 98 | 0.03745 | 0.09031 |
| Potri.004G017600 | *Ptr-HCT8* | 4161 | 100 | 42 | 0.04073 | 0.05916 |
| Potri.006G010300 | *Ptr-HCT11* | 3961 | 77 | 51 | 0.02757 | 0.08316 |
| Potri.006G165200 | *Ptr-HCT12* | 3577 | 90 | 40 | 0.05937 | 0.09344 |
| Potri.007G139400 | *Ptr-HCT13* | 3881 | 72 | 54 | 0.02276 | 0.07024 |
| Potri.008G033500 | *Ptr-HCT14* | 3844 | 65 | 59 | 0.04932 | 0.09296 |
| Potri.008G034100 | *Ptr-HCT15* | 4634 | 117 | 40 | 0.04406 | 0.07704 |
| Potri.008G034200 | *Ptr-HCT16* | 3556 | 121 | 29 | 0.03565 | 0.09638 |
| Potri.008G034300 | *Ptr-HCT17* | 4074 | 61 | 67 | 0.02010 | 0.07795 |
| Potri.018G109900 | *Ptr-HCT19* | 3955 | 102 | 39 | 0.03874 | 0.11693 |
| Potri.019G001200 | *Ptr-HCT20* | 3445 | 14 | 246 | 0.00211 | 0.01532 |
| Potri.019G001400 | *Ptr-HCT21* | 3953 | 6 | 659 | 0.00081 | 0.01322 |
| Potri.001G054600 | *Ptr-LAC1* | 5470 | 53 | 103 | 0.01185 | 0.03286 |
| Potri.001G184300 | *Ptr-LAC2* | 4702 | 140 | 34 | 0.01899 | 0.04622 |
| Potri.001G206200 | *Ptr-LAC3* | 6936 | 109 | 64 | 0.04317 | 0.08356 |
| Potri.001G248700 | *Ptr-LAC4* | 6716 | 75 | 90 | 0.07730 | 0.13243 |
| Potri.001G341600 | *Ptr-LAC5* | 4901 | 225 | 22 | 0.02618 | 0.05474 |
| Potri.001G401300 | *Ptr-LAC7* | 5036 | 96 | 52 | 0.00375 | 0.01395 |
| Potri.004G156400 | *Ptr-LAC8* | 5472 | 30 | 182 | 0.05348 | 0.09910 |
| Potri.005G200600 | *Ptr-LAC9* | 5219 | 136 | 38 | 0.01263 | 0.04517 |
| Potri.005G200700 | *Ptr-LAC10* | 5015 | 116 | 43 | 0.05085 | 0.09878 |
| Potri.006G094100 | *Ptr-LAC13* | 5765 | 59 | 98 | 0.01144 | 0.03421 |
| Potri.007G023300 | *Ptr-LAC16* | 4765 | 96 | 50 | 0.01242 | 0.04717 |
| Potri.008G064000 | *Ptr-LAC17* | 5381 | 99 | 54 | 0.02086 | 0.04212 |
| Potri.008G073800 | *Ptr-LAC19* | 5705 | 64 | 89 | 0.03377 | 0.06053 |
| Potri.009G042500 | *Ptr-LAC21* | 4929 | 35 | 141 | 0.07587 | 0.10574 |
| Potri.009G102700 | *Ptr-LAC22* | 4995 | 117 | 43 | 0.00886 | 0.02384 |
| Potri.010G183500 | *Ptr-LAC25* | 5894 | 54 | 109 | 0.05123 | 0.09835 |
| Potri.010G183600 | *Ptr-LAC26* | 5561 | 121 | 46 | 0.03800 | 0.08712 |
| Potri.010G193100 | *Ptr-LAC27* | 6202 | 57 | 109 | 0.02780 | 0.12375 |
| Potri.011G071100 | *Ptr-LAC28* | 5768 | 107 | 54 | 0.07652 | 0.10850 |
| Potri.011G120300 | *Ptr-LAC30* | 4984 | 69 | 72 | 0.05282 | 0.13920 |
| Potri.012G048900 | *Ptr-LAC31* | 4745 | 59 | 80 | 0.04181 | 0.13431 |
| Potri.013G152700 | *Ptr-LAC32* | 5316 | 115 | 46 | 0.01743 | 0.04681 |
| Potri.014G100600 | *Ptr-LAC33* | 5176 | 56 | 92 | 0.02020 | 0.06270 |
| Potri.015G040600 | *Ptr-LAC35* | 4585 | 66 | 69 | 0.04755 | 0.09133 |
| Potri.015G040400 | *Ptr-LAC36* | 4623 | 46 | 101 | 0.01857 | 0.08904 |
| Potri.016G106000 | *Ptr-LAC37* | 4451 | 138 | 32 | 0.01672 | 0.02405 |
| Potri.016G112000 | *Ptr-LAC40* | 5677 | 153 | 37 | 0.07201 | 0.09007 |
| Potri.019G088500 | *Ptr-LAC43* | 5600 | 46 | 122 | 0.03354 | 0.06532 |
| Potri.019G088600 | *Ptr-LAC44* | 4932 | 46 | 107 | 0.00304 | 0.02010 |
| Potri.006G126800 | *Ptr-PAL1* | 5984 | 67 | 89 | 0.02683 | 0.07168 |
| Potri.008G038200 | *Ptr-PAL2* | 5687 | 70 | 81 | 0.11077 | 0.09531 |
| Potri.016G091100 | *Ptr-PAL3* | 5860 | 126 | 47 | 0.04441 | 0.09120 |
| Potri.010G224100 | *Ptr-PAL4* | 5729 | 65 | 88 | 0.02731 | 0.12871 |
| Potri.010G224200 | *Ptr-PAL5* | 6034 | 139 | 43 | 0.04048 | 0.11517 |
| Potri.001G011500 | *Ptr-PO5* | 4192 | 68 | 62 | 0.02340 | 0.03996 |
| Potri.001G145800 | *Ptr-PO9* | 5183 | 25 | 207 | 0.05087 | 0.10998 |
| Potri.001G329200 | *Ptr-PO11* | 4650 | 56 | 83 | 0.05714 | 0.13403 |
| Potri.001G458700 | *Ptr-PO13* | 3770 | 15 | 251 | 0.00833 | 0.04321 |
| Potri.002G031200 | *Ptr-PO15* | 4415 | 22 | 201 | 0.08428 | 0.16957 |
| Potri.002G065300 | *Ptr-PO16* | 4786 | 27 | 177 | 0.07239 | 0.16771 |
| Potri.003G053700 | *Ptr-PO17* | 4457 | 63 | 71 | 0.08816 | 0.12592 |
| Potri.003G156100 | *Ptr-PO18* | 4162 | 101 | 41 | 0.01081 | 0.03273 |
| Potri.003G214500 | *Ptr-PO19* | 4347 | 89 | 49 | 0.02939 | 0.02570 |
| Potri.003G214700 | *Ptr-PO21* | 4322 | 103 | 42 | 0.02754 | 0.03737 |
| Potri.003G214800 | *Ptr-PO22* | 4098 | 64 | 64 | 0.07638 | 0.09724 |
| Potri.003G214900 | *Ptr-PO23* | 4335 | 103 | 42 | 0.07913 | 0.10728 |
| Potri.004G006400 | *Ptr-PO24* | 3389 | 23 | 147 | 0.03709 | 0.07543 |
| Potri.004G015300 | *Ptr-PO25* | 4530 | 82 | 55 | 0.02059 | 0.07731 |
| Potri.004G023200 | *Ptr-PO26* | 3857 | 73 | 53 | 0.02092 | 0.03522 |
| Potri.004G052100 | *Ptr-PO27* | 4637 | 52 | 89 | 0.03433 | 0.07299 |
| Potri.004G134800 | *Ptr-PO28* | 4149 | 82 | 51 | 0.02149 | 0.07569 |
| Potri.004G144600 | *Ptr-PO30* | 4763 | 42 | 113 | 0.03594 | 0.07745 |
| Potri.005G072800 | *Ptr-PO31* | 4705 | 87 | 54 | 0.03716 | 0.09280 |
| Potri.005G108900 | *Ptr-PO33* | 3788 | 62 | 61 | 0.01449 | 0.05788 |
| Potri.005G118700 | *Ptr-PO34* | 3990 | 63 | 63 | 0.01646 | 0.05340 |
| Potri.005G135300 | *Ptr-PO35* | 4129 | 81 | 51 | 0.01208 | 0.06211 |
| Potri.005G195600 | *Ptr-PO36* | 5196 | 71 | 73 | 0.02992 | 0.10842 |
| Potri.005G195700 | *Ptr-PO37* | 4954 | 93 | 53 | 0.04717 | 0.10221 |
| Potri.006G069600 | *Ptr-PO38* | 3942 | 56 | 70 | 0.02158 | 0.03513 |
| Potri.006G107000 | *Ptr-PO39* | 4162 | 91 | 46 | 0.04231 | 0.11830 |
| Potri.006G129900 | *Ptr-PO40* | 4405 | 146 | 30 | 0.03745 | 0.09974 |
| Potri.006G267400 | *Ptr-PO41* | 4269 | 61 | 70 | 0.07104 | 0.13136 |
| Potri.007G019300 | *Ptr-PO42* | 3763 | 74 | 51 | 0.04182 | 0.04349 |
| Potri.007G067200 | *Ptr-PO43* | 5386 | 77 | 70 | 0.03082 | 0.06904 |
| Potri.007G074600 | *Ptr-PO44* | 3546 | 36 | 99 | 0.11152 | 0.13643 |
| Potri.007G074700 | *Ptr-PO45* | 3691 | 43 | 86 | 0.02397 | 0.10086 |
| Potri.007G096200 | *Ptr-PO46* | 4629 | 51 | 91 | 0.00837 | 0.05207 |
| Potri.007G122100 | *Ptr-PO48* | 4067 | 24 | 169 | 0.08833 | 0.07315 |
| Potri.007G122200 | *Ptr-PO49* | 3804 | 51 | 75 | 0.01422 | 0.06481 |
| Potri.008G022600 | *Ptr-PO51* | 3654 | 75 | 49 | 0.05957 | 0.10466 |
| Potri.008G022700 | *Ptr-PO52* | 4004 | 42 | 95 | 0.04330 | 0.06800 |
| Potri.008G103200 | *Ptr-PO53* | 3783 | 106 | 36 | 0.05468 | 0.08157 |
| Potri.008G106400 | *Ptr-PO54* | 6673 | 73 | 91 | 0.01512 | 0.06119 |
| Potri.008G110600 | *Ptr-PO55* | 4120 | 88 | 47 | 0.05279 | 0.07639 |
| Potri.010G036100 | *Ptr-PO57* | 3496 | 62 | 56 | 0.05192 | 0.08961 |
| Potri.010G134500 | *Ptr-PO58* | 3953 | 60 | 66 | 0.01277 | 0.03738 |
| Potri.010G175100 | *Ptr-PO61* | 3894 | 70 | 56 | 0.01595 | 0.06603 |
| Potri.010G236900 | *Ptr-PO62* | 3965 | 133 | 30 | 0.02423 | 0.08053 |
| Potri.011G027300 | *Ptr-PO63* | 4106 | 47 | 87 | 0.07958 | 0.12929 |
| Potri.011G062300 | *Ptr-PO64* | 4404 | 136 | 32 | 0.01829 | 0.02718 |
| Potri.012G006800 | *Ptr-PO65* | 4073 | 115 | 35 | 0.00894 | 0.03959 |
| Potri.012G042800 | *Ptr-PO66* | 4106 | 88 | 47 | 0.03055 | 0.11779 |
| Potri.012G076500 | *Ptr-PO67* | 3487 | 66 | 53 | 0.04351 | 0.06623 |
| Potri.013G066800 | *Ptr-PO68* | 3761 | 75 | 50 | 0.00736 | 0.03641 |
| Potri.013G083600 | *Ptr-PO69* | 4812 | 68 | 71 | 0.05476 | 0.11509 |
| Potri.013G154400 | *Ptr-PO70* | 4086 | 130 | 31 | 0.04975 | 0.09102 |
| Potri.013G156500 | *Ptr-PO72* | 5206 | 83 | 63 | 0.05143 | 0.05430 |
| Potri.013G156800 | *Ptr-PO73* | 3689 | 80 | 46 | 0.06676 | 0.04898 |
| Potri.014G143200 | *Ptr-PO74* | 4099 | 33 | 124 | 0.03883 | 0.06546 |
| Potri.015G003500 | *Ptr-PO75* | 3900 | 24 | 163 | 0.09415 | 0.16083 |
| Potri.015G003600 | *Ptr-PO76* | 3892 | 30 | 130 | 0.05645 | 0.15964 |
| Potri.016G058200 | *Ptr-PO78* | 4244 | 51 | 83 | 0.04531 | 0.05571 |
| Potri.016G058800 | *Ptr-PO79* | 4208 | 25 | 168 | 0.03988 | 0.14862 |
| Potri.016G132800 | *Ptr-PO82* | 3974 | 103 | 39 | 0.06279 | 0.06562 |
| Potri.016G132900 | *Ptr-PO83* | 3900 | 55 | 71 | 0.04439 | 0.14548 |
| Potri.017G037900 | *Ptr-PO84* | 4038 | 81 | 50 | 0.04249 | 0.09527 |
| Potri.017G038000 | *Ptr-PO85* | 4164 | 92 | 45 | 0.09902 | 0.13604 |
| Potri.018G015500 | *Ptr-PO87* | 4815 | 61 | 79 | 0.04288 | 0.08579 |
| Potri.018G089900 | *Ptr-PO88* | 4644 | 63 | 74 | 0.07078 | 0.14630 |
| Potri.018G131600 | *Ptr-PO89* | 3412 | 53 | 64 | 0.06130 | 0.18297 |
| Potri.018G136900 | *Ptr-PO90* | 5637 | 64 | 88 | 0.11508 | 0.08335 |
| Potri.019G063200 | *Ptr-PO91* | 3826 | 63 | 61 | 0.03902 | 0.17640 |
| Potri.002G105500 | *Ptr-AT-Hook3* | 5132 | 65 | 79 | 0.07134 | 0.12994 |
| Potri.004G087500 | *Ptr-AT-Hook1* | 7407 | 59 | 126 | 0.05038 | 0.09752 |
| Potri.004G189200 | *Ptr-AT-Hook2* | 4658 | 124 | 38 | 0.04541 | 0.09692 |
| Potri.005G156100 | *Ptr-AT-Hook5* | 5878 | 86 | 68 | 0.04499 | 0.08968 |
| Potri.017G129400 | *Ptr-AT-Hook4* | 4673 | 90 | 52 | 0.03538 | 0.07012 |
| Potri.005G121900 | *Ptr-bHLH6* | 4269 | 136 | 31 | 0.03281 | 0.06390 |
| Potri.008G190800 | *Ptr-bHLH13* | 5206 | 105 | 50 | 0.02477 | 0.07345 |
| Potri.002G248500 | *Ptr-bHLH2* | 5627 | 20 | 281 | 0.03913 | 0.11882 |
| Potri.003G164500 | *Ptr-bHLH3* | 3716 | 10 | 372 | 0.01626 | 0.11377 |
| Potri.004G099400 | *Ptr-bHLH4* | 4729 | 62 | 76 | 0.07561 | 0.13047 |
| Potri.004G156000 | *Ptr-bHLH5* | 5484 | 70 | 78 | 0.04648 | 0.09612 |
| Potri.005G146500 | *Ptr-bHLH8* | 6193 | 55 | 113 | 0.01722 | 0.06901 |
| Potri.006G057200 | *Ptr-bHLH9* | 7827 | 122 | 64 | 0.01020 | 0.04335 |
| Potri.006G135600 | *Ptr-bHLH10* | 8203 | 55 | 149 | 0.05518 | 0.15895 |
| Potri.007G023600 | *Ptr-bHLH11* | 5766 | 56 | 103 | 0.05687 | 0.12794 |
| Potri.010G136100 | *Ptr-bHLH16* | 7001 | 87 | 80 | 0.01968 | 0.05414 |
| Potri.008G113200 | *Ptr-bHLH12* | 6577 | 47 | 140 | 0.01762 | 0.05580 |
| Potri.009G117300 | *Ptr-bHLH14* | 5580 | 52 | 107 | 0.01071 | 0.04587 |
| Potri.010G040000 | *Ptr-bHLH15* | 5663 | 89 | 64 | 0.02156 | 0.06340 |
| Potri.012G055700 | *Ptr-bHLH17* | 4393 | 39 | 113 | 0.07620 | 0.17186 |
| Potri.012G072700 | *Ptr-bHLH18* | 5707 | 116 | 49 | 0.04260 | 0.10361 |
| Potri.012G104900 | *Ptr-bHLH19* | 4533 | 27 | 168 | 0.01641 | 0.08232 |
| Potri.014G148900 | *Ptr-bHLH20* | 5789 | 53 | 109 | 0.04710 | 0.10152 |
| Potri.015G068100 | *Ptr-bHLH21* | 6015 | 71 | 85 | 0.08712 | 0.08845 |
| Potri.015G104200 | *Ptr-bHLH22* | 5375 | 43 | 125 | 0.02839 | 0.07594 |
| Potri.017G115300 | *Ptr-bHLH7* | 5209 | 73 | 71 | 0.00975 | 0.03610 |
| Potri.002G235400 | *Ptr-bHLH1* | 5760 | 88 | 65 | 0.01028 | 0.04719 |
| Potri.018G109500 | *Ptr-bHLH23* | 6417 | 103 | 62 | 0.03151 | 0.07735 |
| Potri.002G125400 | *Ptr-bZIP1* | 7202 | 112 | 64 | 0.01295 | 0.04250 |
| Potri.004G140600 | *Ptr-bZIP2* | 6885 | 148 | 47 | 0.01800 | 0.04758 |
| Potri.006G083000 | *Ptr-bZIP3* | 6603 | 53 | 125 | 0.01230 | 0.07068 |
| Potri.009G101200 | *Ptr-bZIP4* | 4836 | 136 | 36 | 0.03818 | 0.07159 |
| Potri.010G248300 | *Ptr-bZIP5* | 6327 | 142 | 45 | 0.03887 | 0.08095 |
| Potri.014G028200 | *Ptr-bZIP6* | 6412 | 23 | 279 | 0.03353 | 0.12121 |
| Potri.001G295500 | *Ptr-C2H2-2* | 3615 | 12 | 301 | 0.03789 | 0.05421 |
| Potri.009G089400 | *Ptr-C2H2-33* | 3769 | 25 | 151 | 0.06467 | 0.11966 |
| Potri.014G017300 | *Ptr-C2H2-72* | 3563 | 104 | 34 | 0.03461 | 0.07907 |
| Potri.002G143800 | *Ptr-C2H2-77* | 3619 | 83 | 44 | 0.03291 | 0.07797 |
| Potri.001G137800 | *Ptr-HD-ZIP1* | 8449 | 145 | 58 | 0.01718 | 0.05439 |
| Potri.001G184100 | *Ptr-HD-ZIP4* | 7809 | 126 | 62 | 0.02401 | 0.04901 |
| Potri.001G229700 | *Ptr-HD-ZIP6* | 4535 | 48 | 94 | 0.02693 | 0.06857 |
| Potri.002G100600 | *Ptr-HD-ZIP10* | 4365 | 81 | 54 | 0.04949 | 0.13907 |
| Potri.002G113400 | *Ptr-HD-ZIP11* | 4094 | 25 | 164 | 0.03750 | 0.05311 |
| Potri.002G136400 | *Ptr-HD-ZIP12* | 5379 | 75 | 72 | 0.01810 | 0.06371 |
| Potri.002G154700 | *Ptr-HD-ZIP13* | 8748 | 128 | 68 | 0.05653 | 0.10531 |
| Potri.002G230200 | *Ptr-HD-ZIP15* | 7976 | 73 | 109 | 0.02544 | 0.03622 |
| Potri.003G052400 | *Ptr-HD-ZIP17* | 8006 | 156 | 51 | 0.03803 | 0.06241 |
| Potri.003G079800 | *Ptr-HD-ZIP18* | 5082 | 72 | 71 | 0.05100 | 0.13959 |
| Potri.003G096000 | *Ptr-HD-ZIP19* | 8507 | 54 | 158 | 0.04055 | 0.06047 |
| Potri.004G020400 | *Ptr-HD-ZIP21* | 7356 | 92 | 80 | 0.02304 | 0.04699 |
| Potri.015G034100 | *Ptr-HD-ZIP54* | 6522 | 68 | 96 | 0.03904 | 0.08408 |
| Potri.005G071900 | *Ptr-HD-ZIP24* | 6116 | 113 | 54 | 0.02091 | 0.10967 |
| Potri.005G147100 | *Ptr-HD-ZIP27* | 3821 | 95 | 40 | 0.08212 | 0.13961 |
| Potri.005G161500 | *Ptr-HD-ZIP26* | 4250 | 36 | 118 | 0.04753 | 0.19074 |
| Potri.007G008200 | *Ptr-HD-ZIP35* | 4125 | 76 | 54 | 0.03615 | 0.09529 |
| Potri.008G129500 | *Ptr-HD-ZIP36* | 5813 | 99 | 59 | 0.01358 | 0.05399 |
| Potri.008G148200 | *Ptr-HD-ZIP37* | 5125 | 91 | 56 | 0.00907 | 0.04597 |
| Potri.008G194400 | *Ptr-HD-ZIP38* | 4666 | 72 | 65 | 0.01191 | 0.05082 |
| Potri.009G023600 | *Ptr-HD-ZIP40* | 4607 | 38 | 121 | 0.01509 | 0.05902 |
| Potri.010G093400 | *Ptr-HD-ZIP41* | 4932 | 120 | 41 | 0.01326 | 0.05607 |
| Potri.011G025000 | *Ptr-HD-ZIP43* | 7950 | 84 | 95 | 0.04287 | 0.13207 |
| Potri.012G070900 | *Ptr-HD-ZIP48* | 5086 | 102 | 50 | 0.02004 | 0.06416 |
| Potri.012G139300 | *Ptr-HD-ZIP49* | 6696 | 115 | 58 | 0.03965 | 0.11403 |
| Potri.014G045100 | *Ptr-HD-ZIP50* | 4993 | 21 | 238 | 0.05708 | 0.13688 |
| Potri.014G075200 | *Ptr-HD-ZIP51* | 9316 | 113 | 82 | 0.04912 | 0.07595 |
| Potri.014G152000 | *Ptr-HD-ZIP53* | 8364 | 106 | 79 | 0.02839 | 0.06777 |
| Potri.015G065400 | *Ptr-HD-ZIP55* | 5028 | 36 | 140 | 0.07842 | 0.11647 |
| Potri.015G141800 | *Ptr-HD-ZIP56* | 7388 | 234 | 32 | 0.06481 | 0.10064 |
| Potri.016G059000 | *Ptr-HD-ZIP57* | 4803 | 53 | 91 | 0.03227 | 0.11711 |
| Potri.006G193700 | *Ptr-HD-ZIP30* | 5199 | 55 | 95 | 0.03551 | 0.11684 |
| Potri.017G081700 | *Ptr-HD-ZIP60* | 4876 | 35 | 139 | 0.04285 | 0.16315 |
| Potri.017G144600 | *Ptr-HD-ZIP58* | 6561 | 86 | 76 | 0.04516 | 0.10004 |
| Potri.006G071600 | *Ptr-HD-ZIP28* | 4100 | 87 | 47 | 0.08067 | 0.14627 |
| Potri.004G074300 | *Ptr-Myb/SANT1* | 6416 | 19 | 338 | 0.04447 | 0.12812 |
| Potri.012G038300 | *Ptr-Myb/SANT2* | 7156 | 99 | 72 | 0.02227 | 0.04939 |
| Potri.015G030400 | *Ptr-Myb/SANT3* | 6102 | 82 | 74 | 0.03198 | 0.07046 |
| Potri.013G059300 | *Ptr-TBP1* | 7560 | 21 | 360 | 0.09756 | 0.13963 |
| Potri.019G033300 | *Ptr-TBP2* | 7874 | 164 | 48 | 0.01657 | 0.04809 |
| Potri.006G160400 | *Ptr-TCR2* | 10262 | 74 | 139 | 0.03462 | 0.06798 |
| Potri.010G083400 | *Ptr-TCR1* | 7822 | 116 | 67 | 0.01093 | 0.02550 |
| Potri.018G083000 | *Ptr-TCR3* | 11777 | 38 | 310 | 0.07530 | 0.17255 |
| Pto-miR160e | *MiR160e* | 1300 | 17 | 76 | 0.11282 | 0.20393 |
| Pto-miR167a | *MiR167a* | 1289 | 45 | 29 | 0.09542 | 0.12579 |
| Pto-miR167b | *MiR167b* | 1296 | 44 | 29 | 0.01743 | 0.05532 |
| Pto-miR167c | *MiR167c* | 1289 | 19 | 68 | 0.00268 | 0.02320 |
| Pto-miR167d | *MiR167d* | 1305 | 53 | 25 | 0.02093 | 0.09372 |
| Pto-miR167e | *MiR167e* | 1291 | 5 | 258 | 0.00048 | 0.04354 |
| Pto-miR167f | *MiR167f* | 1288 | 28 | 46 | 0.02532 | 0.07745 |
| Pto-miR167g | *MiR167g* | 1286 | 10 | 129 | 0.08000 | 0.17704 |
| Pto-miR167h | *MiR167h* | 1291 | 27 | 48 | 0.00364 | 0.01600 |
| Pto-miR172e | *MiR172e* | 1329 | 17 | 78 | 0.02378 | 0.11716 |
| Pto-miR172g | *MiR172g* | 1343 | 18 | 75 | 0.01140 | 0.06320 |
| Pto-miR172h | *MiR172h* | 1343 | 12 | 112 | 0.02964 | 0.05532 |
| Pto-miR396g | *MiR396g* | 1371 | 24 | 57 | 0.01986 | 0.06486 |
| Pto-miR397a | *MiR397a* | 1320 | 24 | 55 | 0.01177 | 0.05778 |
| Pto-miR397b | *MiR397b* | 1309 | 18 | 73 | 0.02303 | 0.05936 |
| Pto-miR408 | *MiR408* | 1305 | 18 | 73 | 0.03747 | 0.08534 |
| Pto-miR475a | *MiR475a* | 1324 | 17 | 78 | 0.03763 | 0.10071 |
| Pto-miR475b | *MiR475b* | 1336 | 10 | 134 | 0.01141 | 0.06557 |
| Pto-miR475c | *MiR475c* | 1325 | 26 | 51 | 0.01487 | 0.05085 |
| Pto-miR475d | *MiR475d* | 1303 | 31 | 42 | 0.00266 | 0.04227 |
| Pto-miR482c | *MiR482c* | 1371 | 12 | 114 | 0.12435 | 0.17684 |
| Pto-miR6425a | *MiR6425a* | 1420 | 15 | 95 | 0.01036 | 0.04574 |
| Pto-miR6425b | *MiR6425b* | 1420 | 22 | 65 | 0.08009 | 0.05097 |
| Pto-miR6425c | *MiR6425c* | 1420 | 15 | 95 | 0.01036 | 0.04574 |
| Pto-miR6425d | *MiR6425d* | 1420 | 22 | 65 | 0.08009 | 0.05097 |
| Pto-miR6425e | *MiR6425e* | 1441 | 15 | 96 | 0.01036 | 0.04574 |
| Pto-miR6427 | *MiR6427* | 1304 | 24 | 54 | 0.08173 | 0.16688 |
| Pto-miR6439a | *MiR6439a* | 1463 | 23 | 64 | 0.00172 | 0.00950 |
| Pto-miR6443 | *MiR6443* | 1318 | 23 | 57 | 0.00767 | 0.02010 |
| Pto-miR6446 | *MiR6446* | 1423 | 94 | 15 | 0.02617 | 0.12749 |
| Pto-miR6452 | *MiR6452* | 1318 | 39 | 34 | 0.03837 | 0.01135 |
| Pto-miR6456 | *MiR6456* | 1292 | 22 | 59 | 0.02425 | 0.06010 |
| Pto-miR394a | *MiR394a* | 1348 | 6 | 225 | 0.03919 | 0.18777 |
| Pto-miR394b | *MiR394b* | 1347 | 29 | 46 | 0.00863 | 0.06005 |
| Pto-miR6462c | *MiR6462c* | 1333 | 25 | 53 | 0.13025 | 0.14023 |
| Pto-miR482d | *MiR482d* | 1370 | 35 | 39 | 0.02553 | 0.13585 |
| L01 | *LncRNA locus 1* | 3597 | 68 | 53 | 0.02266 | 0.06959 |
| L02 | *LncRNA locus 2* | 4291 | 78 | 55 | 0.09366 | 0.12233 |
| L03 | *LncRNA locus 3* | 6397 | 67 | 95 | 0.11978 | 0.12672 |
| L04 | *LncRNA locus 4* | 5196 | 77 | 67 | 0.09647 | 0.14083 |
| L05 | *LncRNA locus 5* | 6748 | 89 | 76 | 0.00945 | 0.04476 |
| L06 | *LncRNA locus 6* | 18527 | 159 | 117 | 0.06255 | 0.13708 |
| L07 | *LncRNA locus 7* | 4027 | 89 | 45 | 0.01470 | 0.04857 |
| L08 | *LncRNA locus 8* | 3887 | 114 | 34 | 0.02081 | 0.04369 |
| L09 | *LncRNA locus 9* | 6773 | 65 | 104 | 0.07455 | 0.12365 |
| L10 | *LncRNA locus 10* | 10629 | 123 | 86 | 0.04355 | 0.06837 |
| L11 | *LncRNA locus 11* | 3234 | 21 | 154 | 0.01687 | 0.04272 |
| L12 | *LncRNA locus 12* | 3180 | 27 | 118 | 0.07239 | 0.16771 |
| L13 | *LncRNA locus 13* | 12222 | 213 | 57 | 0.04407 | 0.11068 |
| L14 | *LncRNA locus 14* | 4826 | 49 | 98 | 0.01497 | 0.02914 |
| L15 | *LncRNA locus 15* | 4845 | 70 | 69 | 0.06046 | 0.15773 |
| L16 | *LncRNA locus 16* | 4206 | 96 | 44 | 0.04494 | 0.09865 |
| L17 | *LncRNA locus 17* | 12932 | 240 | 54 | 0.03952 | 0.08184 |
| L18 | *LncRNA locus 18* | 10583 | 179 | 59 | 0.00953 | 0.03099 |
| L19 | *LncRNA locus 19* | 12302 | 222 | 55 | 0.05219 | 0.10048 |
| L20 | *LncRNA locus 20* | 3909 | 109 | 36 | 0.03190 | 0.07613 |
| L21 | *LncRNA locus 21* | 3692 | 39 | 95 | 0.01331 | 0.07442 |
| L22 | *LncRNA locus 22* | 3395 | 24 | 141 | 0.02622 | 0.10482 |
| L23 | *LncRNA locus 23* | 11919 | 50 | 238 | 0.04293 | 0.15425 |
| L24 | *LncRNA locus 24* | 3966 | 40 | 99 | 0.04920 | 0.06884 |
| L25 | *LncRNA locus 25* | 5917 | 39 | 152 | 0.05220 | 0.15629 |
| L26 | *LncRNA locus 26* | 4639 | 72 | 64 | 0.02118 | 0.10388 |
| L27 | *LncRNA locus 27* | 3587 | 123 | 29 | 0.03755 | 0.12896 |
| L28 | *LncRNA locus 28* | 8374 | 106 | 79 | 0.01829 | 0.07120 |
| L29 | *LncRNA locus 29* | 9209 | 83 | 111 | 0.02003 | 0.10411 |
| L30 | *LncRNA locus 30* | 3816 | 112 | 34 | 0.04598 | 0.11113 |
| L31 | *LncRNA locus 31* | 3994 | 55 | 73 | 0.08876 | 0.10384 |
| L32 | *LncRNA locus 32* | 5773 | 116 | 50 | 0.03324 | 0.07121 |
| L33 | *LncRNA locus 33* | 4129 | 67 | 62 | 0.09211 | 0.13528 |
| L34 | *LncRNA locus 34* | 2793 | 74 | 38 | 0.03024 | 0.05612 |
| L35 | *LncRNA locus 35* | 7434 | 158 | 47 | 0.02072 | 0.05205 |
| L36 | *LncRNA locus 36* | 5279 | 83 | 64 | 0.00151 | 0.01345 |
| L37 | *LncRNA locus 37* | 5199 | 43 | 121 | 0.03301 | 0.09896 |
| L38 | *LncRNA locus 38* | 7531 | 91 | 83 | 0.04392 | 0.04171 |
| L39 | *LncRNA locus 39* | 5137 | 98 | 52 | 0.04002 | 0.06774 |
| L40 | *LncRNA locus 40* | 8107 | 305 | 27 | 0.02680 | 0.03716 |
| L41 | *LncRNA locus 41* | 10118 | 168 | 60 | 0.01794 | 0.06379 |
| L42 | *LncRNA locus 42* | 3197 | 68 | 47 | 0.01065 | 0.04195 |
| L43 | *LncRNA locus 43* | 5333 | 100 | 53 | 0.03643 | 0.04957 |
| L44 | *LncRNA locus 44* | 6421 | 68 | 94 | 0.07822 | 0.13748 |
| L45 | *LncRNA locus 45* | 6662 | 78 | 85 | 0.10603 | 0.10590 |
| L46 | *LncRNA locus 46* | 6285 | 65 | 97 | 0.02667 | 0.06268 |
| L47 | *LncRNA locus 47* | 10869 | 121 | 90 | 0.03268 | 0.04917 |
| L48 | *LncRNA locus 48* | 4426 | 109 | 41 | 0.03953 | 0.08522 |
| L49 | *LncRNA locus 49* | 4642 | 105 | 44 | 0.02492 | 0.09414 |
| L50 | *LncRNA locus 50* | 11979 | 246 | 49 | 0.05091 | 0.09674 |
| L51 | *LncRNA locus 51* | 6882 | 78 | 88 | 0.03237 | 0.07535 |
| L52 | *LncRNA locus 52* | 5851 | 86 | 68 | 0.06221 | 0.05971 |
| L53 | *LncRNA locus 53* | 6609 | 52 | 127 | 0.03689 | 0.03111 |
| L54 | *LncRNA locus 54* | 5106 | 35 | 146 | 0.05909 | 0.11052 |
| L55 | *LncRNA locus 55* | 8463 | 79 | 107 | 0.04832 | 0.07686 |
| L56 | *LncRNA locus 56* | 6744 | 88 | 77 | 0.04523 | 0.05237 |
| L57 | *LncRNA locus 57* | 4393 | 105 | 42 | 0.01441 | 0.03722 |
| L58 | *LncRNA locus 58* | 4172 | 61 | 68 | 0.05354 | 0.10433 |
| L59 | *LncRNA locus 59* | 3677 | 55 | 67 | 0.02398 | 0.06495 |
| L60 | *LncRNA locus 60* | 19234 | 243 | 79 | 0.01563 | 0.06503 |
| L61 | *LncRNA locus 61* | 2815 | 18 | 156 | 0.03146 | 0.10272 |
| L62 | *LncRNA locus 62* | 7979 | 135 | 59 | 0.01967 | 0.05251 |
| L63 | *LncRNA locus 63* | 3429 | 46 | 75 | 0.04503 | 0.08144 |
| L64 | *LncRNA locus 64* | 2984 | 27 | 111 | 0.01648 | 0.01632 |
| L65 | *LncRNA locus 65* | 4915 | 45 | 109 | 0.00801 | 0.03826 |
| L66 | *LncRNA locus 66* | 8046 | 46 | 175 | 0.09308 | 0.12961 |
| L67 | *LncRNA locus 67* | 6775 | 132 | 51 | 0.03630 | 0.09895 |
| L68 | *LncRNA locus 68* | 6912 | 61 | 113 | 0.07467 | 0.15145 |
| L69 | *LncRNA locus 69* | 2730 | 9 | 303 | 0.08534 | 0.10388 |
| L70 | *LncRNA locus 70* | 6064 | 55 | 110 | 0.06509 | 0.12914 |
| L71 | *LncRNA locus 71* | 3429 | 50 | 69 | 0.00747 | 0.04848 |

**Table S5.** Details of significant SNPs within candidate genes associated with growth and wood properties in the association population of *P. tomentosa*.

| **Traits** | **Associated SNP** | **Category** | **Region** | **Additive effect** | **Dominant effect** | ***P*-value** | ***R^2^*(%)** |
| --- | --- | --- | --- | --- | --- | --- | --- |
| DBH | Ptr-HD-ZIP54_SNP 54 | TF gene | Promoter | 4.694 | -1.704 | 1.08E-11 | 21.06 |
| FL | Ptr-4CL12_SNP28 | Lignin biosynthesis gene | Promoter | 0.013 | -0.049 | 1.21E-10 | 26.44 |
| FL | Ptr-PO25_SNP36 | Lignin biosynthesis gene | Promoter |  | -0.010 | 3.73E-10 | 26.73 |
| FL | Ptr-PO25_SNP37 | Lignin biosynthesis gene | Promoter | 0.007 |  | 5.88E-10 | 26.27 |
| FL | Ptr-PO25_SNP38 | Lignin biosynthesis gene | Promoter | 0.087 |  | 5.88E-10 | 26.27 |
| FL | Ptr-TBP2_SNP 156 | TF gene | Promoter | 0.008 |  | 1.32E-09 | 26.67 |
| FL | Ptr-COMT24_SNP31 | Lignin biosynthesis gene | CDS | 0.097 |  | 1.06E-08 | 24.20 |
| DBH | Pto-MIR167d_SNP 11 | MiRNA gene | Flanking | 2.595 | -2.388 | 1.72E-08 | 26.85 |
| FL | Ptr-bHLH5_SNP 10 | TF gene | Promoter | 0.096 | 0.049 | 4.52E-08 | 27.36 |
| HC | Ptr-PO43_SNP9 | Lignin biosynthesis gene | Promoter | 12.149 | 18.424 | 1.09E-07 | 31.30 |
| DBH | Pto-MIR167a_SNP 23 | MiRNA gene | Flanking | 3.152 | -1.427 | 4.38E-07 | 20.59 |
| FL | Ptr-F5H3_SNP28 | Lignin biosynthesis gene | Promoter | 0.099 |  | 9.04E-07 | 20.49 |
| DBH | Ptr-CSE1_SNP41 | Lignin biosynthesis gene | Intron | 5.514 | 0.813 | 1.06E-06 | 23.64 |
| FW | L17_SNP 65 | LncRNA loci | LncRNA coding region | 1.114 |  | 3.01E-06 | 23.32 |
| FW | Ptr-4CL9_SNP39 | Lignin biosynthesis gene | Intron | 1.114 |  | 3.01E-06 | 23.32 |
| DBH | Pto-MIR397a_SNP 7 | MiRNA gene | Pre-miRNA | 6.304 |  | 5.27E-06 | 22.56 |
| FW | Ptr-PO54_SNP44 | Lignin biosynthesis gene | Intron |  | -1.117 | 5.68E-06 | 23.86 |
| FW | Ptr-Myb/SANT2_SNP 99 | TF gene | Promoter | 1.579 |  | 6.04E-06 | 22.02 |
| FL | Ptr-bHLH5_SNP 14 | TF gene | Promoter | 0.088 |  | 8.81E-06 | 18.54 |
| DBH | Pto-MIR167c_SNP 18 | MiRNA gene | Flanking | 3.972 |  | 9.39E-06 | 21.45 |
| FL | Ptr-bHLH5_SNP 11 | TF gene | Promoter | 0.099 | 0.057 | 1.03E-05 | 20.94 |
| MAF | Ptr-AT-Hook2_SNP 3 | TF gene | Flanking | 1.431 | 5.591 | 1.07E-05 | 26.84 |
| HC | Ptr-TBP2_SNP 57 | TF gene | Intron |  | 13.073 | 1.14E-05 | 20.15 |
| MAF | Ptr-AT-Hook2_SNP 6 | TF gene | Flanking | 1.394 | 5.569 | 1.15E-05 | 26.64 |
| MAF | Ptr-AT-Hook2_SNP 4 | TF gene | Flanking | 1.394 | 5.569 | 1.15E-05 | 26.64 |
| MAF | Ptr-AT-Hook2_SNP 5 | TF gene | Flanking | 1.394 | 5.569 | 1.15E-05 | 26.64 |
| FW | Ptr-LAC27_SNP50 | Lignin biosynthesis gene | Promoter | 0.855 | 4.541 | 1.23E-05 | 25.48 |
| LC | Ptr-C4H3_SNP69 | Lignin biosynthesis gene | Promoter | 1.445 |  | 1.29E-05 | 18.13 |
| H | Ptr-HCT12_SNP59 | Lignin biosynthesis gene | CDS | 1.436 | 2.762 | 1.29E-05 | 22.41 |
| HC | Ptr-CCoAOMT3_SNP35 | Lignin biosynthesis gene | Promoter | 12.967 | 8.815 | 1.43E-05 | 22.71 |
| FW | Ptr-CAD18_SNP41 | Lignin biosynthesis gene | Flanking | 0.761 |  | 1.44E-05 | 20.43 |
| DBH | Ptr-PO23_SNP72 | Lignin biosynthesis gene | CDS | 7.913 | 2.615 | 1.60E-05 | 23.87 |
| FW | Ptr-LAC17_SNP42 | Lignin biosynthesis gene | Intron | 0.557 |  | 1.62E-05 | 20.29 |
| FW | Ptr-LAC17_SNP43 | Lignin biosynthesis gene | Intron | 0.389 |  | 1.62E-05 | 20.29 |
| DBH | L43_SNP 69 | LncRNA loci | LncRNA coding region | 5.210 | -0.222 | 1.63E-05 | 23.89 |
| FW | L17_SNP 66 | LncRNA loci | LncRNA coding region |  | 1.249 | 1.71E-05 | 20.13 |
| FW | Ptr-4CL9_SNP40 | Lignin biosynthesis gene | Intron |  | 1.249 | 1.71E-05 | 20.13 |
| V | Ptr-COMT9_SNP8 | Lignin biosynthesis gene | Flanking | 23.919 | 5.517 | 1.72E-05 | 23.57 |
| FW | L12_SNP 24 | LncRNA loci | LncRNA coding region | 1.497 |  | 1.80E-05 | 21.86 |
| FW | Ptr-PO16_SNP24 | Lignin biosynthesis gene | CDS | 1.497 |  | 1.80E-05 | 21.86 |
| HEC | Ptr-bHLH16_SNP 80 | TF gene | CDS | 13.981 | 11.088 | 1.83E-05 | 22.80 |
| DBH | Ptr-bHLH23_SNP 49 | TF gene | Intron | 0.111 | -4.222 | 1.87E-05 | 23.78 |
| HC | Ptr-C3H3_SNP7 | Lignin biosynthesis gene | Promoter | 0.514 | -20.164 | 1.89E-05 | 22.39 |
| DBH | Ptr-HD-ZIP51_SNP 113 | TF gene | Promoter | 2.816 | -1.268 | 1.94E-05 | 23.57 |
| V | L62_SNP 40 | LncRNA loci | Promoter | 30.608 | -31.147 | 2.00E-05 | 23.15 |
| DBH | Ptr-4CL3_SNP37 | Lignin biosynthesis gene | Intron | 2.584 | 8.645 | 2.10E-05 | 23.83 |
| DBH | L37_SNP 35 | LncRNA loci | LncRNA coding region | 2.164 | -2.845 | 2.14E-05 | 23.83 |
| DBH | Ptr-LAC22_SNP102 | Lignin biosynthesis gene | CDS | 2.013 | -4.094 | 2.15E-05 | 23.31 |
| FW | Ptr-LAC8_SNP27 | Lignin biosynthesis gene | CDS | 3.463 | -1.584 | 2.15E-05 | 23.69 |
| DBH | L19_SNP 8 | LncRNA loci | Promoter | 2.396 | 3.591 | 2.17E-05 | 23.36 |
| DBH | L27_SNP 69 | LncRNA loci | Promoter | 0.090 | 4.177 | 2.24E-05 | 23.94 |
| DBH | Ptr-PO65_SNP68 | Lignin biosynthesis gene | Intron | 4.837 | 0.353 | 2.27E-05 | 23.28 |
| LC | Ptr-LAC10_SNP112 | Lignin biosynthesis gene | Promoter | 2.101 | 0.173 | 2.36E-05 | 20.65 |
| FL | L49_SNP 100 | LncRNA loci | LncRNA coding region | 0.020 | -0.312 | 2.44E-05 | 22.44 |
| FL | L49_SNP 99 | LncRNA loci | LncRNA coding region | 0.020 | -0.312 | 2.44E-05 | 22.44 |
| V | Ptr-HD-ZIP43_SNP 83 | TF gene | Promoter | 22.706 | 33.557 | 2.44E-05 | 22.79 |
| DBH | L15_SNP 50 | LncRNA loci | Promoter | 2.701 | -0.928 | 2.55E-05 | 23.58 |
| V | Ptr-HD-ZIP13_SNP 102 | TF gene | 5' UTR | 1.497 | 0.870 | 2.69E-05 | 22.55 |
| FL | Ptr-F5H3_SNP29 | Lignin biosynthesis gene | Promoter | 0.086 |  | 2.70E-05 | 16.71 |
| HC | L70_SNP 30 | LncRNA loci | LncRNA coding region |  | 4.947 | 2.71E-05 | 18.45 |
| DBH | L19_SNP 63 | LncRNA loci | Promoter | 2.300 | 3.017 | 2.71E-05 | 22.94 |
| DBH | L44_SNP 65 | LncRNA loci | Flanking | 2.724 | 0.424 | 2.79E-05 | 23.25 |
| V | Ptr-4CL3_SNP4 | Lignin biosynthesis gene | Flanking | 25.558 | 32.811 | 2.84E-05 | 23.03 |
| DBH | Ptr-bHLH4_SNP 60 | TF gene | 3' UTR | 2.323 | 1.075 | 2.84E-05 | 23.74 |
| LC | L40_SNP 80 | LncRNA loci | LncRNA coding region | 0.296 | 1.768 | 2.89E-05 | 13.14 |
| LC | Ptr-HD-ZIP54_SNP 13 | TF gene | Intron |  | 1.985 | 2.91E-05 | 14.77 |
| MAF | Ptr-HD-ZIP56_SNP 148 | TF gene | CDS | 0.820 | -5.573 | 2.99E-05 | 22.86 |
| MAF | Ptr-HD-ZIP56_SNP 149 | TF gene | CDS | 0.820 | -5.573 | 2.99E-05 | 22.86 |
| DBH | Ptr-COMT1_SNP28 | Lignin biosynthesis gene | Promoter | 1.760 | 4.705 | 3.03E-05 | 23.43 |
| MAF | L40_SNP 245 | LncRNA loci | LncRNA coding region | 0.696 |  | 3.07E-05 | 19.34 |
| FL | L65_SNP 10 | LncRNA loci | Promoter | 0.092 |  | 3.09E-05 | 17.30 |
| DBH | L13_SNP 141 | LncRNA loci | LncRNA coding region | 3.269 | 1.817 | 3.09E-05 | 23.38 |
| FW | Ptr-PO30_SNP35 | Lignin biosynthesis gene | Promoter |  | 2.340 | 3.16E-05 | 19.07 |
| DBH | Ptr-PAL3_SNP110 | Lignin biosynthesis gene | Promoter | 2.688 | -1.079 | 3.16E-05 | 23.09 |
| DBH | Ptr-HD-ZIP28_SNP 65 | TF gene | Promoter | 2.156 | 4.806 | 3.19E-05 | 23.18 |
| DBH | Ptr-HD-ZIP13_SNP 6 | TF gene | Promoter | 1.497 | 0.870 | 3.25E-05 | 22.75 |
| DBH | Ptr-COMT25_SNP157 | Lignin biosynthesis gene | Promoter | 0.450 | -3.583 | 3.31E-05 | 22.69 |
| DBH | Ptr-COMT25_SNP158 | Lignin biosynthesis gene | Promoter | 0.450 | -3.583 | 3.31E-05 | 22.69 |
| HC | Ptr-PO82_SNP61 | Lignin biosynthesis gene | promoter |  | 3.064 | 3.36E-05 | 18.19 |
| HC | Ptr-CAD13_SNP44 | Lignin biosynthesis gene | Intron | 11.851 | 12.160 | 3.79E-05 | 20.16 |
| V | Ptr-CAD13_SNP44 | Lignin biosynthesis gene | Intron | 19.026 | -30.827 | 3.79E-05 | 21.98 |
| FW | Ptr-C3H3_SNP28 | Lignin biosynthesis gene | Promoter | 0.262 | 0.710 | 4.04E-05 | 22.02 |
| FW | L51_SNP 3 | LncRNA loci | Promoter |  | 1.490 | 4.05E-05 | 18.69 |
| V | Pto-MIR167d_SNP 27 | MiRNA gene | Flanking | 29.723 | 31.485 | 4.27E-05 | 21.14 |
| FW | Ptr-HD-ZIP40_SNP 14 | TF gene | Intron |  | 1.920 | 4.32E-05 | 18.86 |
| V | L19_SNP 36 | LncRNA loci | Promoter | 21.594 | -29.583 | 4.50E-05 | 22.56 |
| V | L70_SNP 38 | LncRNA loci | LncRNA coding region | 25.383 | 30.307 | 4.51E-05 | 23.05 |
| HC | Ptr-LAC26_SNP88 | Lignin biosynthesis gene | Promoter | 7.575 | 22.254 | 4.52E-05 | 20.58 |
| FW | Ptr-LAC17_SNP75 | Lignin biosynthesis gene | CDS |  | 1.594 | 4.66E-05 | 18.34 |
| FW | Ptr-TCR2_SNP 70 | TF gene | Intron | 3.490 | -1.703 | 4.68E-05 | 21.65 |
| FW | Ptr-CAD14_SNP1 | Lignin biosynthesis gene | Flanking |  | 1.435 | 4.70E-05 | 18.23 |
| FW | Ptr-PO91_SNP45 | Lignin biosynthesis gene | Promoter | 3.415 | -1.206 | 4.89E-05 | 22.61 |
| FL | Ptr-PO61_SNP43 | Lignin biosynthesis gene | Promoter | 0.014 | 0.032 | 4.89E-05 | 18.63 |
| H | Ptr-PAL4_SNP64 | Lignin biosynthesis gene | Flanking | 2.194 | -4.009 | 4.98E-05 | 21.02 |
| FW | Ptr-PO91_SNP46 | Lignin biosynthesis gene | Promoter | 3.413 | -1.206 | 5.02E-05 | 22.55 |
| MAF | L40_SNP 242 | LncRNA loci | LncRNA coding region | 1.366 |  | 5.21E-05 | 18.33 |
| MAF | L40_SNP 244 | LncRNA loci | LncRNA coding region | 1.546 |  | 5.21E-05 | 18.33 |
| FL | Ptr-PO46_SNP14 | Lignin biosynthesis gene | CDS | 0.017 |  | 5.25E-05 | 15.39 |
| MAF | Ptr-Myb/SANT1_SNP 17 | TF gene | Intron |  | -3.543 | 5.26E-05 | 18.32 |
| HC | Ptr-TBP2_SNP 56 | TF gene | Intron |  | 13.073 | 5.35E-05 | 20.65 |
| FW | Ptr-HD-ZIP53_SNP 101 | TF gene | Flanking |  | 1.265 | 5.66E-05 | 17.90 |
| FW | Ptr-HCT16_SNP80 | Lignin biosynthesis gene | Promoter | 0.342 | 1.895 | 5.72E-05 | 22.41 |
| FW | Ptr-HCT16_SNP81 | Lignin biosynthesis gene | Promoter | 0.342 | 1.895 | 5.72E-05 | 22.41 |
| HC | Ptr-bHLH11_SNP 45 | TF gene | Promoter | 12.318 | 11.383 | 5.79E-05 | 20.40 |
| V | L19_SNP 27 | LncRNA loci | Promoter | 28.887 | -18.392 | 5.83E-05 | 22.17 |
| HC | Ptr-bHLH23_SNP 98 | TF gene | Promoter | 8.911 | 3.037 | 5.83E-05 | 21.78 |
| LC | Ptr-bHLH23_SNP 98 | TF gene | Promoter | 1.705 | -0.167 | 5.83E-05 | 13.37 |
| H | Ptr-PAL4_SNP65 | Lignin biosynthesis gene | Flanking | 2.195 | -3.990 | 5.93E-05 | 20.17 |
| FW | Ptr-PO18_SNP95 | Lignin biosynthesis gene | Promoter |  | 1.065 | 5.96E-05 | 18.00 |
| FL | L08_SNP 44 | LncRNA loci | Promoter |  | -0.051 | 5.96E-05 | 15.76 |
| FL | L08_SNP 45 | LncRNA loci | Promoter |  | -0.051 | 5.96E-05 | 15.76 |
| FL | Ptr-COMT25_SNP19 | Lignin biosynthesis gene | 3' UTR |  | -0.051 | 5.96E-05 | 15.76 |
| FL | Ptr-COMT25_SNP20 | Lignin biosynthesis gene | 3' UTR |  | -0.051 | 5.96E-05 | 15.76 |
| FW | Ptr-CAD8_SNP3 | Lignin biosynthesis gene | Intron |  | 4.022 | 6.08E-05 | 17.98 |
| FW | Ptr-PO37_SNP47 | Lignin biosynthesis gene | Promoter |  | 2.090 | 6.20E-05 | 18.99 |
| LC | Ptr-4CL20X_SNP116 | Lignin biosynthesis gene | Intron | 1.326 | 2.152 | 6.28E-05 | 19.07 |
| FL | Ptr-PO67_SNP40 | Lignin biosynthesis gene | Promoter | 0.001 | -0.328 | 6.33E-05 | 20.68 |
| LC | Ptr-CCoAOMT4_SNP21 | Lignin biosynthesis gene | Promoter | 1.322 |  | 6.57E-05 | 18.78 |
| CC | Ptr-bHLH1_SNP 11 | TF gene | Intron | 2.822 |  | 6.63E-05 | 17.45 |
| FW | Ptr-LAC13_SNP12 | Lignin biosynthesis gene | Intron |  | 1.327 | 6.69E-05 | 17.62 |
| LC | L57_SNP 8 | LncRNA loci | Promoter | 1.860 | 3.564 | 6.78E-05 | 17.58 |
| LC | L57_SNP 9 | LncRNA loci | Promoter | 1.860 | 3.564 | 6.78E-05 | 17.58 |
| FW | L60_SNP 160 | LncRNA loci | LncRNA coding region | 0.976 |  | 6.83E-05 | 17.72 |
| FW | Ptr-4CL17_SNP119 | Lignin biosynthesis gene | Intron | 0.976 |  | 6.83E-05 | 17.72 |

**Table S6.** Detailed information of significant epistatic SNP-SNP pairs for each trait in the association population of *P. tomentosa*.

| **SNP 1** | **Annotation** | **SNP 2** | **Annotation** | **Associated traits** | **Test** | **Effect** | ***P-*value** |
| --- | --- | --- | --- | --- | --- | --- | --- |
| L40_SNP30 | *LncRNA locus 40* | Ptr-HD-ZIP28_SNP58 | *Ptr-HD-ZIP28* | HC | DA | -39.30 | 2.24E-08 |
| L40_SNP30 | *LncRNA locus 40* | Ptr-HD-ZIP28_SNP59 | *Ptr-HD-ZIP28* | HC | DA | -39.30 | 2.24E-08 |
| L13_SNP23 | *LncRNA locus 13* | Ptr-C2H2-72_SNP33 | *Ptr-C2H2-72* | V | AA | 0.85 | 3.91E-08 |
| Ptr-CCR13_SNP7 | *Ptr-CCR13* | L19_SNP32 | *LncRNA locus 19* | V | DA | -0.71 | 4.52E-08 |
| L40_SNP30 | *LncRNA locus 40* | Ptr-HD-ZIP28_SNP60 | *Ptr-HD-ZIP28* | HC | DA | -38.00 | 8.12E-08 |
| L40_SNP30 | *LncRNA locus 40* | Ptr-HD-ZIP28_SNP61 | *Ptr-HD-ZIP28* | HC | DA | 38.00 | 8.12E-08 |
| Ptr-CCR13_SNP7 | *Ptr-CCR13* | L19_SNP33 | *LncRNA locus 19* | V | DA | 0.69 | 1.15E-07 |
| Ptr-PO70_SNP63 | *Ptr-PO70* | Ptr-PO85_SNP15 | *Ptr-PO85* | V | DA | -1.63 | 1.65E-07 |
| Ptr-PO5_SNP65 | *Ptr-PO5* | Ptr-PO70_SNP63 | *Ptr-PO70* | V | DD | 2.28 | 1.89E-07 |
| Ptr-CAD13_SNP44 | *Ptr-CAD13* | Ptr-PO85_SNP1 | *Ptr-PO85* | HC | AA | 15.00 | 3.16E-07 |
| Ptr-COMT25_SNP110 | *Ptr-COMT25* | Ptr-Myb/SANT2_SNP21 | *Ptr-Myb/SANT2* | HEC | AD | -23.90 | 3.57E-07 |
| Ptr-HD-ZIP13_SNP2 | *Ptr-HD-ZIP13* | Ptr-HCT8_SNP58 | *Ptr-HCT8* | HC | AD | 23.10 | 3.66E-07 |
| Ptr-LAC4_SNP49 | *Ptr-LAC4* | Ptr-HD-ZIP24_SNP81 | *Ptr-HD-ZIP24* | HC | DD | 38.70 | 3.94E-07 |
| Ptr-LAC4_SNP50 | *Ptr-LAC4* | Ptr-HD-ZIP24_SNP81 | *Ptr-HD-ZIP24* | HC | DD | 38.70 | 3.94E-07 |
| L40_SNP30 | *LncRNA locus 40* | Ptr-4CL4_SNP76 | *Ptr-4CL4* | HC | DA | 28.10 | 4.29E-07 |
| Ptr-COMT25_SNP109 | *Ptr-COMT25* | Ptr-Myb/SANT2_SNP21 | *Ptr-Myb/SANT2* | HEC | AD | -23.90 | 4.30E-07 |
| Ptr-COMT25_SNP109 | *Ptr-COMT25* | Ptr-HD-ZIP24_SNP81 | *Ptr-HD-ZIP24* | V | AA | 0.42 | 4.50E-07 |
| Ptr-COMT25_SNP110 | *Ptr-COMT25* | Ptr-HD-ZIP24_SNP81 | *Ptr-HD-ZIP24* | V | AA | 0.42 | 4.50E-07 |
| Ptr-CCR13_SNP7 | *Ptr-CCR13* | L35_SNP13 | *LncRNA locus 35* | V | DA | -0.62 | 4.54E-07 |
| Ptr-COMT25_SNP107 | *Ptr-COMT25* | Pto-MiR167d_SNP42 | *Pto-MIR167d* | V | DD | 2.05 | 4.88E-07 |
| L19_SNP31 | *LncRNA locus 19* | Ptr-CCR9_SNP20 | *Ptr-CCR9* | V | AD | -0.97 | 4.94E-07 |
| Ptr-bHLH17_SNP5 | *Ptr-bHLH17* | Ptr-PO70_SNP64 | *Ptr-PO70* | V | AD | -1.52 | 5.59E-07 |
| Ptr-bHLH11_SNP45 | *Ptr-bHLH11* | Ptr-PO85_SNP1 | *Ptr-PO85* | HC | AA | 15.90 | 5.68E-07 |
| Ptr-CCR13_SNP7 | *Ptr-CCR13* | Ptr-PO40_SNP142 | *Ptr-PO40* | V | AD | 0.57 | 5.84E-07 |
| Ptr-HCT1_SNP28 | *Ptr-HCT1* | Ptr-CAD16_SNP98 | *Ptr-CAD16* | V | AA | 0.85 | 6.11E-07 |
| L19_SNP31 | *LncRNA locus 19* | L37_SNP18 | *LncRNA locus 37* | HC | DD | 33.90 | 6.98E-07 |
| Ptr-COMT25_SNP110 | *Ptr-COMT25* | Ptr-HCT13_SNP37 | *Ptr-HCT13* | V | AA | -0.40 | 7.43E-07 |
| Ptr-HCT12_SNP87 | *Ptr-HCT12* | Ptr-LAC43_SNP28 | *Ptr-LAC43* | HC | AA | 15.50 | 7.44E-07 |
| Ptr-COMT25_SNP110 | *Ptr-COMT25* | Pto-MiR167d_SNP42 | *Pto-MIR167d* | V | DD | 1.99 | 7.49E-07 |
| Ptr-COMT25_SNP109 | *Ptr-COMT25* | Ptr-HCT13_SNP37 | *Ptr-HCT13* | V | AA | -0.41 | 7.85E-07 |
| Ptr-PO23_SNP4 | *Ptr-PO23* | Ptr-Myb/SANT2_SNP21 | *Ptr-Myb/SANT2* | HEC | AD | 24.60 | 7.89E-07 |
| Ptr-COMT25_SNP95 | *Ptr-COMT25* | Ptr-HD-ZIP24_SNP81 | *Ptr-HD-ZIP24* | V | AA | 0.66 | 8.37E-07 |
| Ptr-COMT25_SNP109 | *Ptr-COMT25* | Pto-MiR167d_SNP42 | *Pto-MIR167d* | V | DD | 1.99 | 8.56E-07 |
| Ptr-HD-ZIP49_SNP84 | *Ptr-HD-ZIP49* | Ptr-HD-ZIP28_SNP21 | *Ptr-HD-ZIP28* | V | AA | -0.51 | 9.00E-07 |
| L09_SNP32 | *LncRNA locus 9* | Ptr-C4H3_SNP108 | *Ptr-C4H3* | HC | AA | 11.30 | 9.31E-07 |
| Ptr-PO70_SNP75 | *Ptr-PO70* | Ptr-C2H2-77_SNP70 | *Ptr-C2H2-77* | V | DA | 0.42 | 9.51E-07 |
| L39_SNP52 | *LncRNA locus 39* | Ptr-C2H2-77_SNP57 | *Ptr-C2H2-77* | HC | AA | 12.10 | 1.08E-06 |
| L39_SNP53 | *LncRNA locus 39* | Ptr-C2H2-77_SNP57 | *Ptr-C2H2-77* | HC | AA | -12.10 | 1.08E-06 |
| Ptr-PO23_SNP4 | *Ptr-PO23* | L62_SNP113 | *LncRNA locus 62* | CC | AA | -9.57 | 1.16E-06 |
| Ptr-CCR13_SNP7 | *Ptr-CCR13* | Ptr-HD-ZIP13_SNP64 | *Ptr-HD-ZIP13* | V | DA | 0.63 | 1.16E-06 |
| Ptr-COMT25_SNP107 | *Ptr-COMT25* | Ptr-HD-ZIP24_SNP81 | *Ptr-HD-ZIP24* | V | AA | 0.41 | 1.17E-06 |
| Ptr-bHLH10_SNP39 | *Ptr-bHLH10* | Ptr-HCT12_SNP9 | *Ptr-HCT12* | V | AD | 0.53 | 1.26E-06 |
| Ptr-PO70_SNP19 | *Ptr-PO70* | Ptr-4CL2_SNP29 | *Ptr-4CL2* | HC | DD | -41.90 | 1.32E-06 |
| Ptr-HD-ZIP4_SNP11 | *Ptr-HD-ZIP4* | Ptr-CAD10_SNP77 | *Ptr-CAD10* | V | AA | 0.54 | 1.40E-06 |
| Ptr-4CL3_SNP44 | *Ptr-4CL3* | L49_SNP33 | *LncRNA locus 49* | V | AD | 1.65 | 1.50E-06 |
| Ptr-4CL3_SNP44 | *Ptr-4CL3* | L49_SNP34 | *LncRNA locus 49* | V | AD | 1.65 | 1.50E-06 |
| L10_SNP110 | *LncRNA locus 10* | L40_SNP30 | *LncRNA locus 40* | HC | AD | 26.30 | 1.53E-06 |
| Ptr-COMT25_SNP119 | *Ptr-COMT25* | Ptr-COMT22_SNP58 | *Ptr-COMT22* | HC | AA | -26.20 | 1.61E-06 |
| Ptr-bHLH2_SNP16 | *Ptr-bHLH2* | L49_SNP33 | *LncRNA locus 49* | V | AD | 1.40 | 1.65E-06 |
| Ptr-bHLH2_SNP16 | *Ptr-bHLH2* | L49_SNP34 | *LncRNA locus 49* | V | AD | 1.40 | 1.65E-06 |
| Ptr-COMT25_SNP111 | *Ptr-COMT25* | Ptr-HCT13_SNP37 | *Ptr-HCT13* | V | AA | 0.38 | 1.82E-06 |
| L49_SNP15 | *LncRNA locus 49* | Ptr-PO70_SNP75 | *Ptr-PO70* | V | AA | 0.46 | 1.91E-06 |
| Ptr-COMT25_SNP109 | *Ptr-COMT25* | Ptr-HD-ZIP24_SNP81 | *Ptr-HD-ZIP24* | DBH | AA | 5.79 | 1.98E-06 |
| Ptr-COMT25_SNP110 | *Ptr-COMT25* | Ptr-HD-ZIP24_SNP81 | *Ptr-HD-ZIP24* | DBH | AA | 5.79 | 1.98E-06 |
| Ptr-PO23_SNP8 | *Ptr-PO23* | L33_SNP12 | *LncRNA locus 33* | HC | AD | 12.50 | 2.03E-06 |
| Ptr-AT-Hook2_SNP90 | *Ptr-AT-Hook2* | Ptr-4CL4_SNP69 | *Ptr-4CL4* | V | DD | -1.68 | 2.09E-06 |
| Ptr-COMT25_SNP111 | *Ptr-COMT25* | Pto-MiR167d_SNP42 | *Pto-MIR167d* | V | DD | 1.94 | 2.12E-06 |
| Ptr-COMT25_SNP107 | *Ptr-COMT25* | Ptr-COMT22_SNP58 | *Ptr-COMT22* | HC | DA | 17.90 | 2.26E-06 |
| L26_SNP42 | *LncRNA locus 26* | Ptr-C4H3_SNP100 | *Ptr-C4H3* | V | AA | 0.30 | 2.30E-06 |
| Ptr-CAD10_SNP184 | *Ptr-CAD10* | Ptr-bHLH15_SNP56 | *Ptr-bHLH15* | V | DA | -0.39 | 2.32E-06 |
| Ptr-COMT25_SNP109 | *Ptr-COMT25* | L68_SNP1 | *LncRNA locus 68* | HC | DA | 22.50 | 2.33E-06 |
| Ptr-PO19_SNP86 | *Ptr-PO19* | L49_SNP33 | *LncRNA locus 49* | V | AD | 1.45 | 2.38E-06 |
| Ptr-PO19_SNP86 | *Ptr-PO19* | L49_SNP34 | *LncRNA locus 49* | V | AD | 1.45 | 2.38E-06 |
| L49_SNP33 | *LncRNA locus 49* | Ptr-HD-ZIP28_SNP58 | *Ptr-HD-ZIP28* | V | DA | 1.62 | 2.41E-06 |
| L49_SNP34 | *LncRNA locus 49* | Ptr-HD-ZIP28_SNP58 | *Ptr-HD-ZIP28* | V | DA | 1.62 | 2.41E-06 |
| L49_SNP33 | *LncRNA locus 49* | Ptr-HD-ZIP28_SNP59 | *Ptr-HD-ZIP28* | V | DA | 1.62 | 2.41E-06 |
| L49_SNP34 | *LncRNA locus 49* | Ptr-HD-ZIP28_SNP59 | *Ptr-HD-ZIP28* | V | DA | 1.62 | 2.41E-06 |
| L19_SNP31 | *LncRNA locus 19* | Ptr-CCR9_SNP19 | *Ptr-CCR9* | V | AD | -0.91 | 2.60E-06 |
| Ptr-CAD16_SNP98 | *Ptr-CAD16* | Ptr-4CL20_SNP93 | *Ptr-4CL20* | HC | AD | 35.20 | 2.62E-06 |
| Ptr-COMT25_SNP107 | *Ptr-COMT25* | Ptr-HCT13_SNP37 | *Ptr-HCT13* | V | AA | -0.38 | 2.63E-06 |
| Ptr-PAL3_SNP83 | *Ptr-PAL3* | Ptr-C2H2-77_SNP57 | *Ptr-C2H2-77* | HC | AA | 16.60 | 2.72E-06 |
| Ptr-PO31_SNP47 | *Ptr-PO31* | L49_SNP33 | *LncRNA locus 49* | V | AD | 1.63 | 2.73E-06 |
| Ptr-PO31_SNP47 | *Ptr-PO31* | L49_SNP34 | *LncRNA locus 49* | V | AD | 1.63 | 2.73E-06 |
| L09_SNP30 | *LncRNA locus 9* | L19_SNP19 | *LncRNA locus 19* | HC | DA | 20.10 | 2.77E-06 |
| Ptr-HD-ZIP24_SNP81 | *Ptr-HD-ZIP24* | Ptr-HCT2_SNP48 | *Ptr-HCT2* | V | AA | 0.74 | 2.78E-06 |
| Ptr-COMT25_SNP110 | *Ptr-COMT25* | L68_SNP1 | *LncRNA locus 68* | HC | DA | 22.30 | 2.79E-06 |
| Ptr-Myb/SANT2_SNP21 | *Ptr-Myb/SANT2* | Ptr-bHLH18_SNP21 | *Ptr-bHLH18* | HEC | DA | -22.30 | 2.83E-06 |
| Ptr-PO41_SNP7 | *Ptr-PO41* | Ptr-LAC31_SNP6 | *Ptr-LAC31* | HC | AA | 22.10 | 2.88E-06 |
| Ptr-bHLH10_SNP39 | *Ptr-bHLH10* | Ptr-COMT22_SNP58 | *Ptr-COMT22* | DBH | AA | 5.02 | 2.92E-06 |
| L19_SNP138 | *LncRNA locus 19* | Ptr-4CL4_SNP70 | *Ptr-4CL4* | V | AA | 0.67 | 2.95E-06 |
| L19_SNP138 | *LncRNA locus 19* | Ptr-4CL4_SNP71 | *Ptr-4CL4* | V | AA | 0.67 | 2.95E-06 |
| Ptr-CCR13_SNP7 | *Ptr-CCR13* | L19_SNP39 | *LncRNA locus 19* | V | DA | 0.59 | 3.00E-06 |
| Ptr-COMT25_SNP110 | *Ptr-COMT25* | Ptr-COMT22_SNP58 | *Ptr-COMT22* | HC | DA | 16.90 | 3.00E-06 |
| Ptr-C3H3_SNP7 | *Ptr-C3H3* | Ptr-bZIP5_SNP85 | *Ptr-bZIP5* | V | AD | -1.19 | 3.04E-06 |
| Ptr-C2H2-77_SNP70 | *Ptr-C2H2-77* | Ptr-HD-ZIP28_SNP21 | *Ptr-HD-ZIP28* | V | AA | 0.42 | 3.11E-06 |
| L10_SNP110 | *LncRNA locus 10* | Ptr-COMT22_SNP58 | *Ptr-COMT22* | HC | AA | -22.80 | 3.19E-06 |
| Ptr-4CL4_SNP70 | *Ptr-4CL4* | Ptr-COMT12_SNP122 | *Ptr-COMT12* | V | DD | -1.16 | 3.33E-06 |
| Ptr-4CL4_SNP71 | *Ptr-4CL4* | Ptr-COMT12_SNP122 | *Ptr-COMT12* | V | DD | -1.16 | 3.33E-06 |
| L33_SNP50 | *LncRNA locus 33* | Ptr-Myb/SANT2_SNP21 | *Ptr-Myb/SANT2* | HEC | AD | -21.20 | 3.35E-06 |
| L19_SNP138 | *LncRNA locus 19* | Ptr-4CL4_SNP72 | *Ptr-4CL4* | V | AA | -0.67 | 3.41E-06 |
| Ptr-COMT25_SNP95 | *Ptr-COMT25* | Ptr-HCT13_SNP37 | *Ptr-HCT13* | V | AA | -0.52 | 3.41E-06 |
| Ptr-COMT25_SNP126 | *Ptr-COMT25* | Ptr-PO70_SNP63 | *Ptr-PO70* | V | AD | -1.27 | 3.41E-06 |
| Ptr-CCR13_SNP7 | *Ptr-CCR13* | L19_SNP36 | *LncRNA locus 19* | V | DA | 0.66 | 3.44E-06 |
| Ptr-COMT25_SNP104 | *Ptr-COMT25* | Ptr-COMT22_SNP58 | *Ptr-COMT22* | HC | DA | 17.30 | 3.44E-06 |
| Ptr-CAD13_SNP44 | *Ptr-CAD13* | L33_SNP4 | *LncRNA locus 33* | HC | AA | -20.00 | 3.48E-06 |
| Ptr-C3H2_SNP39 | *Ptr-C3H2* | Ptr-LAC43_SNP28 | *Ptr-LAC43* | HC | AA | -16.00 | 3.48E-06 |
| Ptr-PO5_SNP62 | *Ptr-PO5* | Ptr-LAC4_SNP49 | *Ptr-LAC4* | HC | DD | 33.60 | 3.74E-06 |
| Ptr-PO5_SNP62 | *Ptr-PO5* | Ptr-LAC4_SNP50 | *Ptr-LAC4* | HC | DD | 33.60 | 3.74E-06 |
| Ptr-COMT25_SNP111 | *Ptr-COMT25* | Ptr-HD-ZIP24_SNP81 | *Ptr-HD-ZIP24* | V | AA | -0.38 | 3.76E-06 |
| Ptr-COMT25_SNP109 | *Ptr-COMT25* | Ptr-COMT22_SNP58 | *Ptr-COMT22* | HC | DA | 16.90 | 3.83E-06 |
| Ptr-bHLH4_SNP42 | *Ptr-bHLH4* | Ptr-HCT12_SNP88 | *Ptr-HCT12* | HC | DA | -9.26 | 3.85E-06 |
| L19_SNP138 | *LncRNA locus 19* | Ptr-HD-ZIP21_SNP40 | *Ptr-HD-ZIP21* | V | DA | -0.34 | 3.99E-06 |
| Ptr-PO70_SNP63 | *Ptr-PO70* | Ptr-bHLH23_SNP29 | *Ptr-bHLH23* | V | DA | 1.24 | 4.01E-06 |
| Ptr-COMT25_SNP95 | *Ptr-COMT25* | Ptr-HD-ZIP24_SNP81 | *Ptr-HD-ZIP24* | DBH | AA | 8.97 | 4.07E-06 |
| Ptr-HCT12_SNP9 | *Ptr-HCT12* | L40_SNP2 | *LncRNA locus 40* | V | AD | -1.19 | 4.10E-06 |
| Ptr-bHLH13_SNP39 | *Ptr-bHLH13* | L49_SNP33 | *LncRNA locus 49* | V | DD | -2.03 | 4.14E-06 |
| Ptr-bHLH13_SNP39 | *Ptr-bHLH13* | L49_SNP34 | *LncRNA locus 49* | V | DD | -2.03 | 4.14E-06 |
| Ptr-HCT1_SNP22 | *Ptr-HCT1* | Ptr-CAD16_SNP98 | *Ptr-CAD16* | V | AA | -0.88 | 4.26E-06 |
| Ptr-HD-ZIP55_SNP30 | *Ptr-HD-ZIP55* | Ptr-PO82_SNP87 | *Ptr-PO82* | V | AD | -1.19 | 4.29E-06 |
| L49_SNP33 | *LncRNA locus 49* | L58_SNP20 | *LncRNA locus 58* | V | DA | -1.34 | 4.33E-06 |
| L49_SNP34 | *LncRNA locus 49* | L58_SNP20 | *LncRNA locus 58* | V | DA | -1.34 | 4.33E-06 |
| Ptr-COMT25_SNP107 | *Ptr-COMT25* | Ptr-Myb/SANT2_SNP21 | *Ptr-Myb/SANT2* | HEC | AD | -20.10 | 4.41E-06 |
| Ptr-PO54_SNP54 | *Ptr-PO54* | Ptr-CCoAOMT5_SNP20 | *Ptr-CCoAOMT5* | LC | DD | -7.50 | 4.46E-06 |
| Ptr-CAD10_SNP198 | *Ptr-CAD10* | Ptr-HD-ZIP49_SNP64 | *Ptr-HD-ZIP49* | HC | DA | 17.70 | 4.56E-06 |
| Ptr-CCR26_SNP4 | *Ptr-CCR26* | Ptr-Myb/SANT2_SNP21 | *Ptr-Myb/SANT2* | HEC | AD | -19.40 | 4.64E-06 |
| L06_SNP61 | *LncRNA locus 6* | Ptr-F5H1_SNP48 | *Ptr-F5H1* | V | DA | -0.75 | 4.67E-06 |
| L02_SNP23 | *LncRNA locus 2* | Ptr-COMT22_SNP58 | *Ptr-COMT22* | HC | AA | -21.30 | 4.76E-06 |
| Ptr-bHLH4_SNP44 | *Ptr-bHLH4* | Ptr-HCT12_SNP88 | *Ptr-HCT12* | HC | DA | -9.01 | 4.77E-06 |
| Ptr-bHLH4_SNP45 | *Ptr-bHLH4* | Ptr-HCT12_SNP88 | *Ptr-HCT12* | HC | DA | -9.01 | 4.77E-06 |
| Ptr-4CL4_SNP72 | *Ptr-4CL4* | Ptr-COMT12_SNP122 | *Ptr-COMT12* | V | DD | -1.14 | 4.81E-06 |
| Ptr-CAD10_SNP214 | *Ptr-CAD10* | L49_SNP33 | *LncRNA locus 49* | V | AD | -1.27 | 4.86E-06 |
| Ptr-CAD10_SNP214 | *Ptr-CAD10* | L49_SNP34 | *LncRNA locus 49* | V | AD | -1.27 | 4.86E-06 |
| Ptr-4CL12_SNP28 | *Ptr-4CL12* | Ptr-CCoAOMT5_SNP20 | *Ptr-CCoAOMT5* | HC | AA | -10.80 | 4.94E-06 |
| L50_SNP110 | *LncRNA locus 50* | Ptr-PO70_SNP63 | *Ptr-PO70* | V | AD | 1.14 | 5.00E-06 |
| Ptr-PO23_SNP8 | *Ptr-PO23* | L40_SNP30 | *LncRNA locus 40* | HC | AD | 20.10 | 5.03E-06 |
| Ptr-CAD10_SNP215 | *Ptr-CAD10* | L49_SNP33 | *LncRNA locus 49* | V | AD | -1.52 | 5.03E-06 |
| Ptr-CAD10_SNP215 | *Ptr-CAD10* | L49_SNP34 | *LncRNA locus 49* | V | AD | -1.52 | 5.03E-06 |
| Ptr-COMT25_SNP112 | *Ptr-COMT25* | L49_SNP33 | *LncRNA locus 49* | V | AD | 1.31 | 5.30E-06 |
| Ptr-COMT25_SNP112 | *Ptr-COMT25* | L49_SNP34 | *LncRNA locus 49* | V | AD | 1.31 | 5.30E-06 |
| Pto-MiR475d_SNP7 | *Pto-MIR475d* | Ptr-PO82_SNP87 | *Ptr-PO82* | V | AD | -0.90 | 5.30E-06 |
| Ptr-COMT23_SNP5 | *Ptr-COMT23* | Ptr-C4H3_SNP108 | *Ptr-C4H3* | HC | AA | 15.80 | 5.47E-06 |
| Pto-MiR167a_SNP11 | *Pto-MIR167a* | Ptr-COMT22_SNP58 | *Ptr-COMT22* | HC | AA | 22.40 | 5.60E-06 |
| Ptr-C2H2-72_SNP61 | *Ptr-C2H2-72* | Ptr-HCT2_SNP9 | *Ptr-HCT2* | V | DD | -1.27 | 5.74E-06 |
| Ptr-bZIP5_SNP69 | *Ptr-bZIP5* | L58_SNP8 | *LncRNA locus 58* | V | DA | -1.31 | 5.79E-06 |
| Ptr-4CL3_SNP44 | *Ptr-4CL3* | Ptr-COMT22_SNP58 | *Ptr-COMT22* | HC | AA | 23.50 | 5.84E-06 |
| Ptr-HD-ZIP24_SNP81 | *Ptr-HD-ZIP24* | L42_SNP3 | *LncRNA locus 42* | HC | AA | 22.60 | 6.02E-06 |
| L49_SNP33 | *LncRNA locus 49* | Ptr-HD-ZIP28_SNP60 | *Ptr-HD-ZIP28* | V | DA | 1.59 | 6.07E-06 |
| L49_SNP34 | *LncRNA locus 49* | Ptr-HD-ZIP28_SNP60 | *Ptr-HD-ZIP28* | V | DA | 1.59 | 6.07E-06 |
| L49_SNP33 | *LncRNA locus 49* | Ptr-HD-ZIP28_SNP61 | *Ptr-HD-ZIP28* | V | DA | -1.59 | 6.07E-06 |
| L49_SNP34 | *LncRNA locus 49* | Ptr-HD-ZIP28_SNP61 | *Ptr-HD-ZIP28* | V | DA | -1.59 | 6.07E-06 |
| L25_SNP2 | *LncRNA locus 25* | Ptr-AT-Hook4_SNP58 | *Ptr-AT-Hook4* | HC | DD | -32.10 | 6.09E-06 |
| Ptr-PO5_SNP22 | *Ptr-PO5* | Ptr-LAC31_SNP6 | *Ptr-LAC31* | V | AA | 0.29 | 6.31E-06 |
| Ptr-CCR9_SNP137 | *Ptr-CCR9* | Ptr-COMT22_SNP58 | *Ptr-COMT22* | HC | AA | 21.60 | 6.38E-06 |
| Ptr-CCR9_SNP139 | *Ptr-CCR9* | Ptr-COMT22_SNP58 | *Ptr-COMT22* | HC | AA | -21.60 | 6.38E-06 |
| Ptr-PO34_SNP14 | *Ptr-PO34* | L40_SNP2 | *LncRNA locus 40* | V | AD | 1.22 | 6.40E-06 |
| Ptr-CAD10_SNP184 | *Ptr-CAD10* | Ptr-CAD14_SNP81 | *Ptr-CAD14* | V | DD | 2.00 | 6.41E-06 |
| L17_SNP4 | *LncRNA locus 17* | L19_SNP138 | *LncRNA locus 19* | HC | AA | -8.36 | 6.50E-06 |
| L26_SNP42 | *LncRNA locus 26* | Ptr-HD-ZIP28_SNP21 | *Ptr-HD-ZIP28* | V | AA | -0.21 | 6.50E-06 |
| Ptr-HCT1_SNP20 | *Ptr-HCT1* | Ptr-COMT22_SNP58 | *Ptr-COMT22* | HC | AA | 17.20 | 6.59E-06 |
| Ptr-COMT25_SNP107 | *Ptr-COMT25* | Ptr-HD-ZIP24_SNP81 | *Ptr-HD-ZIP24* | DBH | AA | 5.29 | 6.76E-06 |
| Ptr-4CL5_SNP149 | *Ptr-4CL5* | L49_SNP33 | *LncRNA locus 49* | V | AD | 1.31 | 6.77E-06 |
| Ptr-4CL5_SNP149 | *Ptr-4CL5* | L49_SNP34 | *LncRNA locus 49* | V | AD | 1.31 | 6.77E-06 |
| Ptr-HCT12_SNP9 | *Ptr-HCT12* | Ptr-C2H2-77_SNP70 | *Ptr-C2H2-77* | V | DA | 0.44 | 6.79E-06 |
| Ptr-CCR26_SNP4 | *Ptr-CCR26* | Ptr-PO82_SNP87 | *Ptr-PO82* | V | AD | 0.89 | 6.97E-06 |
| Ptr-PO17_SNP20 | *Ptr-PO17* | Ptr-PO70_SNP64 | *Ptr-PO70* | HC | AA | 20.90 | 7.01E-06 |
| L40_SNP30 | *LncRNA locus 40* | Ptr-4CL4_SNP72 | *Ptr-4CL4* | HC | DA | -28.40 | 7.13E-06 |
| Ptr-HD-ZIP10_SNP76 | *Ptr-HD-ZIP10* | Ptr-Myb/SANT2_SNP21 | *Ptr-Myb/SANT2* | HEC | AD | -20.90 | 7.21E-06 |
| Ptr-AT-Hook2_SNP85 | *Ptr-AT-Hook2* | Ptr-PO70_SNP64 | *Ptr-PO70* | V | AD | 1.39 | 7.21E-06 |
| Ptr-COMT25_SNP109 | *Ptr-COMT25* | Ptr-PO72_SNP18 | *Ptr-PO72* | HC | DA | 13.50 | 7.31E-06 |
| L13_SNP23 | *LncRNA locus 13* | Ptr-C2H2-72_SNP33 | *Ptr-C2H2-72* | DBH | AA | 10.40 | 7.35E-06 |
| Ptr-COMT22_SNP58 | *Ptr-COMT22* | Ptr-4CL12_SNP8 | *Ptr-4CL12* | HC | AA | 18.40 | 7.41E-06 |
| Ptr-COMT25_SNP110 | *Ptr-COMT25* | Ptr-HCT13_SNP37 | *Ptr-HCT13* | DBH | AA | -5.41 | 7.55E-06 |
| L40_SNP2 | *LncRNA locus 40* | Ptr-HCT13_SNP37 | *Ptr-HCT13* | V | DA | 1.16 | 7.58E-06 |
| Ptr-COMT25_SNP107 | *Ptr-COMT25* | Ptr-PO72_SNP18 | *Ptr-PO72* | HC | DA | 13.90 | 7.66E-06 |
| L33_SNP4 | *LncRNA locus 33* | Ptr-HD-ZIP55_SNP2 | *Ptr-HD-ZIP55* | HC | AA | 17.30 | 7.78E-06 |
| L40_SNP30 | *LncRNA locus 40* | Ptr-4CL4_SNP70 | *Ptr-4CL4* | HC | DA | 28.30 | 7.85E-06 |
| L40_SNP30 | *LncRNA locus 40* | Ptr-4CL4_SNP71 | *Ptr-4CL4* | HC | DA | 28.30 | 7.85E-06 |
| Ptr-LAC2_SNP20 | *Ptr-LAC2* | Ptr-PO70_SNP63 | *Ptr-PO70* | V | AD | 1.52 | 7.86E-06 |
| Ptr-HD-ZIP4_SNP11 | *Ptr-HD-ZIP4* | Ptr-HCT13_SNP37 | *Ptr-HCT13* | V | AD | -0.60 | 8.07E-06 |
| Ptr-C3H3_SNP15 | *Ptr-C3H3* | Ptr-C2H2-72_SNP33 | *Ptr-C2H2-72* | V | AA | 0.70 | 8.54E-06 |
| Ptr-PO40_SNP145 | *Ptr-PO40* | Ptr-bHLH18_SNP47 | *Ptr-bHLH18* | V | DD | -1.26 | 8.55E-06 |
| Ptr-COMT25_SNP109 | *Ptr-COMT25* | Ptr-HCT13_SNP37 | *Ptr-HCT13* | DBH | AA | -5.54 | 8.57E-06 |
| Ptr-bHLH10_SNP39 | *Ptr-bHLH10* | Ptr-COMT22_SNP58 | *Ptr-COMT22* | V | AA | 0.31 | 8.60E-06 |
| L13_SNP145 | *LncRNA locus 13* | Ptr-PO70_SNP63 | *Ptr-PO70* | V | AD | -1.46 | 8.67E-06 |
| Ptr-HD-ZIP24_SNP81 | *Ptr-HD-ZIP24* | L42_SNP4 | *LncRNA locus 42* | HC | AA | -23.10 | 8.78E-06 |
| L13_SNP120 | *LncRNA locus 13* | Ptr-HD-ZIP49_SNP64 | *Ptr-HD-ZIP49* | HC | DA | -12.40 | 8.80E-06 |
| Ptr-HD-ZIP27_SNP62 | *Ptr-HD-ZIP27* | Ptr-C2H2-72_SNP33 | *Ptr-C2H2-72* | V | AA | 0.69 | 8.98E-06 |
| Ptr-HD-ZIP27_SNP63 | *Ptr-HD-ZIP27* | Ptr-C2H2-72_SNP33 | *Ptr-C2H2-72* | V | AA | 0.69 | 8.98E-06 |
| Ptr-HCT1_SNP8 | *Ptr-HCT1* | Ptr-CAD16_SNP98 | *Ptr-CAD16* | V | AA | 0.84 | 8.98E-06 |
| Ptr-PAL3_SNP83 | *Ptr-PAL3* | Ptr-CCR26_SNP165 | *Ptr-CCR26* | HC | AA | 8.80 | 9.00E-06 |
| Ptr-PAL3_SNP102 | *Ptr-PAL3* | Ptr-HD-ZIP21_SNP40 | *Ptr-HD-ZIP21* | V | AA | 0.37 | 9.05E-06 |
| Ptr-bZIP5_SNP84 | *Ptr-bZIP5* | L58_SNP8 | *LncRNA locus 58* | V | AA | -0.92 | 9.14E-06 |
| L26_SNP42 | *LncRNA locus 26* | Ptr-C2H2-72_SNP33 | *Ptr-C2H2-72* | V | AA | 0.36 | 9.73E-06 |
| Ptr-PO17_SNP31 | *Ptr-PO17* | Ptr-COMT22_SNP58 | *Ptr-COMT22* | HC | AA | -15.20 | 9.84E-06 |
| Ptr-COMT25_SNP107 | *Ptr-COMT25* | L68_SNP1 | *LncRNA locus 68* | HC | DA | 21.80 | 9.94E-06 |
| L43_SNP59 | *LncRNA locus 43* | Ptr-4CL4_SNP22 | *Ptr-4CL4* | V | DD | 1.97 | 1.00E-05 |
| L09_SNP63 | *LncRNA locus 9* | Ptr-C4H3_SNP108 | *Ptr-C4H3* | HC | AA | -14.70 | 1.00E-05 |
| Ptr-PO40_SNP142 | *Ptr-PO40* | Ptr-PO70_SNP75 | *Ptr-PO70* | V | AD | 0.81 | 1.01E-05 |
| Ptr-bZIP5_SNP85 | *Ptr-bZIP5* | L58_SNP8 | *LncRNA locus 58* | V | AA | 0.90 | 1.02E-05 |
| Ptr-PO17_SNP20 | *Ptr-PO17* | Ptr-COMT22_SNP58 | *Ptr-COMT22* | HC | AA | 16.80 | 1.02E-05 |
| Ptr-bHLH21_SNP29 | *Ptr-bHLH21* | Ptr-HD-ZIP28_SNP56 | *Ptr-HD-ZIP28* | HC | AD | -29.00 | 1.03E-05 |
| Ptr-bHLH21_SNP29 | *Ptr-bHLH21* | Ptr-HD-ZIP28_SNP57 | *Ptr-HD-ZIP28* | HC | AD | -29.00 | 1.03E-05 |
| Ptr-PO25_SNP30 | *Ptr-PO25* | L25_SNP3 | *LncRNA locus 25* | V | DD | -1.06 | 1.04E-05 |
| Ptr-PO17_SNP20 | *Ptr-PO17* | Ptr-bHLH15_SNP56 | *Ptr-bHLH15* | V | AA | -0.31 | 1.04E-05 |
| L09_SNP63 | *LncRNA locus 9* | Ptr-HD-ZIP13_SNP18 | *Ptr-HD-ZIP13* | V | DD | -1.89 | 1.04E-05 |
| Pto-MiR475d_SNP7 | *Pto-MIR475d* | Ptr-LAC25_SNP39 | *Ptr-LAC25* | V | DD | -1.30 | 1.04E-05 |
| Ptr-HD-ZIP49_SNP69 | *Ptr-HD-ZIP49* | Ptr-bHLH23_SNP29 | *Ptr-bHLH23* | V | AD | 0.56 | 1.05E-05 |
| Ptr-HD-ZIP21_SNP40 | *Ptr-HD-ZIP21* | Ptr-PO70_SNP63 | *Ptr-PO70* | V | AD | 1.13 | 1.06E-05 |
| Ptr-CCR13_SNP7 | *Ptr-CCR13* | Ptr-4CL4_SNP72 | *Ptr-4CL4* | V | AD | 0.51 | 1.09E-05 |
| Ptr-COMT25_SNP107 | *Ptr-COMT25* | Ptr-HD-ZIP27_SNP78 | *Ptr-HD-ZIP27* | V | DA | -1.05 | 1.11E-05 |
| Ptr-CCR13_SNP7 | *Ptr-CCR13* | Ptr-4CL4_SNP70 | *Ptr-4CL4* | V | AD | 0.51 | 1.12E-05 |
| Ptr-CCR13_SNP7 | *Ptr-CCR13* | Ptr-4CL4_SNP71 | *Ptr-4CL4* | V | AD | 0.51 | 1.12E-05 |
| Ptr-PAL3_SNP100 | *Ptr-PAL3* | Ptr-PO82_SNP87 | *Ptr-PO82* | V | DD | -1.84 | 1.13E-05 |
| L25_SNP2 | *LncRNA locus 25* | L27_SNP13 | *LncRNA locus 27* | HC | DA | 10.50 | 1.14E-05 |
| Ptr-COMT25_SNP110 | *Ptr-COMT25* | Ptr-HD-ZIP27_SNP78 | *Ptr-HD-ZIP27* | V | DA | -1.06 | 1.14E-05 |
| Ptr-AT-Hook2_SNP90 | *Ptr-AT-Hook2* | Ptr-bHLH15_SNP57 | *Ptr-bHLH15* | HC | DA | -14.70 | 1.15E-05 |
| L32_SNP19 | *LncRNA locus 32* | Ptr-C2H2-77_SNP57 | *Ptr-C2H2-77* | HC | AA | -8.92 | 1.15E-05 |
| Ptr-HCT6_SNP25 | *Ptr-HCT6* | Ptr-COMT22_SNP58 | *Ptr-COMT22* | HC | AA | -16.90 | 1.16E-05 |
| Ptr-COMT25_SNP109 | *Ptr-COMT25* | Ptr-HD-ZIP27_SNP78 | *Ptr-HD-ZIP27* | V | DA | -1.07 | 1.16E-05 |
| Ptr-PO41_SNP52 | *Ptr-PO41* | Pto-MiR167d_SNP42 | *Pto-MIR167d* | V | DD | 1.86 | 1.21E-05 |
| Pto-MiR6446_SNP42 | *Pto-MIR6446* | Ptr-4CL4_SNP69 | *Ptr-4CL4* | V | AA | -1.04 | 1.22E-05 |
| L27_SNP13 | *LncRNA locus 27* | Ptr-LAC9_SNP101 | *Ptr-LAC9* | V | DA | -0.58 | 1.23E-05 |
| Ptr-HD-ZIP55_SNP30 | *Ptr-HD-ZIP55* | Ptr-HD-ZIP28_SNP21 | *Ptr-HD-ZIP28* | V | DA | 0.67 | 1.24E-05 |
| Ptr-CCoAOMT4_SNP39 | *Ptr-CCoAOMT4* | Ptr-PO70_SNP29 | *Ptr-PO70* | V | AA | -1.03 | 1.24E-05 |
| Ptr-HD-ZIP13_SNP9 | *Ptr-HD-ZIP13* | Ptr-bHLH17_SNP5 | *Ptr-bHLH17* | DBH | AA | 12.70 | 1.29E-05 |
| Ptr-HCT12_SNP8 | *Ptr-HCT12* | Ptr-C2H2-77_SNP57 | *Ptr-C2H2-77* | HC | AA | -15.40 | 1.29E-05 |
| L49_SNP33 | *LncRNA locus 49* | Ptr-HD-ZIP55_SNP30 | *Ptr-HD-ZIP55* | V | DA | -1.23 | 1.30E-05 |
| L49_SNP34 | *LncRNA locus 49* | Ptr-HD-ZIP55_SNP30 | *Ptr-HD-ZIP55* | V | DA | -1.23 | 1.30E-05 |
| Ptr-bZIP5_SNP69 | *Ptr-bZIP5* | L58_SNP7 | *LncRNA locus 58* | V | DA | -1.27 | 1.31E-05 |
| Ptr-HCT12_SNP9 | *Ptr-HCT12* | Ptr-4CL4_SNP22 | *Ptr-4CL4* | V | AD | -1.45 | 1.31E-05 |
| Ptr-HD-ZIP13_SNP6 | *Ptr-HD-ZIP13* | Ptr-bHLH17_SNP5 | *Ptr-bHLH17* | DBH | AA | 14.50 | 1.31E-05 |
| Ptr-C3H2_SNP39 | *Ptr-C3H2* | Ptr-CCR28_SNP94 | *Ptr-CCR28* | FW | DD | 9.93 | 1.32E-05 |
| Ptr-CCR13_SNP7 | *Ptr-CCR13* | Ptr-PO82_SNP71 | *Ptr-PO82* | V | DA | 0.53 | 1.33E-05 |
| L19_SNP19 | *LncRNA locus 19* | Ptr-bHLH4_SNP44 | *Ptr-bHLH4* | HC | AD | 20.60 | 1.36E-05 |
| L19_SNP19 | *LncRNA locus 19* | Ptr-bHLH4_SNP45 | *Ptr-bHLH4* | HC | AD | 20.60 | 1.36E-05 |
| Ptr-AT-Hook2_SNP90 | *Ptr-AT-Hook2* | Ptr-PO40_SNP142 | *Ptr-PO40* | V | AD | 0.97 | 1.37E-05 |
| Ptr-HD-ZIP24_SNP81 | *Ptr-HD-ZIP24* | Ptr-PO70_SNP63 | *Ptr-PO70* | V | AD | -1.12 | 1.37E-05 |
| L06_SNP61 | *LncRNA locus 6* | Ptr-4CL4_SNP26 | *Ptr-4CL4* | V | DA | -0.74 | 1.39E-05 |
| Ptr-PO40_SNP142 | *Ptr-PO40* | Ptr-HD-ZIP28_SNP21 | *Ptr-HD-ZIP28* | V | AD | 0.69 | 1.39E-05 |
| Ptr-HD-ZIP13_SNP2 | *Ptr-HD-ZIP13* | Ptr-PO40_SNP145 | *Ptr-PO40* | HC | DA | -20.70 | 1.39E-05 |
| Ptr-bHLH15_SNP56 | *Ptr-bHLH15* | Ptr-bHLH18_SNP45 | *Ptr-bHLH18* | V | AA | -0.28 | 1.42E-05 |
| Ptr-HD-ZIP13_SNP18 | *Ptr-HD-ZIP13* | Ptr-CAD6_SNP10 | *Ptr-CAD6* | V | DD | -1.46 | 1.42E-05 |
| Ptr-CAD10_SNP77 | *Ptr-CAD10* | Ptr-bHLH13_SNP39 | *Ptr-bHLH13* | HC | AA | 13.40 | 1.43E-05 |
| L15_SNP14 | *LncRNA locus 15* | Ptr-COMT22_SNP58 | *Ptr-COMT22* | HC | AA | 17.40 | 1.45E-05 |
| Ptr-COMT25_SNP150 | *Ptr-COMT25* | Ptr-COMT22_SNP58 | *Ptr-COMT22* | HC | DA | 15.10 | 1.45E-05 |
| Ptr-CCR13_SNP7 | *Ptr-CCR13* | Ptr-PO82_SNP30 | *Ptr-PO82* | V | DA | 0.39 | 1.46E-05 |
| Ptr-LAC17_SNP60 | *Ptr-LAC17* | Ptr-PO85_SNP54 | *Ptr-PO85* | V | AA | 0.19 | 1.46E-05 |
| Ptr-bHLH15_SNP56 | *Ptr-bHLH15* | Ptr-HD-ZIP49_SNP69 | *Ptr-HD-ZIP49* | V | DA | 0.53 | 1.47E-05 |
| Pto-MiR167a_SNP20 | *Pto-MIR167a* | Ptr-Myb/SANT2_SNP21 | *Ptr-Myb/SANT2* | HEC | AD | 19.80 | 1.47E-05 |
| Ptr-COMT25_SNP110 | *Ptr-COMT25* | Ptr-PO72_SNP18 | *Ptr-PO72* | HC | DA | 13.10 | 1.48E-05 |
| Ptr-LAC4_SNP49 | *Ptr-LAC4* | L19_SNP31 | *LncRNA locus 19* | HC | DA | -29.30 | 1.49E-05 |
| Ptr-LAC4_SNP50 | *Ptr-LAC4* | L19_SNP31 | *LncRNA locus 19* | HC | DA | -29.30 | 1.49E-05 |
| Ptr-HD-ZIP10_SNP6 | *Ptr-HD-ZIP10* | Ptr-HCT7_SNP9 | *Ptr-HCT7* | V | DD | -1.49 | 1.53E-05 |
| Ptr-PO70_SNP63 | *Ptr-PO70* | Ptr-PO85_SNP50 | *Ptr-PO85* | V | DA | -1.30 | 1.53E-05 |
| Ptr-PO70_SNP63 | *Ptr-PO70* | Ptr-HD-ZIP55_SNP30 | *Ptr-HD-ZIP55* | V | DA | -1.21 | 1.54E-05 |
| L06_SNP155 | *LncRNA locus 6* | Ptr-Myb/SANT2_SNP21 | *Ptr-Myb/SANT2* | HEC | DD | -28.40 | 1.54E-05 |
| Ptr-HCT1_SNP23 | *Ptr-HCT1* | Ptr-C2H2-72_SNP33 | *Ptr-C2H2-72* | V | AA | -0.65 | 1.55E-05 |
| Ptr-COMT25_SNP110 | *Ptr-COMT25* | Ptr-PO72_SNP1 | *Ptr-PO72* | HC | DA | -21.30 | 1.55E-05 |
| Ptr-COMT25_SNP104 | *Ptr-COMT25* | Ptr-PO72_SNP18 | *Ptr-PO72* | HC | DA | 13.70 | 1.56E-05 |
| Ptr-HD-ZIP35_SNP44 | *Ptr-HD-ZIP35* | Ptr-PO70_SNP63 | *Ptr-PO70* | V | AD | -1.10 | 1.58E-05 |
| L33_SNP4 | *LncRNA locus 33* | L58_SNP7 | *LncRNA locus 58* | V | AA | -0.89 | 1.59E-05 |
| Ptr-CAD7_SNP9 | *Ptr-CAD7* | Ptr-C3H2_SNP39 | *Ptr-C3H2* | V | DA | 1.28 | 1.59E-05 |
| Ptr-LAC17_SNP60 | *Ptr-LAC17* | Ptr-PO70_SNP63 | *Ptr-PO70* | V | AD | -1.12 | 1.61E-05 |
| Ptr-LAC4_SNP49 | *Ptr-LAC4* | L58_SNP8 | *LncRNA locus 58* | V | AA | 0.94 | 1.63E-05 |
| Ptr-LAC4_SNP50 | *Ptr-LAC4* | L58_SNP8 | *LncRNA locus 58* | V | AA | -0.94 | 1.63E-05 |
| Ptr-PAL3_SNP100 | *Ptr-PAL3* | Ptr-CCR9_SNP20 | *Ptr-CCR9* | V | AA | -0.48 | 1.63E-05 |
| Ptr-HCT8_SNP80 | *Ptr-HCT8* | Ptr-PO35_SNP1 | *Ptr-PO35* | V | DD | 1.83 | 1.63E-05 |
| Ptr-CCR13_SNP7 | *Ptr-CCR13* | L19_SNP19 | *LncRNA locus 19* | FW | AD | -2.60 | 1.64E-05 |
| Ptr-COMT25_SNP173 | *Ptr-COMT25* | Ptr-HD-ZIP55_SNP2 | *Ptr-HD-ZIP55* | V | DD | -1.23 | 1.65E-05 |
| L62_SNP113 | *LncRNA locus 62* | Ptr-PO52_SNP37 | *Ptr-PO52* | CC | AD | 22.90 | 1.67E-05 |
| Ptr-CCR13_SNP7 | *Ptr-CCR13* | L19_SNP32 | *LncRNA locus 19* | DBH | DA | -8.32 | 1.68E-05 |
| Ptr-LAC4_SNP49 | *Ptr-LAC4* | Ptr-C3H3_SNP7 | *Ptr-C3H3* | HC | DA | 27.10 | 1.72E-05 |
| Ptr-LAC4_SNP50 | *Ptr-LAC4* | Ptr-C3H3_SNP7 | *Ptr-C3H3* | HC | DA | 27.10 | 1.72E-05 |
| L33_SNP4 | *LncRNA locus 33* | L58_SNP8 | *LncRNA locus 58* | V | AA | -0.87 | 1.75E-05 |
| Ptr-bHLH18_SNP21 | *Ptr-bHLH18* | Ptr-C4H3_SNP108 | *Ptr-C4H3* | HC | DA | 8.41 | 1.75E-05 |
| Ptr-CCoAOMT4_SNP40 | *Ptr-CCoAOMT4* | Ptr-PO70_SNP29 | *Ptr-PO70* | V | AA | -0.99 | 1.76E-05 |
| Ptr-PO5_SNP22 | *Ptr-PO5* | Ptr-bZIP5_SNP104 | *Ptr-bZIP5* | V | DD | 0.96 | 1.78E-05 |
| Ptr-bHLH2_SNP16 | *Ptr-bHLH2* | Ptr-PO40_SNP145 | *Ptr-PO40* | HC | DA | 15.20 | 1.78E-05 |
| Ptr-COMT25_SNP4 | *Ptr-COMT25* | L19_SNP8 | *LncRNA locus 19* | HC | AD | 24.00 | 1.80E-05 |
| L09_SNP26 | *LncRNA locus 9* | Ptr-LAC22_SNP102 | *Ptr-LAC22* | DBH | DA | -8.12 | 1.80E-05 |
| L10_SNP110 | *LncRNA locus 10* | Ptr-COMT12_SNP120 | *Ptr-COMT12* | HC | AA | 19.60 | 1.81E-05 |
| Ptr-4CL20_SNP97 | *Ptr-4CL20* | Ptr-COMT22_SNP58 | *Ptr-COMT22* | HC | AA | -17.20 | 1.82E-05 |
| Ptr-CAD10_SNP214 | *Ptr-CAD10* | Ptr-PO23_SNP8 | *Ptr-PO23* | HC | DA | -17.90 | 1.82E-05 |
| Ptr-CCR13_SNP7 | *Ptr-CCR13* | L19_SNP39 | *LncRNA locus 19* | FW | AD | -2.64 | 1.83E-05 |
| Ptr-PO33_SNP41 | *Ptr-PO33* | Ptr-PO70_SNP3 | *Ptr-PO70* | V | AA | -1.13 | 1.84E-05 |
| Ptr-CAD10_SNP184 | *Ptr-CAD10* | Ptr-bHLH14_SNP20 | *Ptr-bHLH14* | MFA | DD | -11.30 | 1.85E-05 |
| Ptr-4CL20_SNP51 | *Ptr-4CL20* | Ptr-bHLH15_SNP57 | *Ptr-bHLH15* | HC | AA | -7.45 | 1.86E-05 |
| Ptr-CAD10_SNP69 | *Ptr-CAD10* | Ptr-bHLH13_SNP39 | *Ptr-bHLH13* | HC | DA | 21.40 | 1.90E-05 |
| L19_SNP10 | *LncRNA locus 19* | Ptr-PO54_SNP54 | *Ptr-PO54* | LC | DD | -6.93 | 1.90E-05 |
| L25_SNP2 | *LncRNA locus 25* | L32_SNP111 | *LncRNA locus 32* | HC | AD | -21.00 | 1.93E-05 |
| Ptr-HCT1_SNP22 | *Ptr-HCT1* | Ptr-bZIP5_SNP84 | *Ptr-bZIP5* | V | AA | -0.72 | 1.95E-05 |
| Ptr-PO5_SNP22 | *Ptr-PO5* | Ptr-PO40_SNP145 | *Ptr-PO40* | V | AA | -0.41 | 1.96E-05 |
| L19_SNP31 | *LncRNA locus 19* | Ptr-AT-Hook2_SNP2 | *Ptr-AT-Hook2* | HC | AD | -34.60 | 1.97E-05 |
| Ptr-PO33_SNP41 | *Ptr-PO33* | Ptr-PO70_SNP29 | *Ptr-PO70* | V | AA | -1.14 | 2.01E-05 |
| L40_SNP2 | *LncRNA locus 40* | Ptr-TBP1_SNP17 | *Ptr-TBP1* | V | DA | -1.13 | 2.01E-05 |
| Ptr-4CL20_SNP112 | *Ptr-4CL20* | Ptr-4CL4_SNP71 | *Ptr-4CL4* | CC | AD | 22.60 | 2.03E-05 |
| Ptr-PAL3_SNP117 | *Ptr-PAL3* | Ptr-bHLH11_SNP44 | *Ptr-bHLH11* | CC | AD | 22.60 | 2.03E-05 |
| Ptr-HCT1_SNP20 | *Ptr-HCT1* | Ptr-PO70_SNP63 | *Ptr-PO70* | HC | AA | 21.40 | 2.04E-05 |
| Ptr-COMT25_SNP111 | *Ptr-COMT25* | Ptr-HD-ZIP27_SNP78 | *Ptr-HD-ZIP27* | V | DA | -1.05 | 2.06E-05 |
| Ptr-4CL17_SNP5 | *Ptr-4CL17* | Ptr-PO70_SNP64 | *Ptr-PO70* | FW | AD | -3.44 | 2.10E-05 |
| Ptr-PO22_SNP64 | *Ptr-PO22* | Ptr-PO70_SNP75 | *Ptr-PO70* | V | AA | 0.35 | 2.11E-05 |
| Ptr-PAL3_SNP107 | *Ptr-PAL3* | Ptr-HCT8_SNP58 | *Ptr-HCT8* | HC | AD | 20.00 | 2.13E-05 |
| Ptr-PAL3_SNP102 | *Ptr-PAL3* | L15_SNP48 | *LncRNA locus 15* | V | AA | -1.12 | 2.15E-05 |
| Ptr-PO23_SNP8 | *Ptr-PO23* | Ptr-CCR33_SNP25 | *Ptr-CCR33* | HC | AA | 13.00 | 2.15E-05 |
| Ptr-CCR9_SNP119 | *Ptr-CCR9* | Ptr-COMT22_SNP58 | *Ptr-COMT22* | CC | AA | -15.40 | 2.16E-05 |
| Ptr-COMT25_SNP109 | *Ptr-COMT25* | Ptr-PO72_SNP1 | *Ptr-PO72* | HC | DA | -21.20 | 2.16E-05 |
| Ptr-CAD14_SNP81 | *Ptr-CAD14* | L50_SNP110 | *LncRNA locus 50* | V | DA | 1.57 | 2.17E-05 |
| Ptr-PO25_SNP30 | *Ptr-PO25* | Ptr-C2H2-72_SNP33 | *Ptr-C2H2-72* | V | DA | -0.65 | 2.18E-05 |
| Ptr-HCT6_SNP29 | *Ptr-HCT6* | Ptr-COMT22_SNP58 | *Ptr-COMT22* | HC | AA | 20.10 | 2.18E-05 |
| Ptr-HD-ZIP4_SNP11 | *Ptr-HD-ZIP4* | Ptr-C4H3_SNP8 | *Ptr-C4H3* | V | AD | -0.85 | 2.20E-05 |
| Ptr-HCT8_SNP80 | *Ptr-HCT8* | Ptr-PO85_SNP50 | *Ptr-PO85* | V | DD | -1.00 | 2.21E-05 |
| L19_SNP138 | *LncRNA locus 19* | Ptr-4CL4_SNP70 | *Ptr-4CL4* | V | DA | 0.72 | 2.23E-05 |
| L19_SNP138 | *LncRNA locus 19* | Ptr-4CL4_SNP71 | *Ptr-4CL4* | V | DA | 0.72 | 2.23E-05 |
| Ptr-CAD10_SNP187 | *Ptr-CAD10* | Ptr-PO85_SNP50 | *Ptr-PO85* | V | DD | -1.05 | 2.23E-05 |
| Ptr-bHLH11_SNP44 | *Ptr-bHLH11* | L62_SNP113 | *LncRNA locus 62* | CC | AD | 15.60 | 2.25E-05 |
| Ptr-CCR10_SNP7 | *Ptr-CCR10* | Ptr-bZIP4_SNP135 | *Ptr-bZIP4* | FW | DA | 3.95 | 2.25E-05 |
| Ptr-HCT6_SNP51 | *Ptr-HCT6* | L25_SNP3 | *LncRNA locus 25* | HEC | DA | -15.60 | 2.27E-05 |
| L40_SNP2 | *LncRNA locus 40* | Ptr-PO72_SNP40 | *Ptr-PO72* | V | DA | -1.19 | 2.27E-05 |
| Ptr-HD-ZIP27_SNP78 | *Ptr-HD-ZIP27* | Ptr-HCT12_SNP87 | *Ptr-HCT12* | V | AD | -1.10 | 2.28E-05 |
| Ptr-CAD10_SNP184 | *Ptr-CAD10* | Ptr-PO85_SNP50 | *Ptr-PO85* | V | DD | -1.02 | 2.30E-05 |
| Ptr-PO25_SNP30 | *Ptr-PO25* | Ptr-HD-ZIP21_SNP40 | *Ptr-HD-ZIP21* | V | DA | -0.43 | 2.31E-05 |
| Ptr-4CL20_SNP115 | *Ptr-4CL20* | L49_SNP33 | *LncRNA locus 49* | V | AD | -1.29 | 2.32E-05 |
| Ptr-4CL20_SNP115 | *Ptr-4CL20* | L49_SNP34 | *LncRNA locus 49* | V | AD | -1.29 | 2.32E-05 |
| Ptr-COMT25_SNP95 | *Ptr-COMT25* | Ptr-HCT13_SNP37 | *Ptr-HCT13* | DBH | AA | -6.99 | 2.33E-05 |
| Ptr-HD-ZIP10_SNP6 | *Ptr-HD-ZIP10* | L54_SNP10 | *LncRNA locus 54* | FL | AA | 0.09 | 2.34E-05 |
| Pto-MiR167a_SNP41 | *Pto-MIR167a* | Ptr-LAC43_SNP28 | *Ptr-LAC43* | HC | AA | -13.10 | 2.35E-05 |
| L19_SNP77 | *LncRNA locus 19* | Ptr-COMT12_SNP122 | *Ptr-COMT12* | V | DD | 1.92 | 2.36E-05 |
| Ptr-COMT22_SNP58 | *Ptr-COMT22* | Ptr-COMT1_SNP16 | *Ptr-COMT1* | HC | DA | -22.20 | 2.37E-05 |
| Ptr-PO23_SNP8 | *Ptr-PO23* | Ptr-HCT2_SNP9 | *Ptr-HCT2* | HC | AA | -13.80 | 2.37E-05 |
| Ptr-PO19_SNP86 | *Ptr-PO19* | L49_SNP33 | *LncRNA locus 49* | DBH | AD | 19.30 | 2.42E-05 |
| Ptr-PO19_SNP86 | *Ptr-PO19* | L49_SNP34 | *LncRNA locus 49* | DBH | AD | 19.30 | 2.42E-05 |
| Ptr-CAD10_SNP184 | *Ptr-CAD10* | Ptr-bHLH15_SNP56 | *Ptr-bHLH15* | DBH | DA | -4.89 | 2.44E-05 |
| Ptr-C3H3_SNP17 | *Ptr-C3H3* | Ptr-Myb/SANT2_SNP21 | *Ptr-Myb/SANT2* | HEC | DD | -28.70 | 2.51E-05 |
| Ptr-PO40_SNP142 | *Ptr-PO40* | L43_SNP51 | *LncRNA locus 43* | V | DD | -0.88 | 2.53E-05 |
| L40_SNP2 | *LncRNA locus 40* | L50_SNP108 | *LncRNA locus 50* | V | DA | -1.40 | 2.54E-05 |
| Ptr-HCT13_SNP37 | *Ptr-HCT13* | Ptr-PO70_SNP63 | *Ptr-PO70* | V | AD | 1.07 | 2.54E-05 |
| Ptr-CAD13_SNP44 | *Ptr-CAD13* | Pto-MiR396g_SNP2 | *Pto-MIR396g* | HC | AA | -18.20 | 2.55E-05 |
| Ptr-bHLH13_SNP39 | *Ptr-bHLH13* | L49_SNP33 | *LncRNA locus 49* | DBH | DD | -27.90 | 2.59E-05 |
| Ptr-bHLH13_SNP39 | *Ptr-bHLH13* | L49_SNP34 | *LncRNA locus 49* | DBH | DD | -27.90 | 2.59E-05 |
| L13_SNP23 | *LncRNA locus 13* | Ptr-HD-ZIP21_SNP40 | *Ptr-HD-ZIP21* | V | AA | 0.58 | 2.59E-05 |
| Ptr-PAL3_SNP92 | *Ptr-PAL3* | L19_SNP18 | *LncRNA locus 19* | HC | AD | -26.00 | 2.61E-05 |
| Ptr-COMT9_SNP21 | *Ptr-COMT9* | Ptr-LAC31_SNP6 | *Ptr-LAC31* | HC | AA | -19.80 | 2.62E-05 |
| Ptr-C2H2-72_SNP33 | *Ptr-C2H2-72* | L70_SNP8 | *LncRNA locus 70* | V | AA | -0.77 | 2.63E-05 |
| Ptr-CAD14_SNP81 | *Ptr-CAD14* | Ptr-bHLH23_SNP29 | *Ptr-bHLH23* | V | DA | 1.13 | 2.68E-05 |
| L19_SNP19 | *LncRNA locus 19* | Ptr-bHLH4_SNP42 | *Ptr-bHLH4* | HC | AD | 19.80 | 2.68E-05 |
| Ptr-PO40_SNP142 | *Ptr-PO40* | Ptr-4CL20_SNP97 | *Ptr-4CL20* | V | DA | 1.08 | 2.71E-05 |
| Ptr-CAD14_SNP81 | *Ptr-CAD14* | Ptr-C4H3_SNP8 | *Ptr-C4H3* | V | DA | 1.18 | 2.74E-05 |
| Ptr-COMT25_SNP152 | *Ptr-COMT25* | Ptr-COMT22_SNP58 | *Ptr-COMT22* | HC | DA | 14.30 | 2.76E-05 |
| Ptr-HCT7_SNP9 | *Ptr-HCT7* | Ptr-C2H2-77_SNP70 | *Ptr-C2H2-77* | V | DA | 0.50 | 2.77E-05 |
| L33_SNP29 | *LncRNA locus 33* | Ptr-CCR9_SNP130 | *Ptr-CCR9* | LC | AA | -2.73 | 2.78E-05 |
| L19_SNP23 | *LncRNA locus 19* | Ptr-COMT22_SNP58 | *Ptr-COMT22* | HC | AA | 14.90 | 2.78E-05 |
| Ptr-PO40_SNP142 | *Ptr-PO40* | L49_SNP33 | *LncRNA locus 49* | V | AD | -1.46 | 2.80E-05 |
| Ptr-PO40_SNP142 | *Ptr-PO40* | L49_SNP34 | *LncRNA locus 49* | V | AD | -1.46 | 2.80E-05 |
| Ptr-bZIP5_SNP84 | *Ptr-bZIP5* | L58_SNP7 | *LncRNA locus 58* | V | AA | -0.88 | 2.80E-05 |
| L19_SNP138 | *LncRNA locus 19* | Ptr-4CL4_SNP72 | *Ptr-4CL4* | V | DA | -0.71 | 2.80E-05 |
| Ptr-HD-ZIP21_SNP40 | *Ptr-HD-ZIP21* | Ptr-PO82_SNP87 | *Ptr-PO82* | V | AD | 1.00 | 2.80E-05 |
| Ptr-COMT25_SNP111 | *Ptr-COMT25* | Ptr-HD-ZIP24_SNP81 | *Ptr-HD-ZIP24* | DBH | AA | -5.00 | 2.81E-05 |
| Ptr-C2H2-77_SNP57 | *Ptr-C2H2-77* | Ptr-C4H3_SNP108 | *Ptr-C4H3* | HC | AA | 12.80 | 2.86E-05 |
| L37_SNP35 | *LncRNA locus 37* | Ptr-C4H3_SNP8 | *Ptr-C4H3* | V | AA | 0.22 | 2.87E-05 |
| Ptr-PO22_SNP64 | *Ptr-PO22* | Ptr-Myb/SANT2_SNP21 | *Ptr-Myb/SANT2* | HEC | AD | 20.20 | 2.88E-05 |
| Ptr-PAL3_SNP92 | *Ptr-PAL3* | L19_SNP16 | *LncRNA locus 19* | HC | AD | -25.80 | 2.89E-05 |
| L19_SNP138 | *LncRNA locus 19* | Ptr-HD-ZIP21_SNP40 | *Ptr-HD-ZIP21* | DBH | DA | -4.50 | 2.91E-05 |
| Ptr-PAL3_SNP117 | *Ptr-PAL3* | Ptr-PO52_SNP37 | *Ptr-PO52* | CC | AD | -18.20 | 2.93E-05 |
| Ptr-HD-ZIP4_SNP11 | *Ptr-HD-ZIP4* | Ptr-PO70_SNP75 | *Ptr-PO70* | V | AD | -0.40 | 2.99E-05 |
| Ptr-CCR9_SNP7 | *Ptr-CCR9* | Ptr-HD-ZIP49_SNP69 | *Ptr-HD-ZIP49* | HC | DD | -44.70 | 3.01E-05 |
| Ptr-4CL14_SNP43 | *Ptr-4CL14* | Ptr-Myb/SANT2_SNP21 | *Ptr-Myb/SANT2* | HEC | DD | -26.70 | 3.01E-05 |
| Ptr-HCT1_SNP23 | *Ptr-HCT1* | Ptr-4CL4_SNP22 | *Ptr-4CL4* | V | DD | -1.41 | 3.06E-05 |
| Ptr-bZIP5_SNP85 | *Ptr-bZIP5* | L58_SNP7 | *LncRNA locus 58* | V | AA | 0.86 | 3.07E-05 |
| Ptr-PAL3_SNP92 | *Ptr-PAL3* | L19_SNP67 | *LncRNA locus 19* | HC | AA | -11.10 | 3.08E-05 |
| Ptr-HCT1_SNP10 | *Ptr-HCT1* | L19_SNP77 | *LncRNA locus 19* | V | DD | -1.25 | 3.08E-05 |
| Ptr-HD-ZIP27_SNP62 | *Ptr-HD-ZIP27* | Pto-MiR6446_SNP42 | *Pto-MIR6446* | V | AA | -0.59 | 3.09E-05 |
| Ptr-HD-ZIP27_SNP63 | *Ptr-HD-ZIP27* | Pto-MiR6446_SNP42 | *Pto-MIR6446* | V | AA | -0.59 | 3.09E-05 |
| Ptr-CCR13_SNP7 | *Ptr-CCR13* | L19_SNP32 | *LncRNA locus 19* | FW | AD | -2.66 | 3.10E-05 |
| Ptr-COMT25_SNP111 | *Ptr-COMT25* | Ptr-Myb/SANT2_SNP21 | *Ptr-Myb/SANT2* | HEC | AD | 20.20 | 3.13E-05 |
| Ptr-COMT25_SNP111 | *Ptr-COMT25* | Ptr-HCT13_SNP37 | *Ptr-HCT13* | DBH | AA | 4.99 | 3.15E-05 |
| Ptr-HD-ZIP49_SNP84 | *Ptr-HD-ZIP49* | Ptr-HD-ZIP28_SNP21 | *Ptr-HD-ZIP28* | H | AA | -3.11 | 3.16E-05 |
| Ptr-PO67_SNP45 | *Ptr-PO67* | Ptr-C4H3_SNP108 | *Ptr-C4H3* | HC | AA | -10.60 | 3.27E-05 |
| Ptr-HD-ZIP49_SNP64 | *Ptr-HD-ZIP49* | Ptr-PO85_SNP1 | *Ptr-PO85* | HC | DA | -12.00 | 3.27E-05 |
| Ptr-HD-ZIP13_SNP18 | *Ptr-HD-ZIP13* | L13_SNP23 | *LncRNA locus 13* | V | DA | 1.37 | 3.28E-05 |
| Ptr-COMT25_SNP111 | *Ptr-COMT25* | Ptr-HD-ZIP49_SNP69 | *Ptr-HD-ZIP49* | V | AA | -0.29 | 3.29E-05 |
| Pto-MiR167a_SNP20 | *Pto-MIR167a* | Ptr-PO41_SNP35 | *Ptr-PO41* | V | DD | -0.93 | 3.30E-05 |
| Ptr-PO28_SNP80 | *Ptr-PO28* | L25_SNP2 | *LncRNA locus 25* | FW | DA | -4.08 | 3.31E-05 |
| L49_SNP51 | *LncRNA locus 49* | Ptr-LAC2_SNP80 | *Ptr-LAC2* | DBH | DA | -9.65 | 3.33E-05 |
| L40_SNP2 | *LncRNA locus 40* | L50_SNP110 | *LncRNA locus 50* | V | DA | 1.52 | 3.34E-05 |
| Ptr-4CL3_SNP44 | *Ptr-4CL3* | Ptr-COMT25_SNP4 | *Ptr-COMT25* | HC | AA | -20.50 | 3.38E-05 |
| Ptr-PO17_SNP20 | *Ptr-PO17* | Ptr-Myb/SANT2_SNP21 | *Ptr-Myb/SANT2* | HEC | DD | -25.70 | 3.39E-05 |
| Ptr-HD-ZIP13_SNP6 | *Ptr-HD-ZIP13* | L37_SNP18 | *LncRNA locus 37* | V | DA | -0.87 | 3.40E-05 |
| Ptr-COMT25_SNP109 | *Ptr-COMT25* | L19_SNP77 | *LncRNA locus 19* | V | DD | 1.65 | 3.43E-05 |
| Ptr-COMT25_SNP110 | *Ptr-COMT25* | L19_SNP77 | *LncRNA locus 19* | V | DD | 1.65 | 3.43E-05 |
| L40_SNP2 | *LncRNA locus 40* | Ptr-COMT12_SNP122 | *Ptr-COMT12* | V | DD | 1.76 | 3.48E-05 |
| Ptr-CCR13_SNP39 | *Ptr-CCR13* | Ptr-COMT22_SNP58 | *Ptr-COMT22* | HC | AA | -17.40 | 3.49E-05 |
| L50_SNP108 | *LncRNA locus 50* | Ptr-PO85_SNP1 | *Ptr-PO85* | HC | AA | 5.80 | 3.49E-05 |
| Ptr-C2H2-2_SNP5 | *Ptr-C2H2-2* | Ptr-HCT12_SNP87 | *Ptr-HCT12* | HC | AA | -13.20 | 3.53E-05 |
| Ptr-HD-ZIP27_SNP86 | *Ptr-HD-ZIP27* | Ptr-C2H2-72_SNP33 | *Ptr-C2H2-72* | V | AA | 0.87 | 3.54E-05 |
| Ptr-LAC4_SNP49 | *Ptr-LAC4* | L58_SNP7 | *LncRNA locus 58* | V | AA | 0.91 | 3.57E-05 |
| Ptr-LAC4_SNP50 | *Ptr-LAC4* | L58_SNP7 | *LncRNA locus 58* | V | AA | -0.91 | 3.57E-05 |
| Ptr-HD-ZIP13_SNP6 | *Ptr-HD-ZIP13* | Ptr-bHLH17_SNP5 | *Ptr-bHLH17* | V | AA | 0.98 | 3.61E-05 |
| Ptr-CAD10_SNP198 | *Ptr-CAD10* | Ptr-PAL3_SNP83 | *Ptr-PAL3* | HC | DA | -15.50 | 3.63E-05 |
| L19_SNP67 | *LncRNA locus 19* | Ptr-COMT22_SNP58 | *Ptr-COMT22* | HC | AA | -15.00 | 3.64E-05 |
| L19_SNP68 | *LncRNA locus 19* | Ptr-COMT22_SNP58 | *Ptr-COMT22* | HC | AA | -15.00 | 3.64E-05 |
| Ptr-PAL3_SNP102 | *Ptr-PAL3* | Ptr-PO17_SNP55 | *Ptr-PO17* | V | AA | -0.98 | 3.65E-05 |
| Ptr-PO33_SNP41 | *Ptr-PO33* | Ptr-PO72_SNP40 | *Ptr-PO72* | V | AA | -1.11 | 3.68E-05 |
| Ptr-CCR13_SNP7 | *Ptr-CCR13* | L19_SNP33 | *LncRNA locus 19* | DBH | DA | 8.07 | 3.69E-05 |
| Ptr-COMT25_SNP107 | *Ptr-COMT25* | Ptr-HCT13_SNP37 | *Ptr-HCT13* | DBH | AA | -4.83 | 3.70E-05 |
| Ptr-COMT25_SNP110 | *Ptr-COMT25* | Ptr-PO70_SNP19 | *Ptr-PO70* | HC | DD | 27.40 | 3.77E-05 |
| Ptr-CCoAOMT3_SNP44 | *Ptr-CCoAOMT3* | Ptr-COMT22_SNP58 | *Ptr-COMT22* | HC | AA | 19.00 | 3.78E-05 |
| Pto-MiR396g_SNP22 | *Pto-MIR396g* | Ptr-LAC25_SNP10 | *Ptr-LAC25* | V | AA | -0.40 | 3.80E-05 |
| L30_SNP18 | *LncRNA locus 30* | Ptr-HD-ZIP55_SNP2 | *Ptr-HD-ZIP55* | HC | AA | 12.80 | 3.82E-05 |
| Ptr-PAL3_SNP83 | *Ptr-PAL3* | Ptr-CCR9_SNP130 | *Ptr-CCR9* | HC | AA | -7.80 | 3.83E-05 |
| L13_SNP121 | *LncRNA locus 13* | Ptr-HD-ZIP49_SNP64 | *Ptr-HD-ZIP49* | HC | DA | -12.40 | 3.84E-05 |
| Ptr-PO31_SNP8 | *Ptr-PO31* | Ptr-bHLH11_SNP44 | *Ptr-bHLH11* | HC | AA | 6.68 | 3.85E-05 |
| Ptr-PO33_SNP41 | *Ptr-PO33* | Ptr-bHLH18_SNP21 | *Ptr-bHLH18* | V | AD | 1.37 | 3.89E-05 |
| Ptr-PO16_SNP11 | *Ptr-PO16* | Pto-MiR482c_SNP4 | *Pto-MIR482c* | V | DA | -1.01 | 3.90E-05 |
| Ptr-CCR13_SNP7 | *Ptr-CCR13* | L19_SNP33 | *LncRNA locus 19* | FW | AD | -2.56 | 3.92E-05 |
| Pto-MiR160e_SNP17 | *Pto-MIR160e* | Ptr-COMT22_SNP58 | *Ptr-COMT22* | HC | AD | -15.00 | 3.93E-05 |
| Ptr-PO17_SNP31 | *Ptr-PO17* | L58_SNP7 | *LncRNA locus 58* | HC | AD | -37.60 | 3.96E-05 |
| Ptr-COMT25_SNP109 | *Ptr-COMT25* | Ptr-PO70_SNP19 | *Ptr-PO70* | HC | DD | 27.30 | 3.97E-05 |
| L32_SNP87 | *LncRNA locus 32* | L50_SNP110 | *LncRNA locus 50* | HC | DD | -43.10 | 3.98E-05 |
| Ptr-PAL3_SNP91 | *Ptr-PAL3* | Ptr-COMT13_SNP43 | *Ptr-COMT13* | HC | DA | 31.80 | 3.98E-05 |
| Ptr-CCR13_SNP7 | *Ptr-CCR13* | Ptr-HD-ZIP48_SNP15 | *Ptr-HD-ZIP48* | V | DA | -0.61 | 3.98E-05 |
| L09_SNP30 | *LncRNA locus 9* | L35_SNP13 | *LncRNA locus 35* | V | AD | -0.68 | 3.99E-05 |
| Ptr-CCR28_SNP94 | *Ptr-CCR28* | Ptr-bZIP5_SNP85 | *Ptr-bZIP5* | FW | DA | 8.54 | 4.01E-05 |
| Ptr-PAL3_SNP100 | *Ptr-PAL3* | Ptr-CCR9_SNP19 | *Ptr-CCR9* | V | AA | 0.47 | 4.01E-05 |
| Ptr-CAD10_SNP214 | *Ptr-CAD10* | L10_SNP109 | *LncRNA locus 10* | HC | AA | -26.50 | 4.02E-05 |
| L25_SNP3 | *LncRNA locus 25* | L27_SNP13 | *LncRNA locus 27* | HC | DA | 9.60 | 4.02E-05 |
| Ptr-CAD10_SNP77 | *Ptr-CAD10* | L40_SNP2 | *LncRNA locus 40* | V | AD | 0.88 | 4.02E-05 |
| Ptr-CAD13_SNP97 | *Ptr-CAD13* | Ptr-C3H3_SNP7 | *Ptr-C3H3* | DBH | AA | 13.00 | 4.03E-05 |
| Ptr-CAD13_SNP98 | *Ptr-CAD13* | Ptr-C3H3_SNP7 | *Ptr-C3H3* | DBH | AA | -13.00 | 4.03E-05 |
| Ptr-C2H2-77_SNP57 | *Ptr-C2H2-77* | Ptr-C4H3_SNP108 | *Ptr-C4H3* | HC | DD | 29.80 | 4.06E-05 |
| Ptr-HD-ZIP27_SNP62 | *Ptr-HD-ZIP27* | Ptr-PO85_SNP49 | *Ptr-PO85* | V | AA | 0.49 | 4.06E-05 |
| Ptr-HD-ZIP27_SNP63 | *Ptr-HD-ZIP27* | Ptr-PO85_SNP49 | *Ptr-PO85* | V | AA | 0.49 | 4.06E-05 |
| Ptr-HCT1_SNP20 | *Ptr-HCT1* | Ptr-COMT22_SNP58 | *Ptr-COMT22* | CC | AA | 14.70 | 4.07E-05 |
| Ptr-PAL3_SNP83 | *Ptr-PAL3* | Ptr-HD-ZIP24_SNP81 | *Ptr-HD-ZIP24* | HC | AA | -19.50 | 4.09E-05 |
| Pto-MiR167a_SNP20 | *Pto-MIR167a* | Ptr-HCT2_SNP9 | *Ptr-HCT2* | V | DD | -1.02 | 4.16E-05 |
| Ptr-PO70_SNP64 | *Ptr-PO70* | Ptr-PO85_SNP15 | *Ptr-PO85* | V | DA | -0.58 | 4.18E-05 |
| Ptr-HCT12_SNP88 | *Ptr-HCT12* | Pto-MiR396g_SNP22 | *Pto-MIR396g* | DBH | AA | 6.74 | 4.19E-05 |
| Ptr-HCT8_SNP80 | *Ptr-HCT8* | Ptr-4CL4_SNP69 | *Ptr-4CL4* | V | DA | 1.12 | 4.21E-05 |
| Ptr-C2H2-72_SNP33 | *Ptr-C2H2-72* | Ptr-4CL4_SNP69 | *Ptr-4CL4* | V | AA | 0.84 | 4.23E-05 |
| Ptr-PO17_SNP20 | *Ptr-PO17* | Ptr-bHLH15_SNP56 | *Ptr-bHLH15* | DBH | AA | -3.96 | 4.23E-05 |
| Ptr-COMT25_SNP118 | *Ptr-COMT25* | Ptr-COMT22_SNP58 | *Ptr-COMT22* | HC | AA | -16.20 | 4.24E-05 |
| Ptr-PO23_SNP8 | *Ptr-PO23* | Ptr-PO27_SNP31 | *Ptr-PO27* | HEC | AA | -6.15 | 4.24E-05 |
| Ptr-HD-ZIP4_SNP11 | *Ptr-HD-ZIP4* | Ptr-HD-ZIP28_SNP21 | *Ptr-HD-ZIP28* | V | AA | -0.42 | 4.32E-05 |
| Ptr-HCT1_SNP24 | *Ptr-HCT1* | L40_SNP30 | *LncRNA locus 40* | HC | AD | -31.90 | 4.36E-05 |
| Ptr-PO23_SNP7 | *Ptr-PO23* | Ptr-bHLH15_SNP56 | *Ptr-bHLH15* | HC | AA | -11.30 | 4.37E-05 |
| Ptr-PO40_SNP145 | *Ptr-PO40* | Ptr-COMT12_SNP122 | *Ptr-COMT12* | V | DD | -0.99 | 4.38E-05 |
| Ptr-PAL3_SNP102 | *Ptr-PAL3* | Ptr-HD-ZIP21_SNP40 | *Ptr-HD-ZIP21* | DBH | AA | 4.95 | 4.40E-05 |
| Pto-MiR167a_SNP11 | *Pto-MIR167a* | Ptr-HD-ZIP51_SNP85 | *Ptr-HD-ZIP51* | V | AA | 0.80 | 4.41E-05 |
| Ptr-COMT25_SNP126 | *Ptr-COMT25* | L19_SNP31 | *LncRNA locus 19* | V | AD | 0.76 | 4.42E-05 |
| L19_SNP77 | *LncRNA locus 19* | Ptr-CCR26_SNP4 | *Ptr-CCR26* | V | DA | 0.86 | 4.50E-05 |
| Ptr-PO17_SNP31 | *Ptr-PO17* | L19_SNP31 | *LncRNA locus 19* | V | AD | 0.97 | 4.52E-05 |
| L10_SNP110 | *LncRNA locus 10* | Ptr-CAD16_SNP98 | *Ptr-CAD16* | V | AA | -0.75 | 4.55E-05 |
| Ptr-COMT25_SNP102 | *Ptr-COMT25* | Ptr-PO72_SNP18 | *Ptr-PO72* | HC | DA | 13.30 | 4.56E-05 |
| L33_SNP29 | *LncRNA locus 33* | Ptr-CAD6_SNP11 | *Ptr-CAD6* | HEC | AA | 11.40 | 4.57E-05 |
| Ptr-4CL20_SNP112 | *Ptr-4CL20* | Ptr-4CL4_SNP70 | *Ptr-4CL4* | V | AD | -0.98 | 4.59E-05 |
| Ptr-4CL20_SNP112 | *Ptr-4CL20* | Ptr-4CL4_SNP71 | *Ptr-4CL4* | V | AD | -0.98 | 4.59E-05 |
| Ptr-bHLH18_SNP111 | *Ptr-bHLH18* | Ptr-HD-ZIP55_SNP30 | *Ptr-HD-ZIP55* | V | DD | -1.04 | 4.62E-05 |
| Ptr-PO39_SNP87 | *Ptr-PO39* | Ptr-PO41_SNP52 | *Ptr-PO41* | DBH | DA | 12.20 | 4.64E-05 |
| Ptr-COMT25_SNP4 | *Ptr-COMT25* | L19_SNP18 | *LncRNA locus 19* | HC | AD | 27.60 | 4.65E-05 |
| L32_SNP19 | *LncRNA locus 32* | Ptr-PO40_SNP142 | *Ptr-PO40* | HC | AA | -10.30 | 4.67E-05 |
| Ptr-PO72_SNP10 | *Ptr-PO72* | Ptr-PO85_SNP54 | *Ptr-PO85* | V | AD | 0.38 | 4.68E-05 |
| Ptr-bZIP1_SNP38 | *Ptr-bZIP1* | Ptr-PO70_SNP19 | *Ptr-PO70* | HC | DD | -35.20 | 4.71E-05 |
| Ptr-CCR26_SNP165 | *Ptr-CCR26* | Ptr-PO85_SNP1 | *Ptr-PO85* | HC | AA | -7.16 | 4.72E-05 |
| Ptr-CCR13_SNP7 | *Ptr-CCR13* | Ptr-HD-ZIP49_SNP84 | *Ptr-HD-ZIP49* | FW | DD | -4.69 | 4.74E-05 |
| Ptr-PO82_SNP87 | *Ptr-PO82* | Ptr-COMT12_SNP120 | *Ptr-COMT12* | HC | AA | 16.20 | 4.79E-05 |
| L33_SNP4 | *LncRNA locus 33* | Ptr-COMT22_SNP58 | *Ptr-COMT22* | HC | AD | 21.00 | 4.80E-05 |
| Ptr-AT-Hook1_SNP44 | *Ptr-AT-Hook1* | Ptr-PO34_SNP14 | *Ptr-PO34* | FW | DD | 4.57 | 4.81E-05 |
| Ptr-PAL3_SNP100 | *Ptr-PAL3* | L49_SNP33 | *LncRNA locus 49* | V | DD | -1.79 | 4.85E-05 |
| Ptr-PAL3_SNP100 | *Ptr-PAL3* | L49_SNP34 | *LncRNA locus 49* | V | DD | -1.79 | 4.85E-05 |
| L19_SNP138 | *LncRNA locus 19* | Ptr-C4H2_SNP4 | *Ptr-C4H2* | LC | AA | 2.33 | 4.85E-05 |
| L19_SNP37 | *LncRNA locus 19* | Ptr-PO70_SNP19 | *Ptr-PO70* | HC | AD | 21.20 | 4.86E-05 |
| Ptr-PAL3_SNP100 | *Ptr-PAL3* | Ptr-PO82_SNP87 | *Ptr-PO82* | DBH | DD | -25.60 | 4.86E-05 |
| Ptr-CAD10_SNP214 | *Ptr-CAD10* | L49_SNP33 | *LncRNA locus 49* | DBH | AD | -16.50 | 4.87E-05 |
| Ptr-CAD10_SNP214 | *Ptr-CAD10* | L49_SNP34 | *LncRNA locus 49* | DBH | AD | -16.50 | 4.87E-05 |
| Ptr-PO70_SNP63 | *Ptr-PO70* | Ptr-PO85_SNP15 | *Ptr-PO85* | DBH | DA | -19.10 | 4.88E-05 |
| Ptr-PO35_SNP1 | *Ptr-PO35* | Ptr-C2H2-72_SNP61 | *Ptr-C2H2-72* | V | DD | 1.92 | 4.89E-05 |
| Ptr-C3H3_SNP17 | *Ptr-C3H3* | Ptr-C4H3_SNP108 | *Ptr-C4H3* | HC | DA | 7.05 | 4.89E-05 |
| L09_SNP30 | *LncRNA locus 9* | Ptr-HD-ZIP13_SNP18 | *Ptr-HD-ZIP13* | V | DD | -1.85 | 4.91E-05 |
| Ptr-CAD6_SNP93 | Ptr-CAD6 | Ptr-CSE2_SNP19 | Ptr-CSE2 | HEC | AA | 9.65 | 4.91E-05 |
| Ptr-HCT1_SNP28 | *Ptr-HCT1* | Ptr-bZIP5_SNP84 | *Ptr-bZIP5* | V | AA | 0.71 | 4.92E-05 |
| L09_SNP30 | *LncRNA locus 9* | Ptr-PO41_SNP35 | *Ptr-PO41* | V | AA | -0.92 | 4.92E-05 |
| Ptr-PO27_SNP31 | *Ptr-PO27* | Pto-MiR167d_SNP42 | *Pto-MIR167d* | V | DD | 1.67 | 4.93E-05 |
| L19_SNP138 | *LncRNA locus 19* | Ptr-CCR26_SNP4 | *Ptr-CCR26* | V | DA | -0.45 | 4.94E-05 |
| Ptr-4CL20_SNP107 | *Ptr-4CL20* | Ptr-bHLH15_SNP56 | *Ptr-bHLH15* | V | AA | 0.33 | 4.95E-05 |
| Ptr-HCT1_SNP22 | *Ptr-HCT1* | Ptr-C4H2_SNP4 | *Ptr-C4H2* | LC | DD | -6.07 | 4.95E-05 |
| Pto-MiR167d_SNP42 | *Pto-MIR167d* | Ptr-C2H2-72_SNP61 | *Ptr-C2H2-72* | V | DD | 1.86 | 4.99E-05 |
| L19_SNP19 | *LncRNA locus 19* | Ptr-AT-Hook4_SNP56 | *Ptr-AT-Hook4* | HC | AD | 19.40 | 5.01E-05 |
| L09_SNP62 | *LncRNA locus 9* | L19_SNP19 | *LncRNA locus 19* | CC | DA | 17.80 | 5.05E-05 |
| Ptr-COMT16_SNP8 | *Ptr-COMT16* | Ptr-4CL12_SNP29 | *Ptr-4CL12* | V | AD | 0.99 | 5.07E-05 |
| Ptr-HCT12_SNP9 | *Ptr-HCT12* | Ptr-bZIP5_SNP55 | *Ptr-bZIP5* | V | DD | -1.05 | 5.07E-05 |
| Ptr-PO70_SNP19 | *Ptr-PO70* | Ptr-4CL12_SNP8 | *Ptr-4CL12* | HC | DA | 23.60 | 5.08E-05 |
| Ptr-COMT9_SNP15 | *Ptr-COMT9* | Ptr-Myb/SANT2_SNP21 | *Ptr-Myb/SANT2* | HEC | DD | -28.90 | 5.08E-05 |
| Ptr-PAL3_SNP117 | *Ptr-PAL3* | L62_SNP113 | *LncRNA locus 62* | CC | DD | 21.80 | 5.10E-05 |
| Ptr-4CL12_SNP29 | *Ptr-4CL12* | Ptr-CCoAOMT5_SNP20 | *Ptr-CCoAOMT5* | HC | AA | 10.40 | 5.10E-05 |
| Ptr-CAD10_SNP77 | *Ptr-CAD10* | Ptr-HD-ZIP13_SNP16 | *Ptr-HD-ZIP13* | HEC | DD | -24.10 | 5.10E-05 |
| L40_SNP2 | *LncRNA locus 40* | Ptr-PO72_SNP18 | *Ptr-PO72* | V | DD | 1.70 | 5.13E-05 |
| Ptr-COMT25_SNP4 | *Ptr-COMT25* | L19_SNP16 | *LncRNA locus 19* | HC | AD | 27.40 | 5.14E-05 |
| L37_SNP4 | *LncRNA locus 37* | Ptr-COMT22_SNP58 | *Ptr-COMT22* | HC | AA | -19.60 | 5.14E-05 |
| Ptr-PO70_SNP64 | *Ptr-PO70* | Ptr-HD-ZIP55_SNP30 | *Ptr-HD-ZIP55* | V | DD | -1.28 | 5.14E-05 |
| Ptr-AT-Hook1_SNP21 | *Ptr-AT-Hook1* | Ptr-bHLH15_SNP57 | *Ptr-bHLH15* | HC | AD | -20.80 | 5.15E-05 |
| Ptr-4CL20_SNP107 | *Ptr-4CL20* | Ptr-Myb/SANT2_SNP21 | *Ptr-Myb/SANT2* | HEC | DD | -25.30 | 5.15E-05 |
| Ptr-Myb/SANT2_SNP21 | *Ptr-Myb/SANT2* | Ptr-HD-ZIP48_SNP15 | *Ptr-HD-ZIP48* | HEC | DA | 20.20 | 5.18E-05 |
| Ptr-bHLH2_SNP16 | *Ptr-bHLH2* | L49_SNP33 | *LncRNA locus 49* | DBH | AD | 17.40 | 5.20E-05 |
| Ptr-bHLH2_SNP16 | *Ptr-bHLH2* | L49_SNP34 | *LncRNA locus 49* | DBH | AD | 17.40 | 5.20E-05 |
| Ptr-CCR10_SNP11 | *Ptr-CCR10* | L10_SNP109 | *LncRNA locus 10* | HC | DA | 33.80 | 5.23E-05 |
| Ptr-COMT25_SNP109 | *Ptr-COMT25* | Ptr-PO72_SNP40 | *Ptr-PO72* | HC | DA | -26.20 | 5.23E-05 |
| Ptr-COMT25_SNP109 | *Ptr-COMT25* | Ptr-HD-ZIP49_SNP69 | *Ptr-HD-ZIP49* | V | AA | 0.35 | 5.24E-05 |
| Ptr-4CL3_SNP28 | *Ptr-4CL3* | Ptr-COMT12_SNP122 | *Ptr-COMT12* | V | AD | 1.10 | 5.25E-05 |
| L17_SNP4 | *LncRNA locus 17* | Ptr-4CL9_SNP73 | *Ptr-4CL9* | HC | AA | 5.09 | 5.27E-05 |
| Ptr-C3H2_SNP39 | *Ptr-C3H2* | Ptr-bHLH10_SNP39 | *Ptr-bHLH10* | DBH | DA | 5.76 | 5.27E-05 |
| Ptr-HCT8_SNP80 | *Ptr-HCT8* | Ptr-PO70_SNP63 | *Ptr-PO70* | HC | DA | 27.50 | 5.30E-05 |
| Ptr-HD-ZIP10_SNP76 | *Ptr-HD-ZIP10* | Ptr-bHLH11_SNP43 | *Ptr-bHLH11* | MFA | AA | 2.66 | 5.31E-05 |
| Ptr-bHLH15_SNP57 | *Ptr-bHLH15* | L70_SNP46 | *LncRNA locus 70* | HC | DA | -21.30 | 5.32E-05 |
| Ptr-COMT25_SNP110 | *Ptr-COMT25* | Ptr-HD-ZIP49_SNP69 | *Ptr-HD-ZIP49* | V | AA | 0.34 | 5.32E-05 |
| Ptr-HCT1_SNP20 | *Ptr-HCT1* | Ptr-PO70_SNP64 | *Ptr-PO70* | HC | AA | 17.40 | 5.34E-05 |
| Ptr-HD-ZIP13_SNP9 | *Ptr-HD-ZIP13* | L37_SNP18 | *LncRNA locus 37* | V | DA | -0.83 | 5.36E-05 |
| Ptr-CCR28_SNP94 | *Ptr-CCR28* | Ptr-bZIP5_SNP84 | *Ptr-bZIP5* | FW | DA | -8.49 | 5.43E-05 |
| L19_SNP67 | *LncRNA locus 19* | Ptr-PO70_SNP64 | *Ptr-PO70* | HC | AA | -20.40 | 5.43E-05 |
| L19_SNP68 | *LncRNA locus 19* | Ptr-PO70_SNP64 | *Ptr-PO70* | HC | AA | -20.40 | 5.43E-05 |
| Ptr-COMT25_SNP120 | *Ptr-COMT25* | L19_SNP138 | *LncRNA locus 19* | HC | AA | -20.20 | 5.44E-05 |
| L19_SNP67 | *LncRNA locus 19* | Ptr-Myb/SANT2_SNP21 | *Ptr-Myb/SANT2* | HEC | DD | -26.90 | 5.46E-05 |
| L19_SNP68 | *LncRNA locus 19* | Ptr-Myb/SANT2_SNP21 | *Ptr-Myb/SANT2* | HEC | DD | -26.90 | 5.46E-05 |
| Ptr-HCT1_SNP3 | *Ptr-HCT1* | Ptr-CAD16_SNP98 | *Ptr-CAD16* | V | AA | -0.77 | 5.50E-05 |
| Ptr-4CL9_SNP73 | *Ptr-4CL9* | Ptr-bHLH11_SNP44 | *Ptr-bHLH11* | HC | AA | -6.61 | 5.53E-05 |
| Ptr-CCR13_SNP31 | *Ptr-CCR13* | Ptr-COMT25_SNP4 | *Ptr-COMT25* | HC | AA | 20.00 | 5.53E-05 |
| L19_SNP18 | *LncRNA locus 19* | L58_SNP7 | *LncRNA locus 58* | HC | DD | -45.40 | 5.56E-05 |
| Ptr-CAD10_SNP214 | *Ptr-CAD10* | L40_SNP2 | *LncRNA locus 40* | V | AD | -1.30 | 5.58E-05 |
| Ptr-C3H2_SNP39 | *Ptr-C3H2* | Ptr-bHLH13_SNP39 | *Ptr-bHLH13* | HC | AA | 15.90 | 5.61E-05 |
| L19_SNP37 | *LncRNA locus 19* | Ptr-HD-ZIP24_SNP81 | *Ptr-HD-ZIP24* | V | AA | 0.22 | 5.61E-05 |
| L32_SNP19 | *LncRNA locus 32* | Ptr-HCT12_SNP9 | *Ptr-HCT12* | V | AD | -0.70 | 5.62E-05 |
| L19_SNP16 | *LncRNA locus 19* | L58_SNP7 | *LncRNA locus 58* | HC | DD | -45.30 | 5.63E-05 |
| Ptr-CAD14_SNP81 | *Ptr-CAD14* | Ptr-HCT12_SNP9 | *Ptr-HCT12* | V | DA | -1.15 | 5.65E-05 |
| Ptr-CAD10_SNP77 | *Ptr-CAD10* | Ptr-4CL4_SNP22 | *Ptr-4CL4* | V | AD | 0.84 | 5.67E-05 |
| Ptr-C3H3_SNP15 | *Ptr-C3H3* | Ptr-CCR28_SNP94 | *Ptr-CCR28* | FW | AD | -8.31 | 5.67E-05 |
| Ptr-4CL20_SNP112 | *Ptr-4CL20* | Ptr-4CL4_SNP72 | *Ptr-4CL4* | V | AD | -0.96 | 5.69E-05 |
| Ptr-PO40_SNP142 | *Ptr-PO40* | Ptr-LAC43_SNP28 | *Ptr-LAC43* | V | DA | 0.88 | 5.69E-05 |
| Ptr-COMT25_SNP110 | *Ptr-COMT25* | Ptr-C4H3_SNP108 | *Ptr-C4H3* | HC | DD | 25.30 | 5.70E-05 |
| Ptr-CCR26_SNP4 | *Ptr-CCR26* | Ptr-4CL2_SNP29 | *Ptr-4CL2* | V | AA | 0.36 | 5.71E-05 |
| Ptr-AT-Hook2_SNP90 | *Ptr-AT-Hook2* | L40_SNP2 | *LncRNA locus 40* | V | DD | 1.49 | 5.72E-05 |
| Ptr-LAC31_SNP6 | *Ptr-LAC31* | Ptr-C2H2-72_SNP33 | *Ptr-C2H2-72* | V | AA | -0.44 | 5.74E-05 |
| Ptr-HCT1_SNP10 | *Ptr-HCT1* | L19_SNP77 | *LncRNA locus 19* | DBH | DD | -17.50 | 5.77E-05 |
| L13_SNP130 | *LncRNA locus 13* | Ptr-COMT4_SNP27 | *Ptr-COMT4* | V | AA | -0.87 | 5.80E-05 |
| Ptr-PO28_SNP80 | *Ptr-PO28* | L25_SNP3 | *LncRNA locus 25* | FW | DA | -4.33 | 5.83E-05 |
| Pto-MiR167a_SNP8 | *Pto-MIR167a* | Ptr-bZIP5_SNP69 | *Ptr-bZIP5* | V | AD | 0.89 | 5.84E-05 |
| Ptr-CAD10_SNP69 | *Ptr-CAD10* | Ptr-AT-Hook1_SNP11 | *Ptr-AT-Hook1* | HC | DD | 30.00 | 5.87E-05 |
| Ptr-COMT25_SNP110 | *Ptr-COMT25* | Ptr-PO72_SNP40 | *Ptr-PO72* | HC | DA | -26.00 | 5.89E-05 |
| Ptr-CCR13_SNP7 | *Ptr-CCR13* | Ptr-HD-ZIP18_SNP13 | *Ptr-HD-ZIP18* | V | DA | -0.68 | 5.93E-05 |
| Ptr-COMT25_SNP118 | *Ptr-COMT25* | L25_SNP2 | *LncRNA locus 25* | HC | AD | 21.10 | 5.96E-05 |
| Ptr-HCT1_SNP28 | *Ptr-HCT1* | Ptr-bZIP5_SNP85 | *Ptr-bZIP5* | V | AA | -0.67 | 5.99E-05 |
| Ptr-CCR13_SNP7 | *Ptr-CCR13* | Ptr-HD-ZIP48_SNP15 | *Ptr-HD-ZIP48* | FW | DD | -4.28 | 5.99E-05 |
| L02_SNP15 | *LncRNA locus 2* | Ptr-PAL3_SNP91 | *Ptr-PAL3* | HC | AD | -31.40 | 6.01E-05 |
| Ptr-PO43_SNP9 | *Ptr-PO43* | L40_SNP2 | *LncRNA locus 40* | V | DD | 1.46 | 6.02E-05 |
| Ptr-PO17_SNP32 | *Ptr-PO17* | Ptr-COMT22_SNP58 | *Ptr-COMT22* | HC | AA | -14.00 | 6.03E-05 |
| L30_SNP18 | *LncRNA locus 30* | Ptr-bHLH10_SNP39 | *Ptr-bHLH10* | DBH | AD | -9.38 | 6.04E-05 |
| Ptr-COMT25_SNP110 | *Ptr-COMT25* | Ptr-PO39_SNP87 | *Ptr-PO39* | DBH | AD | -10.40 | 6.05E-05 |
| L40_SNP2 | *LncRNA locus 40* | Ptr-C2H2-77_SNP57 | *Ptr-C2H2-77* | HC | DD | -33.00 | 6.06E-05 |
| Ptr-LAC2_SNP20 | *Ptr-LAC2* | Ptr-PO72_SNP18 | *Ptr-PO72* | V | AD | 0.84 | 6.07E-05 |
| Ptr-HD-ZIP24_SNP81 | *Ptr-HD-ZIP24* | Ptr-4CL21_SNP31 | *Ptr-4CL21* | HC | DD | 25.10 | 6.09E-05 |
| Ptr-CAD10_SNP69 | *Ptr-CAD10* | Ptr-bHLH10_SNP39 | *Ptr-bHLH10* | DBH | DA | 5.19 | 6.22E-05 |
| Ptr-HD-ZIP13_SNP24 | *Ptr-HD-ZIP13* | Ptr-HCT12_SNP9 | *Ptr-HCT12* | HC | AA | -11.60 | 6.22E-05 |
| Ptr-PO70_SNP64 | *Ptr-PO70* | Ptr-PO85_SNP50 | *Ptr-PO85* | FW | DA | 4.76 | 6.22E-05 |
| Ptr-CAD10_SNP187 | *Ptr-CAD10* | Ptr-HCT2_SNP9 | *Ptr-HCT2* | V | DD | -1.01 | 6.23E-05 |
| Ptr-CAD10_SNP170 | *Ptr-CAD10* | Ptr-C4H3_SNP108 | *Ptr-C4H3* | HC | AA | 16.00 | 6.26E-05 |
| Ptr-4CL3_SNP44 | *Ptr-4CL3* | Ptr-CAD13_SNP97 | *Ptr-CAD13* | HC | DD | 39.00 | 6.26E-05 |
| Ptr-4CL3_SNP44 | *Ptr-4CL3* | Ptr-CAD13_SNP98 | *Ptr-CAD13* | HC | DD | 39.00 | 6.26E-05 |
| Pto-MiR167a_SNP20 | *Pto-MIR167a* | Ptr-PO41_SNP35 | *Ptr-PO41* | V | AA | 0.47 | 6.31E-05 |
| Ptr-COMT25_SNP150 | *Ptr-COMT25* | Ptr-C4H2_SNP4 | *Ptr-C4H2* | LC | AD | 3.00 | 6.33E-05 |
| Ptr-PAL3_SNP92 | *Ptr-PAL3* | L30_SNP18 | *LncRNA locus 30* | HC | AA | -13.60 | 6.38E-05 |
| Ptr-PO54_SNP54 | *Ptr-PO54* | Ptr-4CL12_SNP29 | *Ptr-4CL12* | LC | DD | -7.02 | 6.43E-05 |
| L26_SNP42 | *LncRNA locus 26* | Ptr-PO43_SNP28 | *Ptr-PO43* | DBH | AA | 3.75 | 6.44E-05 |
| L19_SNP77 | *LncRNA locus 19* | Ptr-PO27_SNP30 | *Ptr-PO27* | V | DD | 1.64 | 6.46E-05 |
| L33_SNP4 | *LncRNA locus 33* | Ptr-C2H2-72_SNP33 | *Ptr-C2H2-72* | V | AA | 0.55 | 6.49E-05 |
| L17_SNP4 | *LncRNA locus 17* | Ptr-AT-Hook1_SNP28 | *Ptr-AT-Hook1* | HC | AA | 11.60 | 6.51E-05 |
| Ptr-COMT25_SNP109 | *Ptr-COMT25* | Ptr-PO39_SNP87 | *Ptr-PO39* | DBH | AD | -10.50 | 6.52E-05 |
| Ptr-PO27_SNP30 | *Ptr-PO27* | Ptr-PO85_SNP54 | *Ptr-PO85* | LC | AD | 2.37 | 6.52E-05 |
| Ptr-COMT25_SNP126 | *Ptr-COMT25* | Ptr-HD-ZIP13_SNP22 | *Ptr-HD-ZIP13* | LC | DD | -8.83 | 6.56E-05 |
| L33_SNP12 | *LncRNA locus 33* | Ptr-C3H2_SNP39 | *Ptr-C3H2* | FL | DA | 0.11 | 6.58E-05 |
| Ptr-bZIP1_SNP38 | *Ptr-bZIP1* | Ptr-PO40_SNP142 | *Ptr-PO40* | V | AD | -0.90 | 6.58E-05 |
| L20_SNP51 | *LncRNA locus 20* | Ptr-HCT16_SNP51 | *Ptr-HCT16* | H | DA | -2.98 | 6.59E-05 |
| Ptr-HD-ZIP13_SNP18 | *Ptr-HD-ZIP13* | L50_SNP110 | *LncRNA locus 50* | V | DD | -1.26 | 6.63E-05 |
| Ptr-PO31_SNP8 | *Ptr-PO31* | L40_SNP2 | *LncRNA locus 40* | V | DD | 1.81 | 6.68E-05 |
| Ptr-PO40_SNP142 | *Ptr-PO40* | Ptr-PO82_SNP87 | *Ptr-PO82* | V | DA | 0.96 | 6.68E-05 |
| Pto-MiR167d_SNP30 | *Pto-MIR167d* | Ptr-4CL12_SNP29 | *Ptr-4CL12* | DBH | DD | 17.20 | 6.69E-05 |
| Ptr-HCT7_SNP9 | *Ptr-HCT7* | Ptr-HD-ZIP35_SNP44 | *Ptr-HD-ZIP35* | V | DA | 0.49 | 6.69E-05 |
| Pto-MiR167a_SNP41 | *Pto-MIR167a* | Ptr-PO41_SNP19 | *Ptr-PO41* | FL | DD | -0.17 | 6.70E-05 |
| Pto-MiR167a_SNP20 | *Pto-MIR167a* | Ptr-PO41_SNP35 | *Ptr-PO41* | DBH | DD | -13.00 | 6.70E-05 |
| Ptr-HD-ZIP13_SNP2 | *Ptr-HD-ZIP13* | Ptr-COMT22_SNP58 | *Ptr-COMT22* | HC | AA | 16.20 | 6.71E-05 |
| L49_SNP33 | *LncRNA locus 49* | L58_SNP20 | *LncRNA locus 58* | DBH | DA | -17.30 | 6.72E-05 |
| L49_SNP34 | *LncRNA locus 49* | L58_SNP20 | *LncRNA locus 58* | DBH | DA | -17.30 | 6.72E-05 |
| Ptr-HD-ZIP13_SNP16 | *Ptr-HD-ZIP13* | Ptr-bHLH21_SNP26 | *Ptr-bHLH21* | HC | AD | -17.40 | 6.74E-05 |
| Ptr-PO40_SNP145 | *Ptr-PO40* | Ptr-HCT12_SNP9 | *Ptr-HCT12* | V | DA | 0.40 | 6.74E-05 |
| Ptr-COMT25_SNP109 | *Ptr-COMT25* | L42_SNP3 | *LncRNA locus 42* | HC | AA | -8.49 | 6.76E-05 |
| Ptr-CAD6_SNP11 | *Ptr-CAD6* | Ptr-HD-ZIP51_SNP85 | *Ptr-HD-ZIP51* | HC | AA | -15.10 | 6.84E-05 |
| L13_SNP78 | *LncRNA locus 13* | Ptr-HD-ZIP55_SNP2 | *Ptr-HD-ZIP55* | HC | AA | -15.30 | 6.84E-05 |
| Ptr-HCT12_SNP87 | *Ptr-HCT12* | Ptr-LAC43_SNP28 | *Ptr-LAC43* | HEC | AA | 11.70 | 6.85E-05 |
| L26_SNP42 | *LncRNA locus 26* | Ptr-bHLH18_SNP21 | *Ptr-bHLH18* | V | AD | 0.24 | 6.86E-05 |
| Ptr-COMT25_SNP104 | *Ptr-COMT25* | L68_SNP1 | *LncRNA locus 68* | HC | DA | 19.40 | 6.87E-05 |
| L09_SNP26 | *LncRNA locus 9* | Ptr-PO41_SNP35 | *Ptr-PO41* | V | AA | 0.92 | 6.87E-05 |
| Ptr-bHLH21_SNP29 | *Ptr-bHLH21* | Ptr-HD-ZIP28_SNP56 | *Ptr-HD-ZIP28* | HC | DA | -30.00 | 6.94E-05 |
| Ptr-bHLH21_SNP29 | *Ptr-bHLH21* | Ptr-HD-ZIP28_SNP57 | *Ptr-HD-ZIP28* | HC | DA | -30.00 | 6.94E-05 |
| Ptr-C3H3_SNP15 | *Ptr-C3H3* | L32_SNP19 | *LncRNA locus 32* | HC | DA | 12.40 | 6.95E-05 |
| L32_SNP111 | *LncRNA locus 32* | Ptr-HCT12_SNP9 | *Ptr-HCT12* | HC | AA | 13.10 | 6.95E-05 |
| Ptr-4CL20_SNP116 | *Ptr-4CL20* | Ptr-HD-ZIP51_SNP14 | *Ptr-HD-ZIP51* | DBH | DA | -10.30 | 6.96E-05 |
| Ptr-PO41_SNP58 | *Ptr-PO41* | L37_SNP4 | *LncRNA locus 37* | HC | AD | 28.50 | 6.97E-05 |
| Ptr-COMT25_SNP119 | *Ptr-COMT25* | Ptr-PO70_SNP19 | *Ptr-PO70* | HC | AD | -31.80 | 6.97E-05 |
| Ptr-C2H2-77_SNP70 | *Ptr-C2H2-77* | Ptr-4CL4_SNP26 | *Ptr-4CL4* | V | AD | 0.23 | 6.99E-05 |
| Ptr-Myb/SANT2_SNP21 | *Ptr-Myb/SANT2* | Ptr-HD-ZIP56_SNP99 | *Ptr-HD-ZIP56* | HEC | DA | 17.20 | 6.99E-05 |
| L40_SNP30 | *LncRNA locus 40* | L50_SNP110 | *LncRNA locus 50* | HC | DD | -37.90 | 7.04E-05 |
| Ptr-CCR13_SNP7 | *Ptr-CCR13* | Ptr-PO34_SNP14 | *Ptr-PO34* | FW | AD | -2.39 | 7.04E-05 |
| Ptr-COMT25_SNP104 | *Ptr-COMT25* | Ptr-HD-ZIP49_SNP64 | *Ptr-HD-ZIP49* | HC | DA | -7.85 | 7.06E-05 |
| Ptr-COMT22_SNP58 | *Ptr-COMT22* | Ptr-4CL2_SNP29 | *Ptr-4CL2* | HC | AD | -21.90 | 7.08E-05 |
| Ptr-CAD10_SNP77 | *Ptr-CAD10* | Ptr-CAD14_SNP81 | *Ptr-CAD14* | V | AD | 1.13 | 7.08E-05 |
| Ptr-LAC4_SNP49 | *Ptr-LAC4* | L58_SNP7 | *LncRNA locus 58* | HC | DD | -47.10 | 7.09E-05 |
| Ptr-LAC5_SNP16 | *Ptr-LAC5* | L58_SNP8 | *LncRNA locus 58* | HEC | AD | 25.60 | 7.09E-05 |
| Ptr-COMT25_SNP104 | *Ptr-COMT25* | Ptr-HD-ZIP24_SNP81 | *Ptr-HD-ZIP24* | V | AA | -0.41 | 7.09E-05 |
| Ptr-4CL3_SNP28 | *Ptr-4CL3* | Ptr-4CL4_SNP76 | *Ptr-4CL4* | HC | DD | -36.20 | 7.10E-05 |
| Pto-MiR167a_SNP41 | *Pto-MIR167a* | L37_SNP18 | *LncRNA locus 37* | V | AA | -0.52 | 7.12E-05 |
| Ptr-CCR9_SNP20 | *Ptr-CCR9* | Ptr-4CL4_SNP70 | *Ptr-4CL4* | V | DA | 0.56 | 7.12E-05 |
| Ptr-CCR9_SNP20 | *Ptr-CCR9* | Ptr-4CL4_SNP71 | *Ptr-4CL4* | V | DA | 0.56 | 7.12E-05 |
| Ptr-HD-ZIP13_SNP9 | *Ptr-HD-ZIP13* | Ptr-bHLH17_SNP5 | *Ptr-bHLH17* | V | AA | 0.82 | 7.12E-05 |
| Ptr-bHLH11_SNP44 | *Ptr-bHLH11* | Ptr-PO52_SNP37 | *Ptr-PO52* | CC | AD | 6.31 | 7.13E-05 |
| Ptr-COMT25_SNP102 | *Ptr-COMT25* | Ptr-HD-ZIP49_SNP69 | *Ptr-HD-ZIP49* | HC | AA | 9.91 | 7.15E-05 |
| L41_SNP159 | *LncRNA locus 41* | L62_SNP113 | *LncRNA locus 62* | MFA | AA | 2.87 | 7.17E-05 |
| Ptr-CCR28_SNP94 | *Ptr-CCR28* | Ptr-COMT12_SNP40 | *Ptr-COMT12* | FW | DA | 8.36 | 7.17E-05 |
| Ptr-HCT1_SNP24 | *Ptr-HCT1* | Ptr-PO70_SNP64 | *Ptr-PO70* | HC | AA | 19.10 | 7.17E-05 |
| Ptr-COMT25_SNP111 | *Ptr-COMT25* | L19_SNP77 | *LncRNA locus 19* | V | DD | 1.63 | 7.21E-05 |
| L19_SNP77 | *LncRNA locus 19* | Ptr-C2H2-72_SNP61 | *Ptr-C2H2-72* | V | DD | 1.77 | 7.23E-05 |
| L26_SNP42 | *LncRNA locus 26* | Ptr-CCR9_SNP19 | *Ptr-CCR9* | V | AA | -0.39 | 7.23E-05 |
| Ptr-Myb/SANT2_SNP21 | *Ptr-Myb/SANT2* | Ptr-PO65_SNP66 | *Ptr-PO65* | HEC | DA | -17.60 | 7.23E-05 |
| L33_SNP4 | *LncRNA locus 33* | Ptr-bHLH15_SNP57 | *Ptr-bHLH15* | HEC | AD | 19.20 | 7.24E-05 |
| Ptr-HCT12_SNP9 | *Ptr-HCT12* | Ptr-PO75_SNP15 | *Ptr-PO75* | HC | AA | 12.10 | 7.29E-05 |
| Ptr-HCT16_SNP51 | *Ptr-HCT16* | Ptr-bHLH18_SNP45 | *Ptr-bHLH18* | V | AA | -0.38 | 7.31E-05 |
| Ptr-HD-ZIP4_SNP14 | *Ptr-HD-ZIP4* | Ptr-PO40_SNP142 | *Ptr-PO40* | HC | DD | -29.80 | 7.31E-05 |
| Ptr-CCR26_SNP4 | *Ptr-CCR26* | Ptr-bHLH23_SNP29 | *Ptr-bHLH23* | V | AD | -0.59 | 7.32E-05 |
| Ptr-HD-ZIP4_SNP11 | *Ptr-HD-ZIP4* | Ptr-CAD10_SNP77 | *Ptr-CAD10* | V | DA | -0.68 | 7.34E-05 |
| Ptr-HD-ZIP13_SNP16 | *Ptr-HD-ZIP13* | Ptr-C2H2-77_SNP57 | *Ptr-C2H2-77* | HC | AA | 8.20 | 7.36E-05 |
| L17_SNP4 | *LncRNA locus 17* | L32_SNP19 | *LncRNA locus 32* | HC | AD | -10.40 | 7.38E-05 |
| L13_SNP23 | *LncRNA locus 13* | L25_SNP3 | *LncRNA locus 25* | V | AD | 0.87 | 7.43E-05 |
| Ptr-COMT25_SNP107 | *Ptr-COMT25* | Ptr-HD-ZIP49_SNP69 | *Ptr-HD-ZIP49* | V | AA | 0.31 | 7.43E-05 |
| Ptr-HCT6_SNP25 | *Ptr-HCT6* | Ptr-PO70_SNP19 | *Ptr-PO70* | HC | AD | -20.60 | 7.44E-05 |
| Ptr-CAD10_SNP214 | *Ptr-CAD10* | L19_SNP31 | *LncRNA locus 19* | HC | DA | -28.40 | 7.47E-05 |
| L02_SNP13 | *LncRNA locus 2* | Ptr-PAL3_SNP91 | *Ptr-PAL3* | HC | AD | -31.00 | 7.49E-05 |
| Ptr-LAC35_SNP65 | *Ptr-LAC35* | Ptr-COMT22_SNP58 | *Ptr-COMT22* | HC | AA | 16.20 | 7.52E-05 |
| Ptr-bHLH15_SNP57 | *Ptr-bHLH15* | Ptr-PO85_SNP1 | *Ptr-PO85* | HC | DD | -28.70 | 7.52E-05 |
| Ptr-Myb/SANT2_SNP21 | *Ptr-Myb/SANT2* | Ptr-bHLH18_SNP111 | *Ptr-bHLH18* | HEC | DD | -23.60 | 7.59E-05 |
| Ptr-COMT25_SNP112 | *Ptr-COMT25* | L49_SNP33 | *LncRNA locus 49* | DBH | AD | 17.20 | 7.65E-05 |
| Ptr-COMT25_SNP112 | *Ptr-COMT25* | L49_SNP34 | *LncRNA locus 49* | DBH | AD | 17.20 | 7.65E-05 |
| L37_SNP18 | *LncRNA locus 37* | Ptr-PO64_SNP98 | *Ptr-PO64* | HC | DD | 25.20 | 7.66E-05 |
| L19_SNP38 | *LncRNA locus 19* | Ptr-C2H2-77_SNP70 | *Ptr-C2H2-77* | V | DA | 0.25 | 7.67E-05 |
| Ptr-COMT25_SNP109 | *Ptr-COMT25* | Ptr-C4H3_SNP108 | *Ptr-C4H3* | HC | DD | 25.10 | 7.67E-05 |
| Ptr-bZIP1_SNP38 | *Ptr-bZIP1* | Ptr-COMT22_SNP58 | *Ptr-COMT22* | HC | AA | 17.60 | 7.68E-05 |
| Ptr-HD-ZIP13_SNP16 | *Ptr-HD-ZIP13* | Ptr-bHLH21_SNP27 | *Ptr-bHLH21* | HC | AD | -17.40 | 7.69E-05 |
| Ptr-PO39_SNP87 | *Ptr-PO39* | Ptr-4CL12_SNP8 | *Ptr-4CL12* | DBH | DA | -12.20 | 7.72E-05 |
| Ptr-CCR13_SNP31 | *Ptr-CCR13* | Ptr-PAL3_SNP92 | *Ptr-PAL3* | HC | AA | -15.70 | 7.74E-05 |
| L24_SNP40 | *LncRNA locus 24* | Ptr-PO54_SNP54 | *Ptr-PO54* | LC | DD | -6.71 | 7.74E-05 |
| L37_SNP18 | *LncRNA locus 37* | Ptr-4CL21_SNP31 | *Ptr-4CL21* | V | AA | -0.28 | 7.78E-05 |
| Ptr-PO31_SNP8 | *Ptr-PO31* | Ptr-4CL21_SNP31 | *Ptr-4CL21* | CC | DD | -12.70 | 7.78E-05 |
| L37_SNP35 | *LncRNA locus 37* | Ptr-bHLH11_SNP44 | *Ptr-bHLH11* | CC | AA | -6.19 | 7.80E-05 |
| L40_SNP30 | *LncRNA locus 40* | Ptr-COMT22_SNP58 | *Ptr-COMT22* | HC | AA | -18.40 | 7.80E-05 |
| Ptr-PO40_SNP142 | *Ptr-PO40* | Ptr-4CL20_SNP103 | *Ptr-4CL20* | V | DA | 0.77 | 7.83E-05 |
| L32_SNP87 | *LncRNA locus 32* | L50_SNP108 | *LncRNA locus 50* | HC | DD | -41.30 | 7.89E-05 |
| Ptr-HD-ZIP27_SNP62 | *Ptr-HD-ZIP27* | Ptr-HD-ZIP49_SNP69 | *Ptr-HD-ZIP49* | V | AA | -0.33 | 7.90E-05 |
| Ptr-HD-ZIP27_SNP63 | *Ptr-HD-ZIP27* | Ptr-HD-ZIP49_SNP69 | *Ptr-HD-ZIP49* | V | AA | -0.33 | 7.90E-05 |
| Ptr-PO35_SNP1 | *Ptr-PO35* | Ptr-PO85_SNP1 | *Ptr-PO85* | V | DA | 0.76 | 7.90E-05 |
| Ptr-4CL20_SNP103 | *Ptr-4CL20* | Ptr-COMT22_SNP58 | *Ptr-COMT22* | HC | AA | -14.30 | 7.91E-05 |
| Ptr-PO5_SNP65 | *Ptr-PO5* | Ptr-Myb/SANT2_SNP21 | *Ptr-Myb/SANT2* | HEC | AD | -16.20 | 7.95E-05 |
| Ptr-CCR13_SNP7 | *Ptr-CCR13* | Ptr-COMT25_SNP4 | *Ptr-COMT25* | V | DA | -0.71 | 7.99E-05 |
| Ptr-HD-ZIP21_SNP40 | *Ptr-HD-ZIP21* | L33_SNP4 | *LncRNA locus 33* | V | AA | 0.30 | 8.03E-05 |
| L02_SNP11 | *LncRNA locus 2* | Ptr-4CL3_SNP45 | *Ptr-4CL3* | HC | AD | 30.80 | 8.04E-05 |
| Ptr-4CL20_SNP51 | *Ptr-4CL20* | Ptr-bHLH11_SNP44 | *Ptr-bHLH11* | HC | AA | -8.30 | 8.04E-05 |
| L19_SNP31 | *LncRNA locus 19* | L49_SNP51 | *LncRNA locus 49* | HEC | DD | -38.30 | 8.09E-05 |
| L40_SNP2 | *LncRNA locus 40* | Ptr-bHLH21_SNP26 | *Ptr-bHLH21* | V | DD | 1.37 | 8.09E-05 |
| Ptr-bHLH2_SNP16 | *Ptr-bHLH2* | Ptr-COMT22_SNP58 | *Ptr-COMT22* | HC | DA | -26.50 | 8.15E-05 |
| L19_SNP10 | *LncRNA locus 19* | Ptr-4CL4_SNP26 | *Ptr-4CL4* | HC | AA | -11.20 | 8.17E-05 |
| L15_SNP14 | *LncRNA locus 15* | Ptr-LAC22_SNP102 | *Ptr-LAC22* | DBH | DA | -6.84 | 8.24E-05 |
| L13_SNP130 | *LncRNA locus 13* | Ptr-COMT4_SNP27 | *Ptr-COMT4* | DBH | AA | -9.58 | 8.27E-05 |
| Ptr-CSE1_SNP38 | *Ptr-CSE1* | Ptr-PO40_SNP48 | *Ptr-PO40* | V | DD | -0.95 | 8.27E-05 |
| L09_SNP32 | *LncRNA locus 9* | L19_SNP19 | *LncRNA locus 19* | HC | DA | 18.90 | 8.28E-05 |
| Ptr-CCR9_SNP20 | *Ptr-CCR9* | Ptr-4CL4_SNP72 | *Ptr-4CL4* | V | DA | -0.56 | 8.29E-05 |
| Ptr-Myb/SANT2_SNP21 | *Ptr-Myb/SANT2* | Ptr-C2H2-72_SNP33 | *Ptr-C2H2-72* | HEC | DD | -26.90 | 8.29E-05 |
| Ptr-AT-Hook2_SNP2 | *Ptr-AT-Hook2* | Ptr-CCR33_SNP27 | *Ptr-CCR33* | HC | DA | -25.50 | 8.29E-05 |
| Ptr-PO43_SNP9 | *Ptr-PO43* | L49_SNP33 | *LncRNA locus 49* | HC | AA | -19.80 | 8.32E-05 |
| Ptr-PO43_SNP9 | *Ptr-PO43* | L49_SNP34 | *LncRNA locus 49* | HC | AA | -19.80 | 8.32E-05 |
| Ptr-CAD10_SNP187 | *Ptr-CAD10* | Ptr-HD-ZIP27_SNP78 | *Ptr-HD-ZIP27* | V | DA | -0.98 | 8.34E-05 |
| Ptr-bHLH18_SNP21 | *Ptr-bHLH18* | Ptr-HD-ZIP55_SNP30 | *Ptr-HD-ZIP55* | V | DD | -1.03 | 8.34E-05 |
| Ptr-HD-ZIP55_SNP30 | *Ptr-HD-ZIP55* | Ptr-PO82_SNP87 | *Ptr-PO82* | DBH | AD | -15.10 | 8.36E-05 |
| L10_SNP110 | *LncRNA locus 10* | Ptr-bHLH21_SNP26 | *Ptr-bHLH21* | HC | AD | -19.10 | 8.38E-05 |
| Pto-MiR167d_SNP30 | *Pto-MIR167d* | Ptr-4CL12_SNP28 | *Ptr-4CL12* | DBH | DD | 16.90 | 8.40E-05 |
| Ptr-PAL3_SNP107 | *Ptr-PAL3* | Ptr-CCR28_SNP94 | *Ptr-CCR28* | FW | AD | 8.39 | 8.42E-05 |
| Ptr-PO40_SNP142 | *Ptr-PO40* | Ptr-CCR9_SNP119 | *Ptr-CCR9* | CC | DA | -22.20 | 8.42E-05 |
| Ptr-COMT25_SNP110 | *Ptr-COMT25* | L42_SNP3 | *LncRNA locus 42* | HC | AA | -8.05 | 8.47E-05 |
| Ptr-CCR13_SNP7 | *Ptr-CCR13* | Ptr-PAL2_SNP46 | *Ptr-PAL2* | CC | DA | -16.40 | 8.50E-05 |
| Ptr-PAL3_SNP107 | *Ptr-PAL3* | Ptr-bHLH15_SNP56 | *Ptr-bHLH15* | FL | AD | 0.16 | 8.55E-05 |
| Ptr-HCT1_SNP28 | *Ptr-HCT1* | Ptr-CAD16_SNP98 | *Ptr-CAD16* | DBH | AA | 10.00 | 8.58E-05 |
| L09_SNP63 | *LncRNA locus 9* | Ptr-HD-ZIP13_SNP18 | *Ptr-HD-ZIP13* | DBH | DD | -26.10 | 8.58E-05 |
| Ptr-PO17_SNP20 | *Ptr-PO17* | Ptr-LAC2_SNP20 | *Ptr-LAC2* | V | AA | 0.76 | 8.58E-05 |
| L19_SNP67 | *LncRNA locus 19* | Ptr-HD-ZIP21_SNP40 | *Ptr-HD-ZIP21* | DBH | AA | 3.30 | 8.62E-05 |
| L19_SNP68 | *LncRNA locus 19* | Ptr-HD-ZIP21_SNP40 | *Ptr-HD-ZIP21* | DBH | AA | 3.30 | 8.62E-05 |
| Ptr-PO25_SNP30 | *Ptr-PO25* | L37_SNP4 | *LncRNA locus 37* | V | AD | -0.77 | 8.64E-05 |
| Ptr-PO90_SNP54 | *Ptr-PO90* | L68_SNP1 | *LncRNA locus 68* | HC | DA | 19.60 | 8.65E-05 |
| L40_SNP30 | *LncRNA locus 40* | Ptr-bHLH18_SNP21 | *Ptr-bHLH18* | HC | DA | -28.90 | 8.70E-05 |
| Ptr-COMT25_SNP153 | *Ptr-COMT25* | Ptr-C4H2_SNP4 | *Ptr-C4H2* | LC | AD | -2.31 | 8.73E-05 |
| L49_SNP33 | *LncRNA locus 49* | Ptr-4CL4_SNP70 | *Ptr-4CL4* | V | DA | -1.34 | 8.76E-05 |
| L49_SNP34 | *LncRNA locus 49* | Ptr-4CL4_SNP70 | *Ptr-4CL4* | V | DA | -1.34 | 8.76E-05 |
| L49_SNP33 | *LncRNA locus 49* | Ptr-4CL4_SNP71 | *Ptr-4CL4* | V | DA | -1.34 | 8.76E-05 |
| L49_SNP34 | *LncRNA locus 49* | Ptr-4CL4_SNP71 | *Ptr-4CL4* | V | DA | -1.34 | 8.76E-05 |
| L40_SNP2 | *LncRNA locus 40* | Ptr-bHLH21_SNP27 | *Ptr-bHLH21* | V | DD | 1.36 | 8.81E-05 |
| Ptr-COMT25_SNP110 | *Ptr-COMT25* | Ptr-bZIP4_SNP135 | *Ptr-bZIP4* | HEC | AA | -12.50 | 8.81E-05 |
| L50_SNP110 | *LncRNA locus 50* | Ptr-CCR9_SNP92 | *Ptr-CCR9* | DBH | AD | 14.60 | 8.81E-05 |
| Ptr-HD-ZIP13_SNP15 | *Ptr-HD-ZIP13* | Ptr-C2H2-77_SNP57 | *Ptr-C2H2-77* | HC | AA | -8.06 | 8.84E-05 |
| Ptr-CCR13_SNP7 | *Ptr-CCR13* | Ptr-PO82_SNP100 | *Ptr-PO82* | V | DA | -0.39 | 8.88E-05 |
| Ptr-CAD14_SNP81 | *Ptr-CAD14* | Ptr-HCT13_SNP37 | *Ptr-HCT13* | V | DA | 1.10 | 8.92E-05 |
| L10_SNP110 | *LncRNA locus 10* | Ptr-bHLH21_SNP27 | *Ptr-bHLH21* | HC | AD | -19.30 | 8.94E-05 |
| Ptr-CAD10_SNP187 | *Ptr-CAD10* | Ptr-PO72_SNP18 | *Ptr-PO72* | HC | DD | 24.60 | 8.96E-05 |
| L06_SNP61 | *LncRNA locus 6* | Ptr-CCR8_SNP10 | *Ptr-CCR8* | V | DA | -0.71 | 8.98E-05 |
| Ptr-CAD10_SNP170 | *Ptr-CAD10* | Ptr-PO40_SNP142 | *Ptr-PO40* | V | AD | -0.90 | 8.99E-05 |
| L02_SNP13 | *LncRNA locus 2* | Ptr-bHLH21_SNP38 | *Ptr-bHLH21* | HC | AA | 20.00 | 9.04E-05 |
| Ptr-CCR13_SNP7 | *Ptr-CCR13* | L13_SNP145 | *LncRNA locus 13* | V | DA | 0.59 | 9.07E-05 |
| L49_SNP33 | *LncRNA locus 49* | Ptr-4CL4_SNP72 | *Ptr-4CL4* | V | DA | 1.34 | 9.09E-05 |
| L49_SNP34 | *LncRNA locus 49* | Ptr-4CL4_SNP72 | *Ptr-4CL4* | V | DA | 1.34 | 9.09E-05 |
| L09_SNP32 | *LncRNA locus 9* | Ptr-HCT12_SNP8 | *Ptr-HCT12* | HC | AA | -9.12 | 9.11E-05 |
| L02_SNP13 | *LncRNA locus 2* | Ptr-CAD10_SNP198 | *Ptr-CAD10* | HC | AA | 22.30 | 9.14E-05 |
| L30_SNP18 | *LncRNA locus 30* | Ptr-bHLH10_SNP39 | *Ptr-bHLH10* | V | AD | -0.64 | 9.16E-05 |
| Ptr-4CL20_SNP93 | *Ptr-4CL20* | Ptr-C2H2-72_SNP61 | *Ptr-C2H2-72* | V | AD | -1.24 | 9.21E-05 |
| Ptr-HD-ZIP13_SNP31 | *Ptr-HD-ZIP13* | Ptr-HD-ZIP55_SNP2 | *Ptr-HD-ZIP55* | HC | AA | -15.80 | 9.23E-05 |
| Ptr-HD-ZIP13_SNP32 | *Ptr-HD-ZIP13* | Ptr-HD-ZIP55_SNP2 | *Ptr-HD-ZIP55* | HC | AA | -15.80 | 9.23E-05 |
| Ptr-COMT25_SNP119 | *Ptr-COMT25* | L49_SNP33 | *LncRNA locus 49* | V | DD | -1.77 | 9.26E-05 |
| Ptr-COMT25_SNP119 | *Ptr-COMT25* | L49_SNP34 | *LncRNA locus 49* | V | DD | -1.77 | 9.26E-05 |
| Ptr-4CL9_SNP104 | *Ptr-4CL9* | L62_SNP113 | *LncRNA locus 62* | CC | DD | 19.90 | 9.26E-05 |
| Ptr-HD-ZIP4_SNP11 | *Ptr-HD-ZIP4* | Ptr-PO72_SNP10 | *Ptr-PO72* | V | AD | -0.54 | 9.28E-05 |
| Ptr-PO23_SNP6 | *Ptr-PO23* | Ptr-bHLH15_SNP56 | *Ptr-bHLH15* | HC | AA | -11.30 | 9.30E-05 |
| Ptr-CCR13_SNP7 | *Ptr-CCR13* | Ptr-CCoAOMT4_SNP22 | *Ptr-CCoAOMT4* | V | AA | -0.25 | 9.30E-05 |
| Ptr-CAD14_SNP81 | *Ptr-CAD14* | Ptr-TBP1_SNP17 | *Ptr-TBP1* | V | DA | -1.13 | 9.31E-05 |
| Ptr-HD-ZIP13_SNP31 | *Ptr-HD-ZIP13* | Ptr-COMT12_SNP120 | *Ptr-COMT12* | HC | AA | -15.10 | 9.42E-05 |
| Ptr-HD-ZIP13_SNP32 | *Ptr-HD-ZIP13* | Ptr-COMT12_SNP120 | *Ptr-COMT12* | HC | AA | -15.10 | 9.42E-05 |
| L09_SNP54 | *LncRNA locus 9* | Ptr-PO13_SNP8 | *Ptr-PO13* | HC | AA | -9.75 | 9.44E-05 |
| Ptr-PO23_SNP8 | *Ptr-PO23* | Ptr-CCR33_SNP25 | *Ptr-CCR33* | HEC | AA | 10.50 | 9.48E-05 |
| Ptr-CCoAOMT5_SNP20 | *Ptr-CCoAOMT5* | Ptr-C4H3_SNP108 | *Ptr-C4H3* | HC | AA | -16.10 | 9.56E-05 |
| Ptr-HD-ZIP27_SNP78 | *Ptr-HD-ZIP27* | Ptr-HCT12_SNP9 | *Ptr-HCT12* | V | AA | 0.76 | 9.58E-05 |
| Ptr-PO70_SNP64 | *Ptr-PO70* | Ptr-PO85_SNP50 | *Ptr-PO85* | V | DD | -1.12 | 9.58E-05 |
| Ptr-COMT25_SNP109 | *Ptr-COMT25* | Ptr-bZIP4_SNP135 | *Ptr-bZIP4* | HEC | AA | -12.50 | 9.61E-05 |
| Ptr-PO88_SNP33 | *Ptr-PO88* | Ptr-HD-ZIP49_SNP69 | *Ptr-HD-ZIP49* | CC | AD | -19.00 | 9.61E-05 |
| L19_SNP138 | *LncRNA locus 19* | Ptr-HCT8_SNP80 | *Ptr-HCT8* | HC | AA | -9.55 | 9.67E-05 |
| L43_SNP59 | *LncRNA locus 43* | Ptr-HCT13_SNP2 | *Ptr-HCT13* | V | DD | -0.97 | 9.76E-05 |
| Ptr-CAD10_SNP198 | *Ptr-CAD10* | Ptr-PO40_SNP142 | *Ptr-PO40* | HC | DD | -30.10 | 9.76E-05 |
| Ptr-C2H2-77_SNP70 | *Ptr-C2H2-77* | Ptr-HD-ZIP51_SNP14 | *Ptr-HD-ZIP51* | V | AA | 0.24 | 9.79E-05 |
| Ptr-PAL3_SNP102 | *Ptr-PAL3* | Ptr-PO17_SNP54 | *Ptr-PO17* | V | AA | -0.88 | 9.82E-05 |
| Ptr-PO70_SNP63 | *Ptr-PO70* | Ptr-AT-Hook4_SNP56 | *Ptr-AT-Hook4* | V | DD | -1.53 | 9.85E-05 |
| Ptr-PO17_SNP28 | *Ptr-PO17* | Ptr-COMT22_SNP58 | *Ptr-COMT22* | HC | AA | 13.20 | 9.86E-05 |
| L43_SNP59 | *LncRNA locus 43* | Ptr-HD-ZIP51_SNP85 | *Ptr-HD-ZIP51* | V | DD | 1.78 | 9.92E-05 |
| L35_SNP13 | *LncRNA locus 35* | Ptr-LAC43_SNP28 | *Ptr-LAC43* | HC | DA | -18.70 | 9.93E-05 |
| Ptr-TBP1_SNP17 | *Ptr-TBP1* | Ptr-4CL4_SNP72 | *Ptr-4CL4* | V | AA | -0.38 | 9.96E-05 |
| Ptr-HCT8_SNP58 | *Ptr-HCT8* | L40_SNP2 | *LncRNA locus 40* | V | AD | 1.01 | 9.97E-05 |
| Ptr-HCT6_SNP1 | *Ptr-HCT6* | Ptr-Myb/SANT2_SNP21 | *Ptr-Myb/SANT2* | HEC | DD | -22.30 | 9.98E-05 |
| Ptr-COMT25_SNP95 | *Ptr-COMT25* | Ptr-PO39_SNP87 | *Ptr-PO39* | DBH | AD | -9.82 | 9.99E-05 |
| Ptr-COMT9_SNP7 | *Ptr-COMT9* | Ptr-PO85_SNP1 | *Ptr-PO85* | HC | DA | 14.90 | 9.99E-05 |

**Table S7.** Details of eQTNs identified for each gene in the lignin biosynthesis pathway.

| **Expression Traits** | **Total number of associated eQTNs** | **The source of eQTNs** | | | | ***R*^2^** |
| --- | --- | --- | --- | --- | --- | --- |
|  |  | **TF gene** | **Lignin biosyhthetic gene** | **MiRNA gene** | **LncRNA loci** |  |
| Ptr-4CL14 | 2 | 0 | 1 | 0 | 0 | 19.69%-27.31% |
| Ptr-4CL3 | 48 | 0 | 17 | 23 | 0 | 13.86%-33.76% |
| Ptr-4CL9 | 6 | 0 | 1 | 3 | 0 | 21.56%-26.07% |
| Ptr-C3H3 | 12 | 0 | 4 | 4 | 0 | 22.27%-33.44% |
| Ptr-C4H3 | 68 | 1 | 16 | 36 | 2 | 17.52%-34.83% |
| Ptr-CAD1 | 121 | 0 | 14 | 70 | 2 | 21.56%-28.39% |
| Ptr-CAD10 | 164 | 0 | 27 | 114 | 4 | 17.66%-33.87% |
| Ptr-CAD12 | 2 | 0 | 0 | 1 | 0 | 22.02%-22.75% |
| Ptr-CAD16 | 548 | 0 | 115 | 311 | 7 | 17.51%-35.00% |
| Ptr-CAD5 | 7 | 0 | 3 | 4 | 0 | 22.05%-28.08% |
| Ptr-CAD8 | 1 | 0 | 1 | 0 | 0 | 23.05%-23.05% |
| Ptr-CCoAOMT1 | 3 | 0 | 2 | 0 | 0 | 23.98%-32.21% |
| Ptr-CCoAOMT5 | 447 | 0 | 78 | 296 | 4 | 17.54%-34.85% |
| Ptr-CCR10 | 1 | 0 | 0 | 1 | 0 | 23.43%-23.43% |
| Ptr-CCR2 | 3 | 0 | 0 | 3 | 0 | 23.28%-28.89% |
| Ptr-CCR28 | 5 | 0 | 1 | 4 | 0 | 26.86%-32.41% |
| Ptr-CCR29 | 7611 | 9 | 1889 | 4079 | 206 | 17.50%-35.00% |
| Ptr-CCR30 | 88 | 0 | 2 | 73 | 5 | 22.07%-33.95% |
| Ptr-CCR33 | 5097 | 4 | 1371 | 2584 | 129 | 17.51%-35.00% |
| Ptr-CCR8 | 11 | 0 | 1 | 10 | 0 | 22.24%-31.30% |
| Ptr-COMT1 | 135 | 1 | 26 | 81 | 4 | 18.00%-34.89% |
| Ptr-COMT13 | 413 | 4 | 88 | 229 | 1 | 17.61%-34.91% |
| Ptr-COMT2 | 206 | 0 | 84 | 102 | 3 | 17.53%-34.69% |
| Ptr-COMT25 | 318 | 1 | 63 | 172 | 10 | 17.63%-34.93% |
| Ptr-COMT30 | 2666 | 9 | 646 | 1432 | 79 | 17.52%-34.98% |
| Ptr-COMT34 | 1 | 0 | 1 | 0 | 0 | 21.83%-21.83% |
| Ptr-F5H2 | 208 | 0 | 83 | 105 | 5 | 18.22%-34.39% |
| Ptr-HCT12 | 327 | 1 | 54 | 181 | 5 | 22.21%-32.94% |
| Ptr-HCT6 | 2 | 0 | 0 | 0 | 0 | 28.43%-29.99% |
| Ptr-LAC25 | 7 | 0 | 2 | 3 | 1 | 19.35%-34.53% |
| Ptr-LAC27 | 67 | 4 | 10 | 44 | 3 | 19.76%-33.63% |
| Ptr-LAC33 | 109 | 0 | 10 | 58 | 2 | 22.06%-32.84% |
| Ptr-PAL1 | 1 | 0 | 1 | 0 | 0 | 22.16%-22.16% |
| Ptr-PAL2 | 191 | 0 | 74 | 97 | 3 | 27.18%-34.15% |
| Ptr-PAL4 | 1 | 0 | 0 | 0 | 0 | 25.66%-25.66% |
| Ptr-PO22 | 1 | 0 | 0 | 1 | 0 | 29.59%-29.59% |
| Ptr-PO28 | 1 | 0 | 1 | 0 | 0 | 23.11%-23.11% |
| Ptr-PO33 | 6 | 0 | 1 | 1 | 0 | 22.56%-30.45% |
| Ptr-PO43 | 14 | 0 | 0 | 13 | 0 | 21.90%-30.41% |
| Ptr-PO46 | 47 | 0 | 13 | 30 | 0 | 22.05%-32.48% |
| Ptr-PO54 | 1587 | 2 | 419 | 806 | 46 | 17.57%-34.98% |
| Ptr-PO64 | 5 | 0 | 0 | 4 | 0 | 20.53%-24.99% |
| Total | 20558 | 36 | 5119 | 10975 | 521 | 13.86%-35.00% |

**Table S8.** Phenotypic variation of ten growth and wood property traits in the association population of *P. tomentosa*.

| **Statistics** | **Lignin content (%)** | **Holocellulose content (%)** | **α-cellulose content (%)** | **Hemicellulose content (%)** | **Fibre length (mm)** | **Fiber width (μm)** | **Microfibril angle (°)** | **Diameter at breast height (cm)** | **Tree height (m)** | **Stem volume (m^3^)** |
| --- | --- | --- | --- | --- | --- | --- | --- | --- | --- | --- |
| Minimum value | 16.52 | 36.40 | 12.34 | 5.55 | 0.87 | 17.78 | 11.49 | 10.19 | 9.80 | 51.99 |
| Maximum value | 28.67 | 95.40 | 55.41 | 56.16 | 1.39 | 29.85 | 30.12 | 38.17 | 21.10 | 402.69 |
| Mean value | 21.26 | 72.28 | 40.14 | 32.14 | 1.17 | 23.12 | 17.56 | 21.90 | 14.80 | 166.93 |
| Fold | 1.74 | 2.62 | 4.49 | 10.12 | 1.60 | 1.68 | 2.62 | 3.75 | 2.15 | 7.75 |
| SD^†^ | 2.32 | 9.61 | 8.68 | 8.39 | 0.08 | 2.09 | 3.90 | 5.73 | 2.62 | 65.97 |
| SE^‡^ | 0.0053 | 0.0221 | 0.0200 | 0.0193 | 0.0002 | 0.0048 | 0.0090 | 0.0132 | 0.0060 | 0.1516 |
| C.V.(%)^§^ | 10.90 | 13.29 | 21.62 | 26.09 | 6.76 | 9.03 | 22.19 | 26.17 | 17.71 | 39.52 |

^†^Standard deviation for phenotypic variation

^‡^Standard error for phenotypic variation

^§^Coefficient of phenotypic variation

**Table S9.** Phenotypic correlations for tree growth and wood property traits in the association population of *P. tomentosa*.

|  | **Lignin content** | **Holocellulose content** | **α-cellulose content** | **Hemicellulose content** | **Fiber length** | **Fiber width** | **Microfibril angle** | **Diameter at breast height** | **Tree height** | **Stem volume** |
| --- | --- | --- | --- | --- | --- | --- | --- | --- | --- | --- |
| Lignin content |  | -0.278** | -0.179** | -0.134** | -0.146** | 0.027 | 0.187** | 0.072 | 0.005 | 0.062 |
| Holocellulose content | -0.278** |  | 0.584** | 0.542** | -0.038 | -0.063 | -0.136** | -0.310** | -0.270** | -0.351** |
| α-cellulose content | -0.179** | 0.584** |  | -0.366** | -0.012 | -0.103* | -0.016 | -0.445** | -0.300** | -0.488** |
| Hemicellulose content | -0.134** | 0.542** | -0.366** |  | -0.031 | 0.035 | -0.139** | 0.109* | 0.002 | 0.107* |
| Fibre length | -0.146** | -0.038 | -0.012 | -0.031 |  | 0.202** | -0.339** | 0.290** | 0.312** | 0.255** |
| Fiber width | 0.027 | -0.063 | -0.103* | 0.035 | 0.202** |  | -0.297** | 0.115* | -0.011 | -0.010 |
| Microfibril angle | 0.187** | -0.136** | -0.016 | -0.139** | -0.339** | -0.297** |  | -0.272** | -0.352** | -0.194** |
| Diameter at breast height | 0.072 | -0.310** | -0.445** | 0.109* | 0.290** | 0.115* | -0.272** |  | 0.651** | 0.937** |
| Tree height | 0.005 | -0.270** | -0.300** | 0.002 | 0.312** | -0.011 | -0.352** | 0.651** |  | 0.762** |
| Stem volume | 0.062 | -0.351** | -0.488** | 0.107* | 0.255** | -0.010 | -0.194** | 0.937** | 0.762** |  |

Negative correlations are marked by "−" ; **P* < 0.05 level of significance; ** *P* < 0.01 level of significance
